# Identification of oligo-adenylated small RNAs in the parasite *Entamoeba* and a potential role for small RNA control

**DOI:** 10.1186/s12864-020-07275-6

**Published:** 2020-12-09

**Authors:** Hanbang Zhang, Gretchen M. Ehrenkaufer, Neil Hall, Upinder Singh

**Affiliations:** 1 Division of Infectious Diseases, Department of Internal Medicine, Stanford University School of Medicine, S-143 Grant Building, 300 Pasteur Drive, Stanford, CA 94305-5107 USA; 2 Earlham Institute, Norwich Research Park, Norwich, NR4 7UH UK; 3 Department of Microbiology and Immunology, Stanford University School of Medicine, Stanford, California 94305-5107 USA

**Keywords:** RNAi, Small RNA, Small RNA sequencing, Argonaute, Small RNA oligo-adenylation, Parasite

## Abstract

**Background:**

The RNA interference (RNAi) pathway is a gene regulation mechanism that utilizes small RNA (sRNA) and Argonaute (Ago) proteins to silence target genes. Our previous work identified a functional RNAi pathway in the protozoan parasite *Entamoeba histolytica*, including abundant 27 nt antisense sRNA populations which associate with *Eh*Ago2–2 protein. However, there is lack of understanding about the sRNAs that are bound to two other *Eh*Agos *(Eh*Ago2–1 and 2–3), and the mechanism of sRNA regulation itself is unclear in this parasite. Therefore, identification of the entire pool of sRNA species and their sub-populations that associate with each individual *Eh*Ago protein would be a major step forward.

**Results:**

In the present study, we sequenced sRNA libraries from both total RNAs and *Eh*Ago bound RNAs. We identified a new population of 31 nt sRNAs that results from the addition of a non-templated 3–4 adenosine nucleotides at the 3′-end of the 27 nt sRNAs, indicating a non-templated RNA-tailing event in the parasite. The relative abundance of these two sRNA populations is linked to the efficacy of gene silencing for the target gene when parasites are transfected with an RNAi-trigger construct, indicating that non-templated sRNA-tailing likely play a role in sRNA regulation in this parasite. We found that both sRNA populations (27 nt and 31 nt) are present in the related parasite *Entamoeba invadens*, and are unchanged during the development. In sequencing the sRNAs associating with the three *Eh*Ago proteins, we observed that despite distinct cellular localization, all three *Eh*Ago sRNA libraries contain 27 nt sRNAs with 5′-polyphosphate (5′-polyP) structure and share a largely overlapping sRNA repertoire. In addition, our data showed that a fraction of 31 nt sRNAs associate with *Eh*Ago2–2 but not with its mutant protein (C-terminal deletion), nor other two *Eh*Agos, indicating a specific *Eh*Ago site may be required for sRNA modification process in the parasite.

**Conclusion:**

We identified a new population of sRNA with non-templated oligo-adenylation modification, which is the first such observation amongst single celled protozoan parasites. Our sRNA sequencing libraries provide the first comprehensive sRNA dataset for all three *Entamoeba* Ago proteins, which can serve as a useful database for the amoeba community.

**Supplementary Information:**

The online version contains supplementary material available at 10.1186/s12864-020-07275-6.

## Background

Thought to be evolved from an ancient anti-viral defense mechanism, RNA interference (RNAi) and its gene regulation pathways are conserved in most eukaryotic organisms [1–3]. RNAi can be triggered by double-stranded RNA (dsRNA), which is processed by Dicer into short interfering RNA (siRNA) duplexes. One strand of the siRNA duplex is then loaded into Argonaute (Ago) protein to form the RNA induced silencing complex (RISC). The RISC leads to the inactivation of target mRNAs through mechanisms of posttranscriptional or transcriptional gene silencing (PTGS/TGS) [4]. In addition to the above mentioned RNAi pathway, an amplified gene silencing mechanism involving the activity of RNA-dependent RNA polymerases (RdRPs) has been identified in plants, nematodes and fungi [5, 6]. RdRPs respond to a primary sRNA pool and generate secondary sRNAs. In plants and fungi, RdRPs use the cleaved mRNA as template to synthesize dsRNA which are then processed by Dicer to generate secondary sRNAs [5]. In *Caenorhabditis elegans*, RdRPs synthesize secondary sRNAs de novo. These secondary sRNAs are called 22G sRNAs for being 22 nt in size and with antisense orientation, having both 5′-end triphosphate structure and guanosine bias [7].

There are different classes of sRNAs including siRNAs, miRNAs, and Piwi-interacting RNAs (piRNAs) [8]. Recent studies have shown that sRNAs are modified for different cellular functions [9, 10]. Uridylation of siRNAs and piRNAs is observed in many systems including *Chlamydomonas* [11], *C. elegans* [12], *Arabidopsis* [13], *Drosophila* [14], and mammalian cells [15, 16]. Mono-adenylation is reported to have a stabilizing effect on mature miRNAs, and miRNA precursors are often modified by uridylation for degradation [14]. In fission yeast, a fraction of Argonaute-bound siRNAs are found with non-templated adenosines at the 3′-end [17]. Thus, sRNA modification with non-templated uridine(s) or adenosine(s) among these model organisms are used as a mechanism for regulating sRNAs: either leading to sRNA degradation (uridylation), miRNA protection (mono-adenylation) or sRNA turnover in fission yeast (di-adenylation and di-uridylation).

The protozoan parasite *E. histolytica* causes amebiasis, a major health concern in underdeveloped countries [18, 19]. The parasite has two life stages: a dormant, environmentally resistant cyst form and a proliferative trophozoite form, which is capable of causing invasive disease. Our previous work has identified a functional RNAi pathway in this parasite [20–23]. We found that *E. histolytica* has abundant 27 nt sRNAs with a 5′-polyP structure, a feature that is seen in the secondary sRNAs in *C. elegans* and nematode parasite *Ascaris suum* [7, 24]. There are three *Eh*Ago proteins: *Eh*Ago2–1 (EHI_186850), *Eh*Ago2–2 (EHI_125650), and *Eh*Ago2–3 (EHI_177170) that have distinct subcellular locations including the nucleus (*Eh*Ago2–2), perinuclear ring (*Eh*Ago2–1, *Eh*Ago2–3), and cytosol (*Eh*Ago2–3) [25]. Our structural domain analysis showed that all three *Eh*Agos have a conserved PAZ and PIWI domain [25]. We demonstrated that *Eh*Ago PAZ domains are essential for sRNA binding for all three *Eh*Agos, and sRNA binding affects cellular localization of *Eh*Ago2–1 and *Eh*Ago2–3 but not *Eh*Ago2–2 [25]. To better understand the RNAi mechanism(s) in this parasite, we ask three questions (i) what are the full spectrum of sRNA species in this parasite? (ii) do the three *Eh*Agos bind different sRNA sub-populations? (iii) are sRNAs themselves regulated in *Entamoeba*?

In this report, we performed high throughput sRNA sequencing for size-fractionated total RNAs and three *Eh*Ago-bound sRNAs. We demonstrated that two dominant sRNA populations are present: one at 27 nt and the other at 31 nt, with the latter containing non-templated 3–4 adenosines at the 3**′**-end, indicating an oligo-adenylation modification event of the sRNAs in the *E. histolytica* parasite. We further expanded our sRNA sequencing effort for 31 nt populations in the related reptilian parasite *E. invadens*, and found that the 31 nt sRNA populations are not changed during development. Using an RNAi-trigger gene silencing approach, we showed that the relative abundance of the two sRNA populations is reversed when a target gene is unable to be silenced. Sequencing of three *Eh*Ago immunoprecipitation (IP) RNA libraries showed significant overlap of sRNA species, mainly targeting retrotransposons and ~ 226 genes that are silenced in this organism. We also found that there is a fraction of 31 nt sRNA reads that are in the *Eh*Ago2–2 IP library but not in its mutant, nor the other two *Eh*Agos IP libraries. Overall, our study provides the first comprehensive dataset for sRNAs bound to the three *Eh*Ago proteins, which can serve as a useful database for the *Entamoeba* community. The finding of sRNAs with oligo-adenylation revealed an additional layer of sRNA regulation control and functional diversity in this single celled deep-branching eukaryotic pathogen.

## Results

### Two small RNA populations (27 nt and 31 nt) are identified in *Entamoeba*

In order to identify the complete spectrum of sRNA species in *Entamoeba*, including those that may have diverse structures, modifications, or may be less abundant, we decided to extensively explore the endogenous sRNA populations in *E. histolytica* by sequencing total RNA fractions (15-45 nt) from wildtype *E. histolytica* trophozoites. We fractionated the total RNA into two RNA size fractions (15-30 nt and 30-45 nt). The recovered RNAs from both fractions were cloned by 5′-P independent cloning method (using tobacco acid pyrophosphatase (TAP) to convert 5′-polyP into 5′-monoP) (Suppl. Table 1). Although we previously reported similar sRNA libraries, those libraries were on a small-scale sequencing level using Sanger sequencing or pyrosequencing approaches, and sRNAs identified were in the 15-30 nt range [21, 23]. The goal in this study is to provide a full account of *Entamoeba* sRNAs, including potential sRNA species with modifications, using the current Illumina deep sequencing platform.

**Fig. 1:**
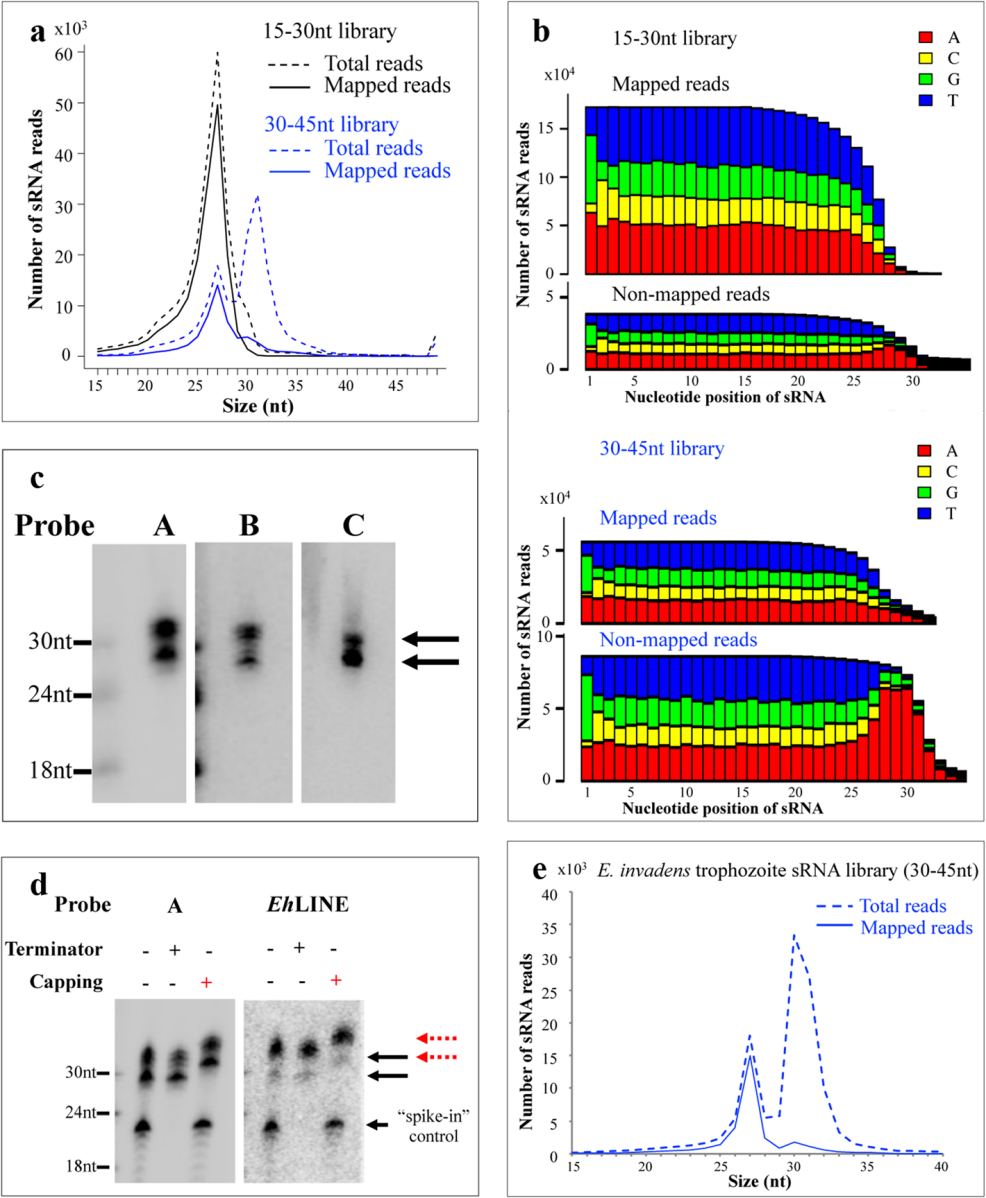
*E. histolytica* has two sRNA populations (27 nt and 31 nt) and 31 nt sRNA is oligo-A modified at 3′-end. **a** Size distribution for two size-fractionated sRNA libraries (15-30 nt and 30-45 nt). Both libraries were cloned using 5′-P independent cloning method (TAP). Total reads (dashed lines) and mapped reads (solid lines) are shown. The 15-30 nt size-fractionated library shows a single peak at 27 nt for both total and mapped reads. The 30-45 nt size-fractionated library has two sRNA peaks (27 nt and 31 nt) for the total reads, and only the 27 nt sRNAs, but not 31 nt sRNAs, can be mapped to genome. **b** Nucleotide distribution analysis for the mapped and non-mapped reads**.** There is a 5**′**-G bias for the first nucleotide in all populations. The 30–45 non-mapped reads show a 5**′**-G bias for the first nucleotide, and a string of 3 or 4 As are identified at 3**′**-end. After trimming of the 3**′**-end As, these reads can be remapped to the genome (Table 1) indicating non-templated oligo-A tailing event to the 27 nt sRNAs. **c** Northern blot detects both 27 nt and 31 nt sRNA populations. Three sRNAs (probes called A, B and C) were cloned in both size sRNA populations; Northern blot analysis detected signals at both sRNA sizes, indicating that the two sRNA species co-exist in the cell. A sRNA enriched RNA from *E. histolytica* trophozoites (20 μg) was used for each sample and probed with end-labeled [^32^P] oligonucleotide probes corresponding to the cloned sRNAs, see Suppl. Table 7 and Suppl. original blot for Fig. 1**c**. **d** Both 27 nt and 31 nt sRNA populations are resistant to cleavage by Terminator enzyme, they are shifted for one nucleotide distance via capping assay, indicating a 5′-polyP structure for both sRNA populations. A “spike-in” control of 22 nt RNA with a 5′-monoP is readily degraded by Terminator enzyme. An increase in size following treatment with capping enzyme indicates that RNAs have 5′-di- or tri-phosphate structure. The probe “A” and a probe specific to *Eh*LINE were used (Suppl. Table 7 and Suppl. original blot for Fig. 1**d**.) **e** *E. invadens* 30-45 nt size-fractionated library has two sRNA peaks. Similar to *E. histolytica*, the 27 nt peak can be mapped to the genome but not 31 nt peak. The plot shows the trophozoite dataset (similar plots are observed for two other time point datasets, data not shown)

The sRNA size distribution of the two libraries (15-30 nt and 30-45 nt libraries) were cloned by TAP method, as shown in Fig. 1a. We observed only one sRNA population (a sharp 27 nt peak) for the 15-30 nt library, which matched with previous results [23]. However, for the 30-45 nt library, we identified two sRNA populations (peaks at 27 nt and 31 nt). The 27 nt peak is likely a carry-over from the abundant 27 nt population, but the peak at 31 nt was unexpected and new to us. We characterized and mapped the sRNA sequences from both libraries using a custom data processing pipeline (Suppl. Fig. 1). The unique reads were mapped to tRNA and rDNA sequences using Bowtie [26]; the remaining reads were aligned to the amebic *Eh*LINEs (Long Interspersed Nuclear Elements), the genome, and transcriptome. This analysis revealed that most reads in 27 nt peak can be mapped to the genome. The sRNA reads in the 31 nt peak did not map to the genome (Table 1 and Fig. 1a). To understand why the 31 nt sRNAs could not be mapped to the genome, we plotted the nucleotide frequency at each position for the non-mapped reads and identified an oligo-A tail prominent at the 3**′**-end as the reason for these reads being not mapped to the genome (Fig. 1b). In order to map the 31 nt sRNA reads, we clipped the sequence reads after the 27 nt position using a custom Python script, then re-mapped to the genome. Our analysis revealed that these clipped sequences can now map to the genome, indicating that the non-templated 3–4 As were added to the existing 27 nt sRNAs (Table 1).

**Table 1:** Genomic categories that are mapped by sRNA reads by size-fractionated total RNA libraries (TAP cloning method)

	Size-fractionated total RNA libraries, 5′ P-independent cloning, WT		
Categories	15-30nt (27nt sRNAs)	30-45nt ^#^ (27nt and 31nt sRNAs)	3'-end trimmed ^*^ (31nt sRNAs trimmed off oligo-As)
Total reads	624,954	411,419	
Unique (unique/total reads)	242,277 (38.8%)	171,309 (41.6%)	86,031
tRNA^a,S^	2,343 (1.0%)	2,848 (1.7%)	1,035 (1.2%)
rRNA^a,S^	12,957 (5.3%)	18,235 (10.6%)	450 (0.5%)
LINEs^a,AS^	16,424 (6.8%)	8,492 (5.0%)	**23,999 (27.9%)**
Map to rest of genome^a^	172,261 (71.1%)	55,703 (32.5%)	36,651 (42.6%)
Map to predicted ORFs^a,AS^	124,770 (51.5%)	37,145 (21.7%)	27,508 (32%)
Not mapped to genome (%)	38,292 (15.8%)	86,031 (50.2%)	23,896 (27.8%)

^a^ Number of unique reads divided by total unique reads

^S^ Most reads are in sense orientation

^AS^ Most reads are in antisense orientation

^#^ Only 27nt sRNAs can be mapped to the genome, not 31nt sRNAs

^*^ The 31nt sRNAs (non-mapped reads in 30-45nt library) were trimmed from 3' end into 27nt size, then they can be mapped to the genome.

We noticed that the reads that map to tRNA and rRNA are predominantly in the sense orientation. The tRNA and rRNA reads are inevitably present in almost all published sRNA sequencing libraries. These tRNA and rRNA reads are often considered partial degradation products as they are highly abundant in the cell, with a few exceptions [27, 28]. Of note, the number of reads that map to rRNA is significantly less in the category of 31 nt sRNAs (Table 1, 0.5% for 3**′**-end trimmed), compared to the non-modified 27 nt sRNAs (Table 1, 5.3% for 15-30 nt library and 10.6% for 30-45 nt library).

The *E. histolytica* genome is highly populated with retrotransposons and repeat elements including *Eh*LINEs, *Eh*SINEs (Short Interspersed Nuclear Elements), and EREs (*Entamoeba* Repeat Elements) [29]. There are thousands of copies of *Eh*LINEs in the genome, but they are considered “inactive”. Genome sequencing has not identified any *Eh*LINEs which have completed open reading frames (ORF) [30]. Interestingly, reads mapped to *Eh*LINEs make up almost 28% of these 31 nt sRNAs compared with < 10% in the 27 nt sRNAs (the non-modified populations), indicating a possible link between retrotransposon control and sRNA modification in the parasite.

In order to determine if the sRNA species overlap between the 27 nt and 31 nt populations, we performed alignment analysis of the trimmed 31 nt sRNAs directly against the 27 nt sRNA reads. We found that most (85%) of the trimmed 31 nt reads can be mapped to the 27 nt reads, indicating a high overlap between two sRNA populations. Consequently, we found that both sRNA populations target almost the same set of genes, and the number of unique sRNAs mapped to these genes from both datasets are correlated (Suppl. Fig. 2A). However, the abundance of individual sRNA cloned in each population is not well correlated (Suppl. Fig. 2B), indicating that the abundance level of sRNAs within the two sRNA populations may be regulated differently within the cell.

We selected a few sRNAs which were cloned in both the 27 nt and 31 nt populations and designed probes based on the sequences of these chosen sRNAs. We detected the two expected sRNA sizes by Northern blot analysis (Fig. 1c). In addition, we tested susceptibility of both sRNA populations to capping enzyme and Terminator exonuclease. As shown in Fig. 1d, both sRNA populations are shifted one nucleotide higher by the capping enzyme treatment, indicating that these sRNAs have a 5′ di- or tri-phosphate structure. Additionally, both sRNAs are resistant to Terminator exonuclease treatment, which degrades 5′ mono-phosphate RNA. A pre-labeled radioactive 5′-mono-phosphate RNA (a spike-in control) is not shifted by capping enzyme but can be readily degraded by Terminator exonuclease. Taken together, both sRNA sequencing data and Northern blot analyses confirm that *E. histolytica* contains 27 nt as well as 31 nt sRNAs. Both sRNA populations have 5′-end polyP structure and the 31 nt sRNAs differ from the 27 nt sRNAs at 3′-end by non-templated 3 or 4 adenosines.

### Both small RNA populations (27 nt and 31 nt) are unchanged during development of *E. invadens*

*E. invadens* is a reptilian parasite that is used to study amebic development in vitro [31, 32]. Previously, we sequenced the 27 nt sRNA population from *E. invadens* parasites, and mapped these sRNAs to ~ 700 genes with low expression levels [22]. However, these genes with antisense sRNAs appear to be not developmentally regulated as sequencing of the 27 nt population at four developmental time-points showed identical sRNA targeted gene sets [22]. We first sought to check whether the 31 nt population was also present in *E. invadens.* Total RNA samples from trophozoites, 72 h encysted parasites, and parasites after 8 h excystation were radioactively labeled and separated on a denaturing 15% polyacrylamide gel. Two sRNA bands can be easily detected at 27 nt and 31 nt sizes (Suppl. Fig. 3A), indicating that *E. invadens* has both sRNA populations. To sequence the 31 nt sRNA population, we size-fractioned the 30-45 nt RNA and made sRNA libraries using the TAP method for all three samples. Similar to the observation in *E. histolytica*, the size distribution and mapping features of these libraries all showed 27 nt and 31 nt peaks, and 31 nt peak reads could not be directly mapped to the genome (Fig. 1e). Nucleotide compositions of the 31 nt population clearly show an oligo-A tail (Suppl. Fig. 3B). Genome mapping of these three libraries and mapping of their tail-clipped sequences are shown in Suppl. Table 2. Thus, we conclude that *E. invadens* also contains a sRNA population with non-templated A-tail*.* Using a similar approach as outlined previously [22], we analyzed the genes that mapped by sRNA from 31 nt populations among trophozoite, 72 h encystation, and 8 h excystation libraries. The overlap from these libraries is significant as shown in Suppl. Fig. 3C, indicating that the development process does not affect these genes, matching previous results with the sRNA from the 27 nt population. In summary, endogenous genes with antisense sRNAs seemed to be “locked” for silencing during development, which is reflected in both 27 nt and 31 nt populations.

### The relative abundance of two sRNA populations is linked to gene silencing efficacy

**Fig. 2:**
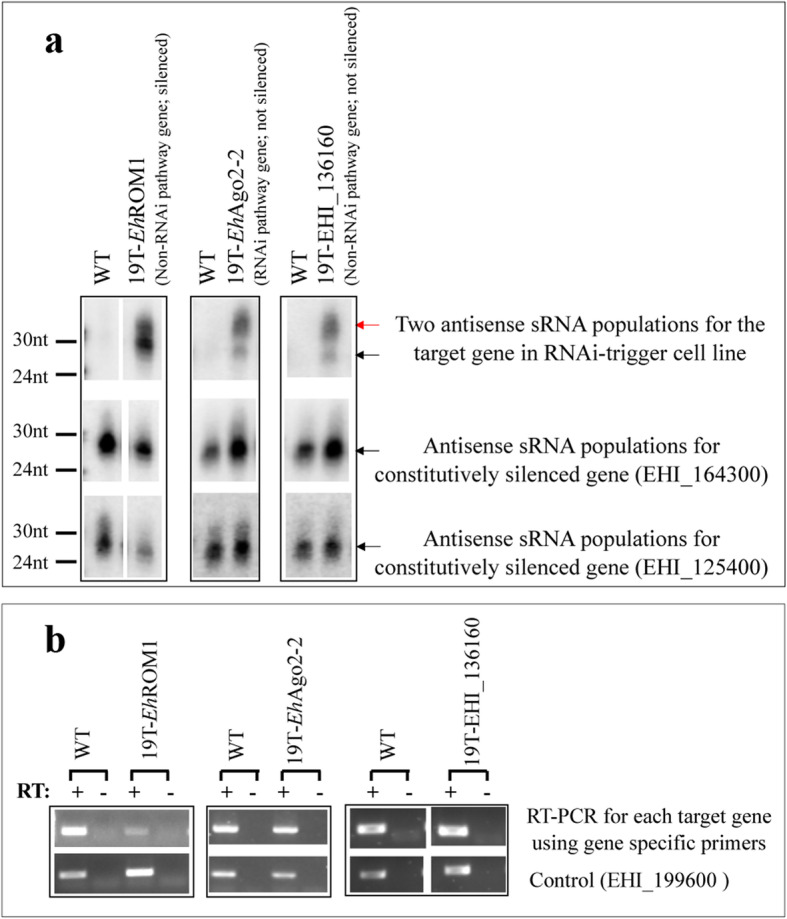
Northern blots detect relative abundance of two sRNA populations in RNAi-trigger cell lines. **a** Northern blot analysis detects antisense sRNA at both 27 nt and 31 nt sizes for each target gene. A gene specific sense probe was used for each target gene, and antisense signals are detected in the respective RNAi-trigger cell line. Relative abundance of sRNA populations: 27 nt > 31 nt in 19 T-*Eh*ROM1 cell line with *Eh*ROM1 gene silenced; in contrast, 27 nt < 31 nt in cell lines (19 T-*Eh*Ago2–2, 19 T-EHI_136160), where the target genes are not silenced. The ratio of the sRNA bands (31 nt/27 nt) measured by densitometry is 0.57 for 19 T-*Eh*ROM1; 6.7 for 19 T-*Eh*Ago2–2 and 7.0 for 19 T-EHI_136160. Antisense sRNAs to constitutively silenced genes EHI_164300 and EHI_125400 as controls, which further demonstrate the relative abundance pattern of 27 nt > 31 nt for the silenced genes. Red arrow points to 31 nt sRNA band, black arrow points to 27 nt sRNA band. See also Suppl. original blots for Fig. 2**a**. **b** Semi-quantitative RT-PCRs using gene specific primers. Gene expression levels of target gene were measured in RNAi-trigger cell lines: *Eh*ROM1 is silenced but the other two genes have equal expression in WT and RNAi-trigger cell lines. The fold change in gene expression compared to the control: 0.23 for 19 T-*Eh*ROM1; 1.45 for 19 T-*Eh*Ago2–2 and 0.95 for 19 T-EHI_136160. EHI_199600 is used as a loading control and -RT samples are shown. All RT-PCRs are specific, as a single band was generated for each primer set

We sought to explore the possible role of sRNA oligo-adenylation in the regulation of sRNA turnover in amoeba, a function that was ascribed to the di-adenylation of siRNAs in yeast [17]. We used cell lines that were transfected with RNAi-trigger plasmids. This approach was previously developed in our lab [33–35], and utilizes an episomal plasmid to overexpress a “trigger” sequence fused in-frame with a target gene. A 132 bp region of an endogenously silenced gene of EHI_197520 is used as trigger sequence, and is termed 19 T. Figure 2a shows that there are two bands corresponding to the size of 27 nt and 31 nt sRNA populations and can be detected for each target gene. We also observed that the relative abundance of two sRNA populations is indicative as to whether or not the target gene is silenced: for the cell line in which the *Eh*ROM1 gene (*E. histolytica* rhomboid protease 1, an intramembrane protease [36]) is silenced (Fig. 2b, the gene is downregulated by approximately 5-fold), there are much higher levels of the 27 nt population than the 31 nt population (Fig. 2a, lane labeled as 19 T-*Eh*ROM1; the ratio of the sRNA bands (31 nt/27 nt) measured by densitometry is 0.57). In contrast, the cell line in which the *Eh*Ago2–2 gene is not silenced (Fig. 2b, the change in gene expression is negligible, the fold change is 1.45), the 31 nt population is more abundant than the 27 nt population (Fig. 2a, lane labeled as 19 T-*Eh*Ago2–2; the ratio of the sRNA bands (31 nt/27 nt) is 6.7). In addition, we attempted to silence a gene that is not involved in RNAi (EHI_136160, a putative calreticulin precursor), and observed a similar phenomenon whereby the target gene was not silenced but a prominent 31 nt sRNA band was detected (Fig. 2a and b for lanes labeled as 19 T-EHI_136360; the ratio of the sRNA bands (31 nt/27 nt) is 7.0; there is no change in gene expression, the fold change is 0.95). Control sRNAs for constitutively silenced genes (EHI_164300 and EHI_125400) have signal that correspond mostly to the 27 nt population. Thus, the two sRNA populations can be detected in the cell lines transfected with RNAi-trigger plasmids, and their abundance level is linked to gene silencing efficacy.

### The three *Eh*Ago proteins all bind to 27 nt sRNAs

**Fig. 3:**
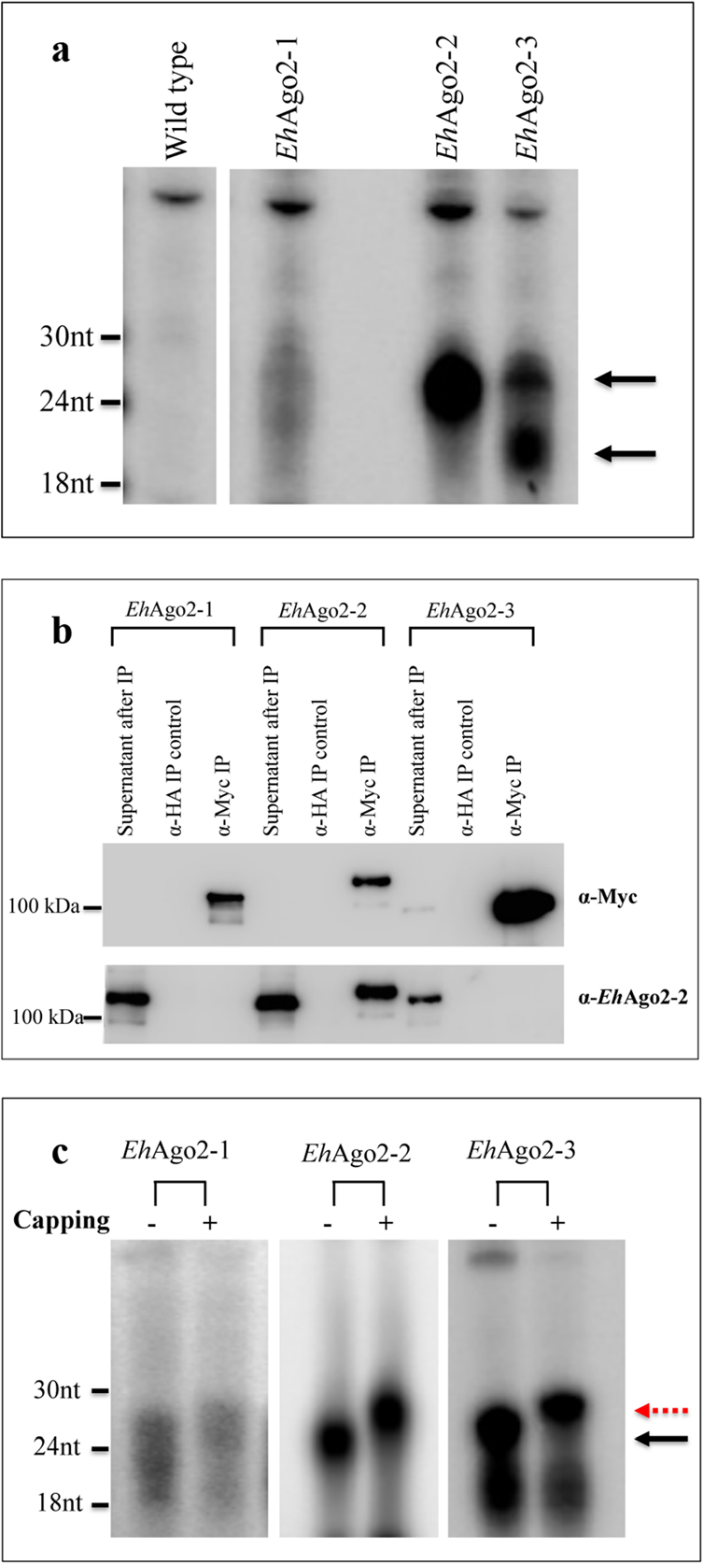
All three *Eh*Agos associate with 27 nt sRNAs with 5′-polyP structure. **a** sRNA populations are bound to all three *Eh*Ago proteins. Total RNA was prepared from anti-Myc IP using lysates from each Myc-tagged *Eh*Ago overexpressing cell line and labeled with α-[^32^P]-pCp. A faint band ranging from 20 to 30 nt was noted for *Eh*Ago2–1, a distinct 27 nt sRNA band identified for *Eh*Ago2–2, and two sRNA populations at 27 nt and 21 nt was observed for *Eh*Ago2–3. Arrows point to 27 nt and 21 nt sRNA bands. See also Suppl. original blots for Fig. 3**a**. **b** Western blot analysis detects a specific Myc signal for each *Eh*Ago IP. Anti-Myc IPs along with control (anti-HA IP) were performed for three Myc-tagged *Eh*Ago overexpressing cell lines. The Myc signal is detected at the expected size for each Myc-tagged *Eh*Ago using anti-Myc antibody, and is absent in the control IP. The same membrane was stripped and re-probed using an anti-*Eh*Ago2–2 antibody. The detected signal is only present in the *Eh*Ago2–2 IP but not in the *Eh*Ago2–1 IP nor *Eh*Ago2–3 IP, showing the specificity of anti-Myc IP experiment. See also Suppl. original blots for Fig. 3**b**. **c** Capping assay demonstrates the 5′-polyP structure for the 27 nt sRNA populations. An increase in the sRNA size is observed for all three *Eh*Ago sRNA populations, indicating that they have a 5′-polyP structure. The smaller sized RNAs below 24 nt size in *Eh*Ago2–1 and *Eh*Ago2–3 do not shift in size indicating that they do not have 5′-polyP structure. See also Suppl. original blots for Fig. 3**c**

We recently reported that three *E. histolytica* Ago proteins have distinct subcellular localizations and demonstrated that the PAZ domain of each *Eh*Ago controls sRNA binding [25]. To further characterize the sRNA populations that bind to each *Eh*Ago**,** we used Myc-tagged *Eh*Ago overexpression lines and performed anti-Myc IP to isolate RNAs associated with each Ago (Fig. 3a). For *Eh*Ago2–2, a distinct 27 nt sRNA population was noted, as has been previously published [23]. For *Eh*Ago2–1, the sRNAs were much less abundant and seen as a faint smear at the 20-30 nt range. For *Eh*Ago2–3, two sRNA populations around sizes 27 nt and 21 nt were observed.

We tested the specificity of the anti-Myc IP with additional controls. IP using control beads (anti-HA) showed no signal at the sRNA range when compared with anti-Myc IP for each *Eh*Ago (Suppl. Fig. 4A). We also used Western blot analysis to demonstrate that each *Eh*Ago has a specific Myc signal at the expected sizes which is absent in the control IP (Fig. 3b). The same membrane was stripped and probed using anti-*Eh*Ago2–2 antibody. This demonstrated that the *Eh*Ago2–2 was identified only in the *Eh*Ago2–2 IP but not in the IP of *Eh*Ago2–1 or *Eh*Ago2–3, indicating that each IP is specific without cross-contamination with *Eh*Ago2–2 (Fig. 3b). Of note, *Eh*Ago2–2 is the only protein that is abundant enough to be detected in wildtype cell lysates by Western blot analysis; the other two *Eh*Agos are expressed at low levels, which can only be detected in the overexpressing cell lines. Hence, we could not easily test the other two Ago proteins for cross-contamination [25]. Given that *Eh*Ago2–2 is the most abundant Ago protein in *Entamoeba* and has the most abundant population of associated sRNAs, the ability to exclude its potential co-IP in *Eh*Ago2–1 and *Eh*Ago2–3 was important. Finally, we demonstrated that the sRNA population bound to each Ago was not affected by various high salt concentrations used in the IP wash (Suppl. Fig. 4B), indicating that each *Eh*Ago binds strongly to the associated sRNA population. Based on these data, we concluded that the sRNA profile shown in Fig. 3a is specific to the given *Eh*Ago protein being studied.

### The sRNAs bound to the three *Eh*Ago proteins have 5′-polyP structure

We have previously shown that sRNAs bound to *Eh*Ago2–2 have a 5′-polyP structure [23], a feature similar to the 22G sRNA found in *C. elegans* and *Ascaris* [7, 24]. To determine if sRNAs bound to *Eh*Ago2–1 and *Eh*Ago2–3 also have a similar 5′-polyP structure, we performed an RNA capping assay [7, 23]. We show that 27 nt sRNAs associated with both *Eh*Ago2–1 and *Eh*Ago2–3 shifted in size by one nucleotide; however, the smaller size RNAs (18-24 nt) within the same sample were unchanged with the capping assay (Fig. 3c). Overall, the data indicate that 27 nt sRNAs that associate with *Eh*Ago2–1 and *Eh*Ago2–3 have a 5′-polyP structure, whereas the lower size sRNAs do not. In order to define the 5′-structure for the lower sized sRNAs, *Eh*Ago2–3 IP sRNA sample was labeled at the 5′-end using either T4 polynucleotide kinase (PNK) or calf intestinal phosphatase (CIP) plus T4 PNK (Suppl. Fig. 4C). The signal for PNK labeling can be seen for the lower sized band but not for the upper 27 nt band. However, as expected, both the upper and the lower bands can be seen by CIP + PNK labeling, indicating that the lower sized sRNAs likely have a 5′-OH structure. Thus, these sRNAs may arise from an RNA degradation process. Our capping assay and 5′-end labeling analysis indicated that the three *Eh*Agos are all loaded with 5′-polyP sRNAs.

### Characterization of sRNA populations bound to three *Eh*Ago proteins

For a better understanding of the sRNAs associated with all three *Eh*Agos, we performed high throughput sequencing of sRNA libraries generated from anti-Myc IP RNA samples. The sRNA sequencing libraries were made by a 5′-P independent cloning method using two separate enzymatic treatments (either TAP or RNA 5′-pyrophosphohydrolase (RppH), see Methods). A total of six sRNA libraries (three with each enzyme treatment) were constructed and their sequencing depth of total reads and unique reads is listed in Suppl. Table 1. Our IP sRNA libraries also included an important *Eh*Ago2–2 mutant (*Eh*Ago2–2^△NLS-DR^). This mutant protein did not alter sRNA binding but caused protein localization to change from the nucleus to the cytoplasm [25].

The overall mapping of sRNAs bound to the three *Eh*Ago proteins is listed in Table 2 (Ago IP libraries based on RppH method) and in Suppl. Table 3 (Ago IP libraries based on TAP method). The percentage of reads that map to tRNA and rRNA are at similar levels among the three *Eh*Ago libraries (0.5–1% for tRNA; 13–19% for rRNA). Of note, the mutant *Eh*Ago2–2^△NLS-DR^ IP library had fewer rRNA reads (3.7%) when compared with the wildtype *Eh*Ago2–2 IP library (13.3%).

**Table 2:** Genomic categories that are mapped by sRNA reads by *Eh*Ago IP libraries

	5′ P-independent cloning, α-Myc IP libraries			
Categories	Myc-***Eh***Ago2-1	Myc-***Eh***Ago2-2	Myc-***Eh***Ago2-3	Myc-***Eh***Ago2-2^**△NLS-DR**^
Total reads	1,944,425	2,298,495	1,705,678	1,498,720
Unique (unique/total reads)	419,279 (21.6%)	599,121 (26.1%)	241,012 (14.1%)	391,003 (26.1%)
tRNA^a,S^	4,407 (1.0%)	4,944 (0.5%)	1,919 (0.4%)	2,028 (0.5%)
rRNA^a,S^	80,844 (19.3%)	79,488 (13.3%)	47,678 (19.8%)	14,344 (3.7%)
*Eh*LINEs^a,AS^	41,713 (9.9%)	15,275 (2.5%)	16,306 (6.8%)	41,820 (10.7%)
Map to rest of genome^a^	230,334 (54.9%)	388,040 (64.8%)	139,897 (58.0%)	282,829 (72.3%)
Map to predicted ORFs^a,AS^	168,968 (40.3%)	284,402 (47.5%)	109,020 (45.2%)	213,451 (54.6%)
Not mapped to genome (%)	61,981 (14.8%)	111,374 (18.6%)	35,212 (14.6%)	49,982 (12.8%)

^a^ Number of unique reads divided by total unique reads

^S^ Most reads are in sense orientation

^AS^ Most reads are in antisense orientation

Our sequencing datasets for the three *Eh*Ago sRNA libraries have few reads that mapped to *Eh*SINEs and EREs, however there are substantial sRNA reads that mapped to *Eh*LINEs. Among the three *Eh*Ago proteins, *Eh*Ago2–2 had significantly lower amounts of *Eh*LINE-derived sRNAs (2.5% with *Eh*Ago2–2; 9.9% with *Eh*Ago2–1 and 6.8% with *Eh*Ago2–3) (Table 2). For the mutant *Eh*Ago2–2^△NLS-DR^, we observed a higher percentage of *Eh*LINEs reads compared to the wildtype *Eh*Ago2–2 (10.7% vs. 2.5%).

The largest category (40%) of reads that mapped to the genome belong to ORFs, indicating the second major source of endogenous sRNAs in *Entamoeba* are derived from gene coding regions. We categorized the genes to which the sRNAs mapped using both a cutoff (≥ 20 sRNAs map to a gene) and antisense/sense ratio (Antisense (ratio > 2), Mixed (ratio 0.5–2) and Sense (ratio < 2)). As seen in Table 3, the number of ORFs in the Antisense group is the largest among the three categories for all three *Eh*Ago proteins and they overlap by a set of 226 ORFs (additional file 1). Both the TAP and RppH IP libraries rendered very similar results in terms of sRNA mapped genes, indicating the two different sRNA treatments work equally well, and sequencing depth used in this study is sufficient to identify the core ORFs targeted by sRNAs. Our data for all three *Eh*Ago-bound sRNA libraries further demonstrated that genes with antisense sRNAs have very low expression levels and that the distribution of the antisense reads is biased to the 5′-end of genes (Suppl. Fig. 5A and B). Lastly, we used the sequences from *Eh*Ago IP libraries to determine if *E. histolytica* antisense sRNAs have a “phased” feature. For the secondary sRNAs, “phase” means that they are generated on an RNA precursor transcript in a phased pattern initiated at a specific nucleotide position, with sRNAs starting positions occurring at regular intervals. We checked the first 540 bp region of each ORF for the mapped sRNA reads under a 27 bp window starting from the initiator ATG. The resulting frequency for each position (1–27) was plotted (Suppl Fig. 6). We found no apparent phased register for antisense sRNAs in *Entamoeba*, indicating that these sRNAs are likely not from Dicer processing.

**Table 3:** Antisense sRNAs mapped genes overlap among three *Eh*Ago IP libraries (TAP and RppH) and size-fractionated total RNA libraries (TAP)

			Three categories of genes			
			Sense (AS/S < 0.5)	Mixed (AS/S 0.5-2)	Antisense (AS/S >2)	Antisense (overlap among three *Eh*Agos)
		Libraries				
**α-Myc IP library**	**RppH**	Myc-*Eh*Ago2-1	116	13	319	226
		Myc-*Eh*Ago2-2	178	60	297	
		Myc-*Eh*Ago2-3	81	36	255	
		Myc-*Eh*Ago2-2^△NLS-DR^	73	24	300	
	**TAP**	Myc-*Eh*Ago2-1	79	31	324	238
		Myc-*Eh*Ago2-2	48	25	298	
		Myc-*Eh*Ago2-3	28	19	256	
**Total RNA**	**TAP**	15-30nt size fraction	57	68	244	
		30-45nt size fraction^*^	72	52	242	
		30-45nt size fraction with 3' end trimming^#^	57	24	234	

^*^ most mapped reads are in 27nt peak

^#^ most mapped reads are in 31nt peak

On a genome-wide scale, we used the Cuffdiff algorithm [37] to check if there are intragenic regions to which sRNAs from the three *Eh*Ago IP libraries map differentially. This is an approach similar to our genome-wide RNA-Seq study for identifying loci with differential mapping of mRNAs [22, 38]. Pair-wise comparisons among the three *Eh*Ago libraries identified a small number of loci with differential mapping of Ago-associated sRNAs. For example, 64 significant differences out of 2225 loci with mapped sRNAs were identified in the comparison between *Eh*Ago2–1 and *Eh*Ago2–2, and 51 out of 1734 loci were identified in the comparison between *Eh*Ago2–3 and *Eh*Ago2–2. These results again indicate that sRNAs bound to each of the three *Eh*Agos have very similar targets throughout the genome.

### The sRNAs that bind *Eh*Ago proteins are 27 nt in size and have a 5′-G bias

**Fig. 4:**
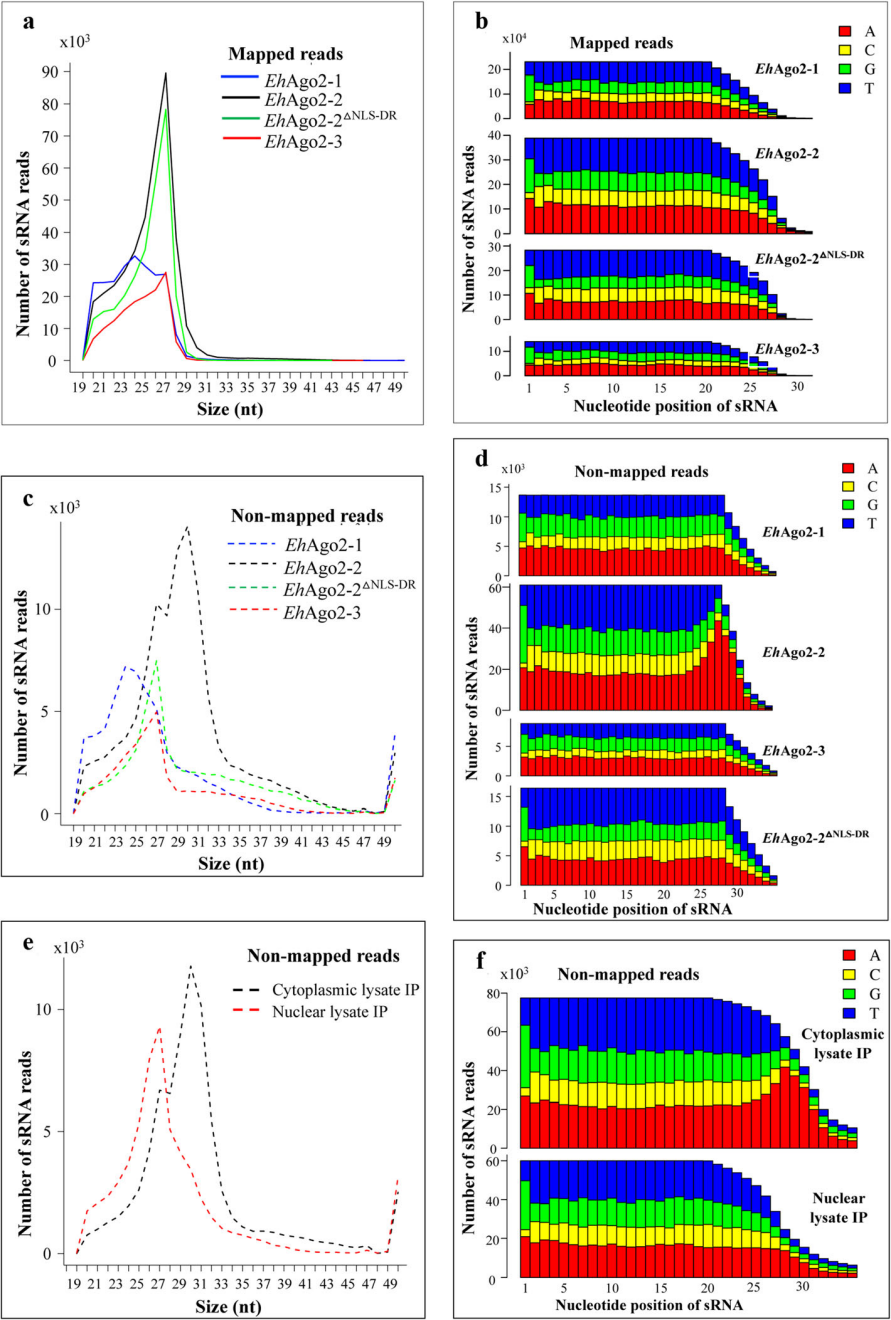
Characterization of sRNA populations bound to three *Eh*Agos. **a** Size distribution of the mapped reads (unique reads). Three *Eh*Ago IP libraries and *Eh*Ago2–2^△NLS-DR^ IP library were made by RppH method. An sRNA 27 nt peak is observed in all IP libraries. Both *Eh*Ago2–2 and *Eh*Ago2–2^△NLS-DR^ show a sharp 27 nt sRNA peak. *Eh*Ago2–1 and *Eh*Ago2–3 have a broad size distribution in addition to the 27 nt sRNA peak, which matches with the sRNA profile seen by pCp labeling. **b** Nucleotide distribution analysis for the mapped reads**.** 5**′**-G bias is evident for the first nucleotide in the mapped sRNA reads from all *Eh*Ago IP libraries. **c** Size distribution for the non-mapped reads. It shows a peak at 31 nt only in *Eh*Ago2–2 but not in the other two *Eh*Agos or *Eh*Ago2–2^△NLS-DR^. **d** Nucleotide distribution analysis for the non-mapped reads. Non-templated oligo-A tailing is present only in the *Eh*Ago2–2 library not in the others. **e** Size distribution analysis for the non-mapped reads in nuclear and cytoplasmic *Eh*Ago2–2 IP sRNA libraries. The 31 nt population is seen for cytoplasmic but not the nuclear *Eh*Ago2–2 IP sRNA library. **f** Nucleotide distribution analysis for the non-mapped reads show oligo-A tailing for cytoplasmic *Eh*Ago2–2 IP sRNA library

For the three *Eh*Ago-associated sRNA libraries, we determined the size distribution of sRNAs cloned from both the TAP and RppH methods and found that they are similar (Fig. 4a and Suppl Fig. 7A), indicating both enzymatic treatments made no difference in converting these 5′-polyP sRNAs for library cloning. The 27 nt sRNA peak can be seen in all *Eh*Ago libraries, with a sharp 27 nt peak for *Eh*Ago2–2 and *Eh*Ago2–2^△NLS-DR^. However, smaller size sRNAs are seen in *Eh*Ago2–1 and *Eh*Ago2–3 libraries by both TAP and RppH methods. This matches with the sRNA profile seen on the sRNA gel (Fig. 3a), where the lower sized RNAs have a 5′-OH structure and are likely a degradation product. In addition, we also checked the size distribution of the total reads (non-unique) for each library (Suppl. Fig. 8). There is a prominent peak at 27 nt and a very small peak at 21 nt in the *Eh*Ago2–3 IP library, indicating the smaller 21 nt RNA band was not cloned efficiently (as expected, likely because of their 5′-OH structure).

As the sRNAs bound to *Eh*Ago2–2 have a G bias in the 5′-nucleotide position [21, 22], we checked the nucleotide composition of sRNAs in the *Eh*Ago2–1 and *Eh*Ago2–3 IP libraries. We plotted nucleotide frequency at each position for each unique sRNA read and found the 5′-G bias again (Fig. 4b and Suppl. Fig. 7B).

For reads in the *Eh*Ago2–1 and *Eh*Ago2–3 libraries, there are significant sRNA reads in the size range of 18-27 nt. We tested to see if the 5′-G bias feature was true for the smaller size sequences in these libraries. We extracted subsets of 23-24 nt and 27 nt reads from the three *Eh*Ago libraries and compared their nucleotide composition (Suppl. Fig. 9). Both subsets show the 5′-G bias feature, indicating that 5′-end of sRNAs likely is intact between the two sampled 23-24 nt and 27 nt subsets. We did further mapping analysis of two subsets (23-24 nt reads against 27 nt reads) using Bowtie and found that the majority of reads in the 23-24 nt subset align perfectly to reads in the 27 nt subset (Suppl. Table 4). This indicates that the smaller reads are likely derived from 27 nt sRNA reads due to the degradation of 27 nt sRNAs at the 3′ end.

In order to determine if the actual sRNA species overlap among the three *Eh*Ago libraries, we performed Bowtie alignment analysis of the *Eh*Ago2–1 and *Eh*Ago2–3 libraries, using *Eh*Ago2–2 dataset as reference. We found that over 70% reads are aligned with reads in the *Eh*Ago2–2 library (Suppl. Table 5), indicating the identities of sRNA pool significantly overlap for the three *Eh*Agos. We also compared *Eh*Ago2–2^△NLS-DR^ with *Eh*Ago2–2, and the overlap for these two libraries is 75%, indicating that the mutation does not affect sRNA binding to this protein. We had previously noted that the *Eh*Ago2–2^△NLS-DR^ can efficiently bind sRNA similar to the wildtype protein [25], and the sequencing now confirms that the *Eh*Ago2–2^△NLS-DR^ mutant does not have an alteration in its associated 27 nt sRNA population.

As an added note, the sequencing libraries for TAP *Eh*Ago IP samples were made several years apart from the RppH *Eh*Ago IP samples. Analysis for both libraries rendered very similar results in terms of sRNA mapping features, size distribution profile, 5′-G bias, and antisense sRNA mapped genes. Based on these data, we concluded that all three *Eh*Agos bind to sRNA populations with significant overlap, mainly targeting retrotransposons and a core set of ~ 226 genes that are silenced in this organism.

### A small fraction of *Eh*Ago2–2-bound sRNAs are 31 nt sRNAs

Our sequencing data for the size-selected total RNAs showed that *E. histolytica* has a second sRNA population with a peak in the size of 31 nt, due to the non-templated additions of 3–4 adenosine(s) at the 3′-end of 27 nt sRNAs. In order to check if this 31 nt population can be associated to any specific *Eh*Ago, we analyzed non-mapped sRNA reads for each of the *Eh*Ago IP libraries. The size distribution of the non-mapped reads showed a 31 nt peak is only present in *Eh*Ago2–2 but not in the other two *Eh*Ago proteins or *Eh*Ago2–2^△NLS-DR^ (Fig. 4c). Additionally, the nucleotide distribution analysis for these non-mapped reads in *Eh*Ago2–2 showed 5**′**-G bias for the first nucleotide, and a string of 3 or 4 As was identified at the 3′-end (Fig. 4d). These results indicate that *Eh*Ago2–2 is the protein complex site involved in the oligo-adenylation of sRNA in the parasite.

Our previous work showed that cellular localization of *Eh*Ago2–2 is mostly in the nucleus with low levels of protein in the cytoplasm. However, the *Eh*Ago2–2^△NLS-DR^ mutant is excluded from the nucleus [25, 39]. In order to see if oligo-adenylated sRNAs had a different distribution between the cytoplasm and nucleus, we performed cell fractionation for nuclear and cytoplasmic lysates based on previously published methods [40] using both Myc-*Eh*Ago2–2 and Myc-*Eh*Ago2–2^△NLS-DR^ cell lines. The sRNAs isolated from anti-Myc IP samples are shown in Suppl. Fig. 10. As expected, the 27 nt sRNAs were significantly depleted in the nuclear fraction for *Eh*Ago2–2^△NLS-DR^ due to its protein localization to the cytoplasm. In contrast, the 27 nt sRNAs were found at almost equally high levels in both nuclear and cytoplasmic fractions for *Eh*Ago2–2. We sequenced the sRNA libraries made from nuclear and cytoplasmic IP RNA samples. Sequence alignment data are shown in Suppl. Table 6. Both libraries had similar percentages for every mapped genomic category, except that the cytoplasmic IP library had a larger percentage of non-mapped reads (19%) than the nuclear IP library (11.8%) (Suppl. Table 6). The non-mapped reads from both libraries were further analyzed for the size distribution. Figure 4e demonstrates that the 31 nt population is present in *Eh*Ago2–2 cytoplasmic IP but not in the *Eh*Ago2–2 nuclear IP. Figure 4f shows these 31 nt sRNAs have oligo-A tails at the 3′-end by nucleotide distribution analysis.

Our sequencing data for both total and Ago-bound RNAs show that *E. histolytica* has two sRNA populations, and the 31 nt sRNAs are formed from 27 nt sRNAs by oligo-adenylation at the 3′-end. We confirmed the presence of 27 nt and 31 nt sRNA populations for constitutively silenced genes, as well as for the genes that were targeted for the gene silencing using an episomal RNAi-trigger plasmid. All three *Eh*Agos associate with 27 nt sRNAs with mostly overlapping features. Only *Eh*Ago2–2 was found in partial association with 31 nt sRNAs, a process that appears to occur in the cytoplasm. Our finding of sRNA with oligo-adenylation modification is the first report of sRNA modifications among the pathogenic unicellular parasites which contain an RNAi pathway.

**Fig. 5:**
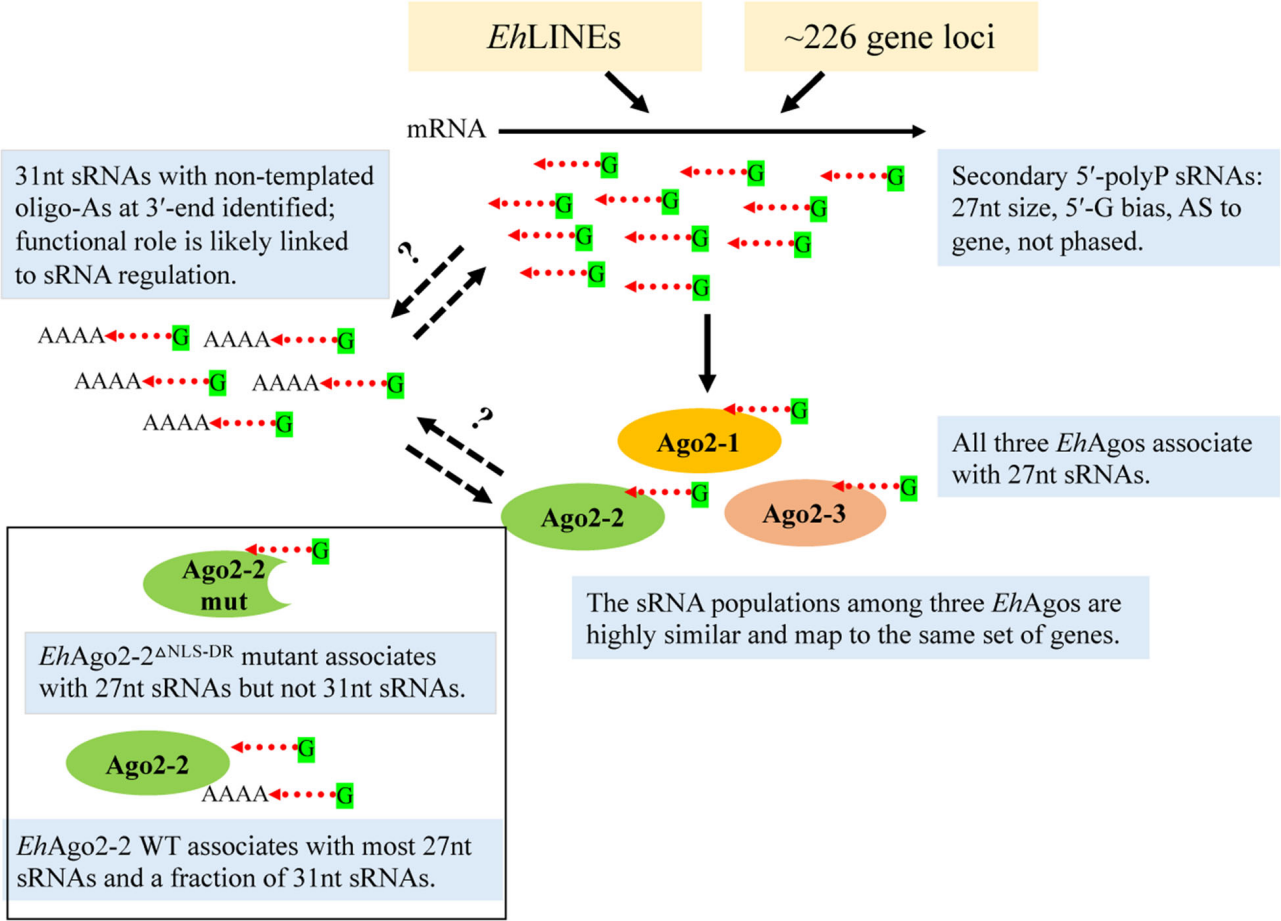
*E. histolytica* endogenous sRNA populations and their association with *Eh*Agos. *E. histolytica* has two endogenous sRNA populations: 27 nt and 31 nt sRNAs. Both largely align to either genomic *Eh*LINEs or a core set of 226 silenced gene loci. These sRNAs have features such as 5′-polyP structure, 5′-G bias, antisense to gene, and not-phased. The 31 nt sRNAs differ from 27 nt sRNAs at the 3′-end with addition of non-templated 3 or 4 As. All three *Eh*Agos associate to 27 nt sRNA populations and these sRNAs are largely overlapping. The 31 nt sRNAs are in partial association with *Eh*Ago2–2 but not with other two *Eh*Agos or mutant *Eh*Ago2–2^△NLS-DR^ (shown in the boxed area). The functional role of 31 nt sRNAs is linked to sRNA regulation, but the exact underlying mechanism for its biogenesis or function await further study. Red dotted arrows represent sRNAs (either with or without oligo-A tail). Solid black arrows indicate data supported by sRNA sequencing; dashed black two-way arrows with a question mark indicate possible routes for sRNA modification between two sRNA populations, or between 31 nt sRNAs and the *Eh*Ago2–2-loaded 27 nt sRNAs

Figure 5 summarizes the findings in this study. We see that *E. histolytica* has abundant 5′-polyP 27 nt sRNAs, with sRNA features consistent with RdRP products (they are 5′-G biased, mostly antisense, and are not phased). The 27 nt sRNAs are loaded into three *Eh*Ago proteins in a non-distinguishable manner. The endogenous sRNAs are mainly derived from *Eh*LINEs and a core set of ~ 226 silenced gene loci. We identified a second sRNA population at 31 nt which is due to the modification of 27 nt sRNAs at the 3′-end with 3 or 4 As; These 31 nt sRNAs are found in partial association with *Eh*Ago2–2 while the intact *Eh*Ago2–2 RISC is in cytoplasm. The functional roles corresponding these non-templated sRNAs await further study.

## Discussion

The RNAi pathway regulates gene expression through Ago proteins and their bound sRNAs. Here, we performed high throughput sRNA sequencing to uncover sRNA species in *E. histolytica*, focusing on sRNAs that are from size-fractionated total RNA and sRNAs that are associated with the three amebic *Eh*Ago proteins. Our sRNA sequencing data uncovered two sRNA populations in the parasite: 27 nt sRNA with 5′-polyP structure and a 5′-G bias and a second 31 nt sRNA population formed by oligo-adenylation of the 27 nt sRNAs at the 3′-end. We confirmed both populations by Northern blot analysis and showed that the abundance of the two sRNA populations is linked to gene silencing efficacy. The 31 nt sRNA populations are also present in the *E. invadens*; sequencing of the 31 nt population indicated that they are unchanged during development. While most sRNAs bound to *Eh*Ago2–2 are the 27 nt population, a fraction of sRNA reads belong to 31 nt sRNAs and can be recovered from a sRNA library generated from cytoplasmic but not nuclear fractions. Despite each *Eh*Ago protein having distinct cellular localization, sRNA sequencing data for the three *Eh*Agos demonstrate that they mostly overlap and sRNAs that bind to all three *Eh*Ago proteins largely target retrotransposons and a core set of ~ 226 genes marked for gene silencing.

The sRNA sequencing presented in this study provides a full account of the three *Eh*Ago-bound sRNAs in *E. histolytica.* We identified a similar pool of sRNAs among all three *Eh*Agos, indicating that the three *Eh*Agos are probably loaded from the same pool of secondary sRNAs (Fig. 5). Differential loading of sRNA populations into different Agos has been reported in several model systems [41, 42]. As demonstrated in *Drosophila* and *Arabidopsis*, miRNAs and siRNAs are sorted into specific Ago proteins [43]; the first nucleotide at the 5′-end of the sRNA is used as selective identity for AGO loading: AGO1 has a preference for 5′-uridine, whereas AGO2 and AGO4 prefer 5′-adenosine [44]. *C. elegans* has 27 Ago proteins; some of these Agos are specific to either primary or secondary sRNAs based on sRNA sizes and 5′-end structures [7, 45, 46]. However, worm-specific WAGOs are semi-redundantly loaded with secondary 5′-polyP 22G sRNAs [47]. The four human Ago proteins mostly bind an overlapping set of sRNAs [48, 49], although some differences in miRNA loading and distribution were recently reported using deep sequencing methods [50, 51]. Our sRNA sequencing results revealed a similar sRNA dataset for three *Eh*Ago proteins indicating a possible redundant function of these proteins for *E. histolytica* RNAi.

sRNAs are regulated through various modifications: uridylation of siRNAs has been reported across multiple organisms for its degradation role [9]; single adenylation of miRNA has been suggested for a stabilizing function [9, 10]. In yeast *Schizosaccharomyces pombe*, the addition of non-templated nucleotides (1–2 adenosines or uridines) to the 3′-end leads to sRNA elimination [17]. We observed that the relative abundance of two sRNA populations is indicative as to whether or not the target gene is silenced by an RNAi-trigger plasmid. There are more 27 nt sRNAs relative to 31 nt sRNAs when a target gene is silenced, and vice versa. This may suggest parasites have a way to convert 27 nt sRNAs into oligo-adenylated 31 nt sRNAs; once sRNAs are adenylated, it can leads to their degradation. An alternative possibility could be that the 31 nt sRNA populations might represent the “inactive” sRNAs (not Ago-bound) that could convert to the “active” 27 nt sRNAs (Ago-bound). Further studies to identify the biological conditions and genetic pathways that contribute to sRNA modification and regulation will be important to pursue.

## Conclusion

In conclusion, our data provide the first comprehensive dataset of the three *E. histolytica* Ago-bound sRNA populations. We identified a second population of 31 nt sRNAs that results from non-templated RNA-tailing of the 27 nt sRNA populations with the addition of 3–4 adenosines. The sRNA oligo-adenylation modification event is the first to be discovered among unicellular parasites. Future studies on the functional roles and identification of the protein complexes for oligo-adenylation will provide more insights on the biology of amebic sRNA regulation.

## Methods

### Parasite culture, plasmids and cell lines

The wildtype *E. histolytica* trophozoites (HM-1:IMSS) were grown axenically under standard conditions as described previously [23, 52]. Three *Eh*Ago wildtype cell lines for overexpressing N-terminal Myc-tagged protein were constructed as in [25]. Myc-*Eh*Ago2–2^△NLS-DR^ is a mutant cell line of *Eh*Ago2–2 that lacks NLS-DR-rich motif region as reported in [25]. The RNAi-trigger gene silencing plasmids (19 T-*Eh*ROM1 (EHI_197460) and 19 T-*Eh*Ago2–2 (EHI_125650)) were made in previous studies [34, 35]. We made new cell lines by transfection of these plasmids in this study. For construction of plasmid 19 T-EHI_136160, the full-length gene of EHI_136160 was amplified from genomic DNA and cloned into the RNAi-trigger gene silencing vector using SmaI and XhoI sites, as reported in [34]. All parasite cell lines were maintained at 6 μg/ml G418. *E. invadens* strain IP-1 was cultured in LYI-S-2 at 25 °C as previously described [38]. We followed previously published methods for the encystation and excystation [22].

### Cell lysis, cytoplasmic and nuclear enriched fractions and IP

The cell lysates were made using either WT parasite cells or transfected parasite cells. The basic lysis buffer contains 20 mM Tris-HCl (pH 7.5), 1 mM MgCl_2_, 10% (v/v) glycerol and 50 mM NaCl. The complete lysis buffer was made by adding basic lysis buffer with IGEPAL CA-630 (equivalent of NP-40) at 0.5% (v/v) plus 1 mM NaF, 1 mM DTT, 1 mM PMSF and 1X HALT EDTA free protease inhibitors (Thermo Scientific) and RNase inhibitor (1 unit/ml). The cells were lysed on ice for 15 min, and centrifuged at max speed using a bench top centrifuge at 4 °C for 30 min, and the supernatant was stored at − 80 °C.

Cytoplasmic and nuclear enriched fractions were isolated based on published methods [40, 53] with some changes: three T25 flasks of confluent parasite cells were collected, washed and resuspended in 3 ml of buffer A (10 mM Tris-HCl, pH 7.5, 3 mM MgCl_2_, 10 mM NaCl) with protease inhibitors (1 μM leupeptin, 1 μM E-64-d, and 1X HALT protease inhibitor mixture) and incubated on ice for 15 min. IGEPAL was added to a final concentration of 0.5% (v/v), mixed briefly, and centrifuged for 10 min at 2000×*g* at 4 °C. The supernatant was collected and adjusted with NaCl to 150 mM and stored at − 80 °C as the cytoplasmic fraction. For nuclear fraction, the pellet was then washed with 1000 μl of buffer A (without IGEPAL), followed by centrifugation for 10 min at 500×*g* and 4 °C. The pellet was resuspended in 500 μl of buffer C (20 mM Tris-HCl, pH 7.5, 150 mM KCl, 3 mM MgCl_2,_ 10% glycerol, and 0.5% IGEPAL) supplemented with protease inhibitors and passed through a 27G needle for 5 times to break the nuclei. The samples were centrifuged for 20 min at max speed at 4 °C. The supernatant was collected and stored at − 80 °C as the nuclear fraction.

The IP protocol is identical to [25], which is an adaptation from [54]. Both anti-Myc and anti-HA beads (Thermo Scientific) were used for IP experiments. Typically, 20 μl packed beads were used for each IP. A total of 250 μl crude lysate was further diluted with an equal volume of complete lysis buffer giving a protein concentration around 1 μg/μl. The IP mixture was rotated for 2 h at 4 °C. After binding, the beads were washed 6 times at 4 °C (5 min each) using a low stringency wash buffer (the basic lysis buffer plus 0.1% (v/v) Tween-20, 0.1% (v/v) NP-40, 1 mM PMSF and 0.5X HALT EDTA free protease inhibitors). In order to assess the stringency of the protein-sRNA interaction for certain experiments, the last three washes (5 min each) had varied salt concentrations with NaCl at low (50 mM), medium (250 mM), and high (500 mM). After the final wash step, the beads were pelleted at max speed for 1 min at 4 °C, and the beads were used for RNA preparation or eluted for protein Western blot.

### RNA isolation, pCp labeling and capping assay

For isolation of RNA from IP experiment, 300 ul of TRIzol (Invitrogen) reagent was added to the final IP beads, and total RNA was isolated using standard protocol. For total RNA/sRNA enriched RNA, we used the mirVana kit (Thermo Scientific) according to the manufacturer’s protocol. The procedures for the capping assay are similar as in [23]. The NEB Vaccinia capping system (New England Biolabs) was used for the capping assay. A reaction volume (20 μl) containing 2 μl of IP RNA or 10 μg sRNA-enriched RNA, 1X capping buffer, 1 μl vaccinia capping enzyme and 1 μl GTP (10 mM) and 1 μl SAM (2 mM). The reaction was incubated for 0.5 h at 37 °C. The capped RNAs were then extracted with acid phenol: chloroform. The radioactive labeling of 3′-end RNA was done by T4 RNA Ligase (New England Biolabs) using α-[^32^P]-pCp. For radioactive labeling of 5′-end RNA, we used KinaseMax kit (Thermo Scientific), either PNK or CIP + PNK reaction was set up using γ-[^32^P]-ATP following the manufacturer’s protocol. For the Terminator assay, total RNA (20 μg) was incubated with Terminator enzyme (30 °C for 1 h). A spike-in control (a pre-labeled radioactive 5′-monoP RNA) was included in the reaction as a substrate for Terminator Exonuclease. All RNA samples were resolved on a denaturing 15% polyacrylamide gel (7 M urea) and the radioactive signal was detected using a Phosphor screen and imaged on a Personal Molecular Imager (Bio-Rad).

### Northern blot analysis

Northern blot protocol was performed as in [23]. A sRNA-enriched RNA sample (20 μg or 50 μg) was separated on a denaturing 15% polyacrylamide gel and transferred to a membrane (Amersham™ Hybond™ -N+ Membrane, GE Healthcare). Probe DNA (Suppl. Table 7) was 5′-end labeled by PNK reaction using γ-[^32^P]-ATP. The [^32^P]-labeled DNA probe was then hybridized with membrane in perfectHyb buffer (Sigma) overnight at 37 °C. The membrane was washed using low (2X SSC, 0.1% SDS at 37 °C for 15 min) and medium (1X SSC, 0.1% SDS at 37 °C for 15 min) stringency conditions, and the radioactive signal was detected using a Phosphor screen and imaged on a Personal Molecular Imager (Bio-Rad). The ImageJ program was used for sRNA band quantification.

#### RT-PCR analysis

Total RNA was isolated from parasites using TRIzol reagent according to its protocol. The cDNA was synthesized from 1 μg total RNA using SuperScript™ IV VILO™ Master Mix kit with ezDNase™ Enzyme (Thermo Fisher Scientific) according to its suggested protocol. PCR conditions: one step denaturation: 94 °C for 30 s; 28 cycles: (94 °C for 15 s; 54 °C for 30 s; 72 °C for 60 s); final extension: 72 °C for 5 min. The primers used for RT-PCR can be found in Suppl. Table 7. The ImageJ program was used for PCR bands quantification.

### Size fractionation of total RNA

We used 60 μg sRNA-enriched RNA (from WT parasites) for the size fractionation experiments. RNA was loaded onto a denaturing 15% polyacrylamide gel, and separation of RNA was monitored by small RNA ladder. The RNA gel was stained with SYBR gold (Thermo Scientific) and visualized under UV light. The gel sections corresponding to the desired sRNA size range were cut out and minced into small parts for overnight extraction using buffer (20 mM Tris, pH 8.0, 1 mM EDTA, 0.3 M NH_4_OAc, 0.05% SDS). Extracted RNAs were then precipitated by isopropanol and resuspended in 20 μl water.

### Library construction and sequencing

For *Eh*Ago IP libraries, we combined three independent biological samples of anti-Myc IP RNAs for each *Eh*Ago line. For these samples, we constructed sequencing libraries using two separate enzymatic treatments: either TAP (Epicentre, this product was discontinued in 2014) or RppH (NEB). Both TAP and RppH can convert RNA of 5′-polyP into 5′-monoP. The pooled IP RNAs or size-fractionated RNAs were treated under suggested enzyme conditions at 37 °C for 20 min and extracted by acid phenol: chloroform. All RNA libraries were made using the NEBNext multiplex small RNA library prep set for Illumina (NEB#E7300S) following the manufacturer’s protocol. After cDNA generation, we incorporated barcode primers to each library by PCR (10–12 cycles). We quantified samples using Nanodrop and pooled for Illumina sequencing using the MiSeq platform.

The RppH IP libraries for three *Eh*Agos were sequenced more deeply than the TAP IP libraries, with depth of approximately 2 million reads among three *Eh*Agos, which generated approximately two times more unique reads than the TAP IP libraries. We therefore relied on the RppH sequencing libraries for the majority of the analysis presented in the paper. Our IP sRNA libraries included one important *Eh*Ago2–2 mutant (*Eh*Ago2–2^△NLS-DR^) and *Eh*Ago2–2 widetype nuclear and cytoplasmic IP samples, these samples were made using 5′-P independent cloning method based on RppH treatment. For all size-fractionated total RNA libraries, 5′-P independent cloning method (TAP) were used.

### Bioinformatics analysis

We used the same data processing pipelines as described previously [21, 22]. Raw reads were first processed to remove barcodes. We then mapped sequences for the unique reads (processed using Unix uniq command) to *E. histolytica* tRNA, rDNA, repetitive elements, transcripts and genome using Bowtie v1.2.2 (bowtie-bio.sourceforge.net) with the parameters: -v1 --all. Amebic sequences were obtained from amoebadb.org. The length profile and nucleotide distribution at each position were determined as previously reported [21] using the R package ShortRead [55]. To identify genes targeted by sRNAs, we used ≥ 20 sRNA reads as a cutoff as previously described [22]. For sRNA phasing analysis, we followed the concept used in [56]. Three *Eh*Ago IP libraries were used, the nucleotide position of the start of each antisense sRNA was obtained from Bowtie ORF alignment files. To simplify the question, we checked only the first 540 bp region of each ORF. The frequency of alignment of the first nucleotide of sRNAs to each position was calculated, and a custom R script was used to determine the count of reads at each position within a 27 bp window starting from the ATG. The resulting frequency for each position (1–27) was plotted.

## Supplementary Information

**Additional file 1. **Antisense sRNA mapped genes (226 genes) that overlap among three *Eh*Ago IP libraries. Listed are 226 genes with gene ID and gene annotation. Also listed are three datasheets containing Antisense group genes for each *Eh*Ago IP library: gene ID, antisense sRNA count, sense sRNA count, ratio of AS/S, gene expression in HM1.

**Additional file 2: Suppl. Figure 1.** Flow-chart of study design and bioinformatics analyses. For size fractionation libraries, total RNA from wildtype *E. histolytica* trophozoites or *E. invadens* three time points during development (trophozoite, 72 h encystation, and 8 h excystation) was size-fractioned, and cloned by 5′-P independent cloning method (TAP). For all *Eh*Ago anti-Myc IP libraries, 5′-P independent cloning (either TAP or RppH enzyme treatment) was used to identify sRNA populations associating with each Ago protein. All samples were made following NEB’s sRNA library construction protocol and sequenced by Illumina MiSeq platform. The raw reads were separated by barcodes, and sRNA sequences were processed. Bowtie alignments (−v1) were performed against tRNA, rRNA, retrotransposon elements, the genome and ORFs. **Suppl. Figure 2.** Analysis of genes mapped with sRNA and sRNA abundance level between two sRNA populations. **(A)** Number of unique sRNAs mapped to genes from both 27 nt and 31 nt populations are correlated. Counts in log10 scale for 27 nt population in x-axis, counts in log10 scale for 31 nt population in y-axis. **(B)** The abundance of individual sRNAs in each population is poorly correlated. Although two sRNA populations have significant overlap, the abundance of individual sRNAs cloned in each population differs greatly. The 27 nt sRNA populations are shown in x-axis, 31 nt sRNA populations in y-axis. Both axes are in log10 scale. **Suppl. Figure 3.** *E. invadens* 31 nt sRNA populations are not changed during development. **(A)** Both sRNA populations (27 nt and 31 nt) are present in *E. invadens* during development. Total RNAs from three time points during development (trophozoite, 72 h encystation, and 8 h excystation) were labeled with α-[^32^P]-pCp, and separated on a denaturing 15% polyacrylamide gel. Arrows show two sRNA bands at 27 nt and 31 nt. **(B)** Nucleotide distribution analysis of the non-mapped reads from *E. invadens* trophozoite dataset. There is a 5′-G bias for the first nucleotide, and a string of 3 or 4 As at 3**′**-end (all three time-point libraries had a similar pattern, only trophozoite dataset is shown as an example). **(C)** Venn diagram illustrates that genes mapped at all three time point libraries overlap greatly. After trimming/removal of non-templated oligo-As, sRNA reads can be remapped to the genome. Most genes overlap among all three time-point libraries, indicating that the 31 nt sRNA populations are un-changed during development. Note that the trophozoite dataset is under-sequenced compared to the other two datasets, hence there are fewer mapped genes and all are found in other datasets. **Suppl. Figure 4.** Verification of specificity of sRNA binding to each of three *Eh*Agos. **(A)** specific sRNA populations are observed in each *Eh*Ago anti-Myc IP. The IP experiments were performed under the same conditions for both sample and the control using iso-antigenic beads. Anti-HA IP was used as a control and generates no signal at sRNA range demonstrating the specificity of the anti-Myc IP for each *Eh*Ago. **(B)** The sRNA/Ago binding is unaffected by high salt concentration used in the IP wash. As shown, three NaCl concentrations for IP wash (low, medium, high) were used for anti-Myc IP experiments. Specific sRNA populations bound to each *Eh*Ago can be identified under all wash conditions. **(C)** 5′-end labeling tests for sRNA populations bound to *Eh*Ago2–3. The IP RNAs for both *Eh*Ago2–2 and *Eh*Ago2–3 were labeled at the 5′-end either by PNK using γ-[^32^P] ATP, or they were first treated with CIP, then labeled by PNK. For *Eh*Ago2–3, the lower band (20 nt) can be seen by PNK labeling, and also be seen to some degree by CIP + PNK. The upper band (27 nt) can only be seen by CIP + PNK labeling, indicating that the 27 nt sRNAs have 5′-polyP structure, and the lower band (20 nt) is mostly 5′-OH. The *Eh*Ago2–2 IP RNA is shown as a control, which has 27 nt sRNAs with 5′-polyP structure. **Suppl. Figure 5.** Antisense sRNA mapped genes among three *Eh*Ago IP libraries are silenced in *E. histolytica*. **(A)** Boxplot of normalized gene expression for three group genes identified in each of three *Eh*Ago IP libraries. Genes were divided into Antisense, Mixed and Sense groups based on the value of the AS/S sRNA ratio, see Table 3. Expression data were based on microarray data from *E. histolytica* trophozoites [57]. Gens in both Antisense and Mixed groups have very low expression levels. **(B)** sRNA distribution within the mapped genes using *Eh*Ago2–2 dataset. This analysis was based on the bowtie transcript alignment files. Unique reads that mapped to ORFs (Table 2, map to predicted ORFs) were assigned a position value based on the position of the starting nucleotide of the aligned sRNA read within each gene. The gene length for each ORF was normalized to one. Histograms for sRNA reads (y-axis) was plotted from 5′ to 3′-end according to their relative position within all genes for each group (x-axis). 5′-bias was noted for sRNAs that are mostly in antisense orientation (shaded as blue), 3′-bias was noted for sRNAs that are mostly in sense orientation (shaded as magenta). **Suppl. Figure 6.** Antisense sRNAs in three *Eh*Ago IP libraries show no phasing. For each of the three *Eh*Ago IP libraries, the nucleotide position of the start of each AS sRNA was obtained by Bowtie ORF alignment files. For the first 540 bp of each ORF, the frequency of alignment of the beginning of each sRNA to each position was calculated, and a custom R script was used to determine the count of reads at each position within a 27 bp window starting from the initiator ATG. The resulting frequency for each position (1–27) was plotted. **Suppl. Figure 7.** Size and nucleotide distribution of the mapped unique reads for *Eh*Ago IP libraries (TAP). **(A)** sRNA size distribution. Similar to *Eh*Ago IP libraries (RppH) in Fig. 4a, *Eh*Ago2–2 shows a sharp 27 nt sRNA peak, but *Eh*Ago2–1 and *Eh*Ago2–3 instead show a broad size distribution below its noticeable 27 nt sRNA peak. **(B)** Nucleotide distribution for sRNA reads. G bias is evident for the first nucleotide in mapped sRNA reads from all TAP IP libraries. Overall, both TAP and RppH IP libraries had similar results. **Suppl. Figure 8.** Size distribution of the raw reads for three *Eh*Ago IP libraries (RppH). After barcode removal, raw reads are checked for size distribution, which contains all redundant reads. For *Eh*Ago2–1, reads are broadly distributed at 20-30 nt range with noticeable peaks at 27 nt and 22 nt, which matches its profile seen in Fig. 3a. For *Eh*Ago2–2, a sharp peak at 27 nt matches its profile in Fig. 3a. For *Eh*Ago2–3, it has a sharp peak at 27n, and a much smaller peak at 22 nt. The obvious 20 nt band seen in Fig. 3 a was not efficiently picked up using our small RNA cloning method, further indicating these RNA species do not have 5′-polyP or 5′-monoP structure. **Suppl. Figure 9.** Comparison of two sampled subset reads: 23-24 nt subset versus the 27 nt subset in three *Eh*Ago IP libraries (RppH). Unique genome mapped reads in Table 2 were sorted into a 23-24 nt subset and 27 nt subset for each *Eh*Ago dataset. **(A)** The nucleotide distribution for each subset is plotted and show 5′-G bias feature is intact in 23-24 nt subset, indicating that smaller size reads are intact on 5′-end. **(B)** Bowtie mapping of 23-24 nt subset versus the 27 nt subset show most reads are aligned at position 1, indicating smaller subset reads are likely due to degradation of 27 nt sRNA on its 3**′**-end. **Suppl. Figure 10.** sRNA profile for anti-Myc IP RNAs using nuclear and cytoplasmic cell lysates. For *Eh*Ago2–2, the 27 nt sRNAs are almost equally presented between two cell fractions. *Eh*Ago2–2^△NLS-DR^ is used as a control, as it is localized to the cytoplasm, the IP for nuclear lysate brings down few sRNAs. **Suppl. Table 1.** Summary of all libraries used in the study. Listed are raw and unique reads generated for each library. For *Eh*Ago IP RNA libraries, 5′-P independent cloning method was based on either TAP or RppH enzyme treatment to convert 5′-polyP to 5′-monoP. For size-fractioned RNA libraries, 5′-P independent cloning method was based on TAP treatment. **Suppl. Table 2.** Genomic mapping for 31 nt sRNA population during *E. invadens* development. Raw and unique reads from *E. invadens* three time-points libraries during development (trophozoite, 72 h encystation, and 8 h excystation) were first mapped to genome (these are mostly 27 nt sRNAs carried over during gel separation), and non-mapped reads (mostly 31 nt sRNAs) were trimmed to 27nts and remapped to the genome. Listed are mapped unique reads and percentile for each category. **Suppl. Table 3.** Genomic categories that are mapped by sRNA for *Eh*Ago IP libraries (TAP). The Bowtie alignments (−v1) were used for mapping to tRNAs, rRNAs and repetitive elements, and genome including ORFs. Listed are mapped unique reads and the percentiles are calculated in parenthesis. **Suppl. Table 4.** Bowtie analysis of two sampled subset reads, 23-24 nt subset vs. 27 nt subset. For mapped unique reads (Table 2, Map to rest of genome), two subsets were generated based on the size criteria. Numbers of reads aligned between 23 and 24 nt subset vs. 27 nt subset are listed. Over 60% reads in 23–24 subset can be found in 27 nt subset, and most are aligned at position 1, indicating the smaller reads are likely degradation product from 3**′-**end of 27 nt sRNA. **Suppl. Table 5.** Bowtie analysis of reads among three *Eh*Ago IP libraries (RppH). We performed Bowtie alignment analysis of the unique reads in the *Eh*Ago2–1 and *Eh*Ago 2–3 libraries, using *Eh*Ago2–2 dataset as reference. Listed are overlap with their percentage. **Suppl. Table 6.** Genomic categories that are mapped by sRNA for nuclear and cytoplasmic *Eh*Ago2–2 IP libraries. The Bowtie alignments (−v1) were used for mapping to tRNAs, rRNAs and repetitive elements, and genome including ORFs. Listed are mapped unique reads and the percentiles are calculated in parenthesis. **Suppl. Table 7.** Oligo probes/primers used in this study. **Original blot for Fig.** 1**c and d** Original gel/blots used for Fig. 1c and d, refer to the figure legends in Fig. 1c and d. **Original blot for Fig.** 2**a** Original Northern blots used for Fig. 2A. ROM1 blots: three samples are loaded (WT, 19 T-LUC, 19 T-ROM1), both WT and 19 T-LUC are used as controls. The blot was first probed with ROM1 probe, which showed antisense sRNA signals of two populations with 27 nt > 31 nt. The blot was stripped and probed for two additional probes that are for endogenous sRNAs from genes EHI_ 164300 and EHI_ 125400, both showed two populations with 27 nt > 31 nt. For simplicity, only WT and 19 T-ROM1 were cropped and used in Fig. 2a. Ago2–2 blots: four samples are loaded (WT, 19 T-537 Ago2–2, 19 T-1064 Ago2–2, 19 T-FL Ago2–2). WT is used as control, three different length of Ago2–2 sequences (from 5′ ATG to the size indicated) were used as trigger. The blot was first probed with Ago2–2 probe, which showed antisense sRNA signals for all three trigger lines, two sRNA populations: 27 nt < 31 nt. The blot was stripped and probed for two additional probes that are for endogenous sRNAs from genes EHI_ 164300 and EHI_ 125400, both showed two populations with 27 nt > 31 nt. For simplicity reason, only WT and 19 T-FL Ago2–2 were cropped and used in Fig. 2a. EHI_136360 blot: three samples are loaded (WT, 19 T-EHI_136360, 19 T-EHI_136380). WT is used as control, two genes as listed were used as trigger. The blot was first probed with EHI_136360 probe, which showed specific antisense sRNA signals for two populations with 27 nt < 31 nt. The blot was stripped and probed for two additional probes that are for endogenous sRNAs from genes EHI_ 164300 and EHI_ 125400; both showed two populations with 27 nt > 31 nt. For simplicity, only WT and 19 T- EHI_136360 were cropped and used in Fig. 2a. **Original gel picture for Fig.** 2**b.** Semi-quantitative RT-PCRs using gene specific primers monitor the gene expression level of target gene in RNAi-trigger cell lines and control PCR for EHI_199600 was used as a loading control. For 19 T-EHI_136360, a second control line (19 T-luc) was included. Original blot for Fig. 3. (3A) Original blot, refer to the figure legends in Fig. 3a; (3B) Original Western blots of α-Ago2–2 and α-Myc, refer to the figure legends in Fig. 3b. (3C) Original blot for capping assay. Shown are two capping assay experiments for *Eh*Ago2–1 and *Eh*Ago2–3. Left panel contains IP RNAs from WT, *Eh*Ago2–1, *Eh*Ago2–3 under capping assay conditions. Both *Eh*Ago2–1 and *Eh*Ago2–3 samples showed upward migration of 27 nt sRNAs but not for the control. The 2nd experiment for *Eh*Ago2–3 sample was repeated and shown in Right panel, with clear upward migration of 27 nt sRNAs. Also refer to the figure legends in Fig. 3c. Capping assay for IP RNAs *Eh*Ago2–2 were done on a separate gel as shown. For simplicity, *Eh*Ago2–1, *Eh*Ago2–2, *Eh*Ago2–3 were cropped and used in Fig. 3c.

## Data Availability

The datasets of sRNA libraries generated and analyzed in the current study are available from the corresponding author on reasonable request. We also deposited all datasets to NCBI GEO (accession GSE157756).

## Abbreviations

RNAi: RNA interference
sRNA: Small RNA
Ago: Argonaute
dsRNA: Double-stranded RNA
siRNA: Short interfering RNA
RISC: RNA induced silencing complex
PTGS/TGS: Posttranscriptional / transcriptional gene silencing
RdRPs: RNA-dependent RNA polymerases
miRNAs: MicroRNAs
piRNAs: Piwi-interacting RNAs
TAP: Tobacco acid pyrophosphatase
RppH: RNA 5′-pyrophosphohydrolase
5′-polyP: 5′-polyphosphate
PNK: Polynucleotide kinase
CIP: Calf intestinal phosphatase
LINEs: Long interspersed nuclear elements
SINEs: Short interspersed nuclear elements
EREs: *Entamoeba* Repeat Elements
ORF: Open reading frame
WT: Wild type
