# Supplementary material for: Identification of oligo-adenylated small RNAs in the parasite Entamoeba and a potential role for small RNA control
Source: BMC Genomics. 2020 Dec 9;21:879. doi: 10.1186/s12864-020-07275-6 (PMC7724847; doi:10.1186/s12864-020-07275-6)
Supplement: Supplementary file 2 — Additional file 2: Suppl. Figure 1. Flow-chart of study design and bioinformatics analyses. For size fractionation libraries, total RNA from wildtype E. histolytica trophozoites or E. invadens three time points during development (trophozoite, 72 h encystation, and 8 h excystation) was size-fractioned, and cloned by 5′-P independent cloning method (TAP). For all EhAgo anti-Myc IP libraries, 5′-P independent cloning (either TAP or RppH enzyme treatment) was used to identify sRNA populations associating with each Ago protein. All samples were made following NEB’s sRNA library construction protocol and sequenced by Illumina MiSeq platform. The raw reads were separated by barcodes, and sRNA sequences were processed. Bowtie alignments (−v1) were performed against tRNA, rRNA, retrotransposon elements, the genome and ORFs. Suppl. Figure 2. Analysis of genes mapped with sRNA and sRNA abundance level between two sRNA populations. (A) Number of unique sRNAs mapped to genes from both 27 nt and 31 nt populations are correlated. Counts in log10 scale for 27 nt population in x-axis, counts in log10 scale for 31 nt population in y-axis. (B) The abundance of individual sRNAs in each population is poorly correlated. Although two sRNA populations have significant overlap, the abundance of individual sRNAs cloned in each population differs greatly. The 27 nt sRNA populations are shown in x-axis, 31 nt sRNA populations in y-axis. Both axes are in log10 scale. Suppl. Figure 3. E. invadens 31 nt sRNA populations are not changed during development. (A) Both sRNA populations (27 nt and 31 nt) are present in E. invadens during development. Total RNAs from three time points during development (trophozoite, 72 h encystation, and 8 h excystation) were labeled with α-[32P]-pCp, and separated on a denaturing 15% polyacrylamide gel. Arrows show two sRNA bands at 27 nt and 31 nt. (B) Nucleotide distribution analysis of the non-mapped reads from E. invadens trophozoite dataset. There is a 5′-G bias [file 12864_2020_7275_MOESM2_ESM.pptx]

## Slide 1
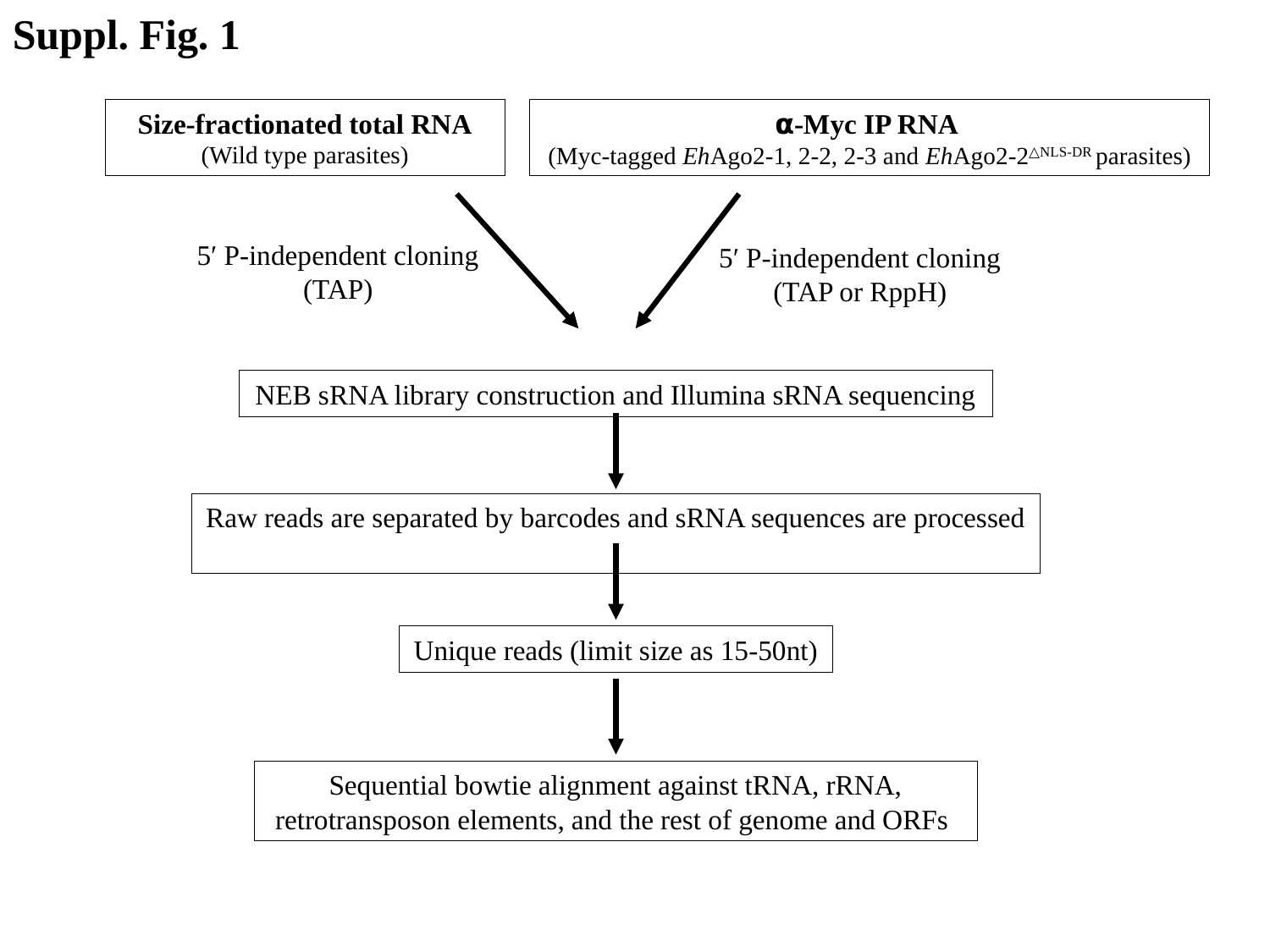

Suppl. Fig. 1
Size-fractionated total RNA
(Wild type parasites)
⍺-Myc IP RNA
(Myc-tagged EhAgo2-1, 2-2, 2-3 and EhAgo2-2△NLS-DR parasites)
5′ P-independent cloning
(TAP)
5′ P-independent cloning
(TAP or RppH)
NEB sRNA library construction and Illumina sRNA sequencing
Raw reads are separated by barcodes and sRNA sequences are processed
Unique reads (limit size as 15-50nt)
Sequential bowtie alignment against tRNA, rRNA, retrotransposon elements, and the rest of genome and ORFs

## Slide 2
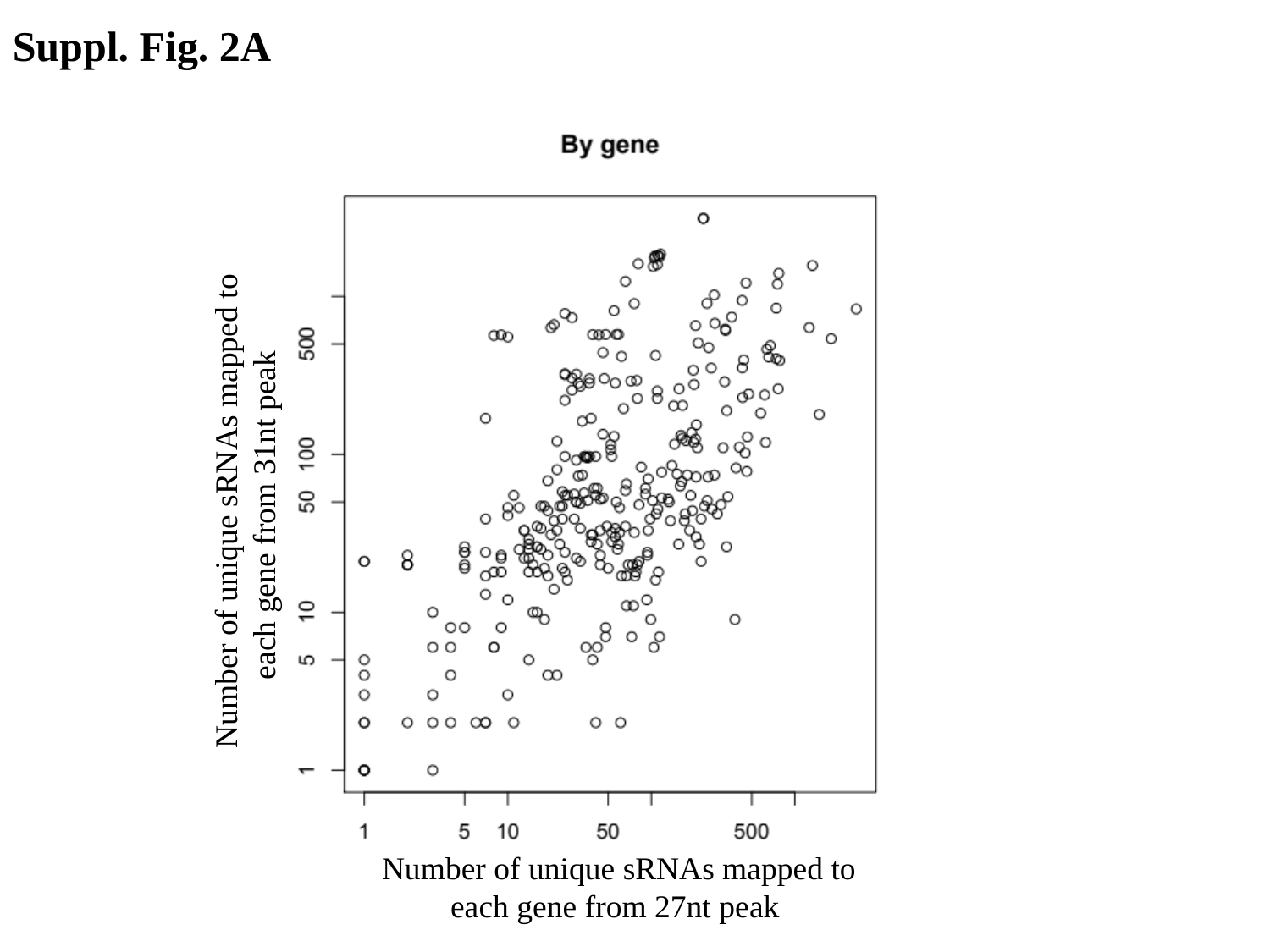

Suppl. Fig. 2A
Number of unique sRNAs mapped to each gene from 31nt peak
Number of unique sRNAs mapped to each gene from 27nt peak

## Slide 3
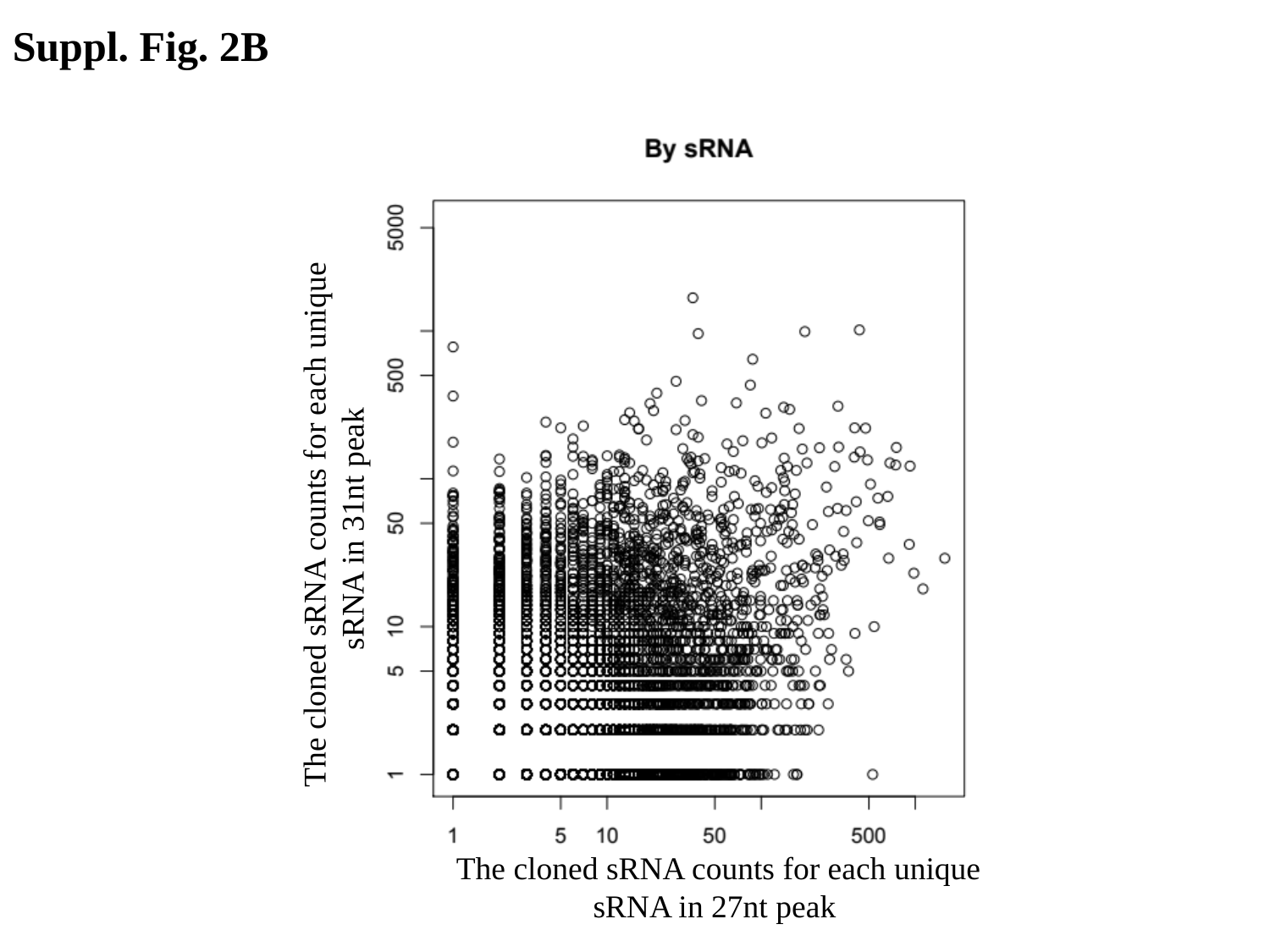

Suppl. Fig. 2B
The cloned sRNA counts for each unique sRNA in 31nt peak
The cloned sRNA counts for each unique sRNA in 27nt peak

## Slide 4
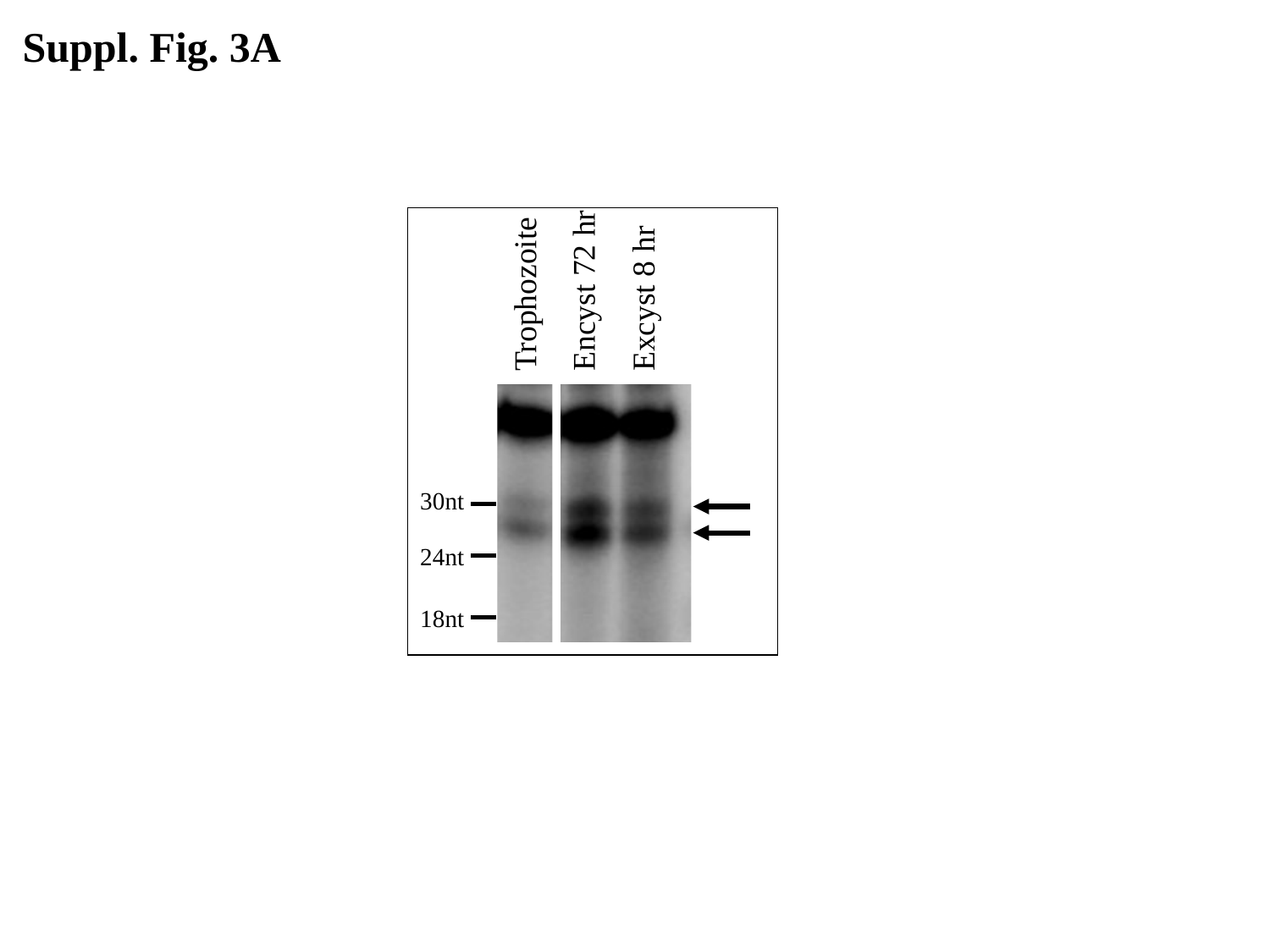

Suppl. Fig. 3A
Encyst 72 hr
Trophozoite
Excyst 8 hr
30nt
24nt
18nt

## Slide 5
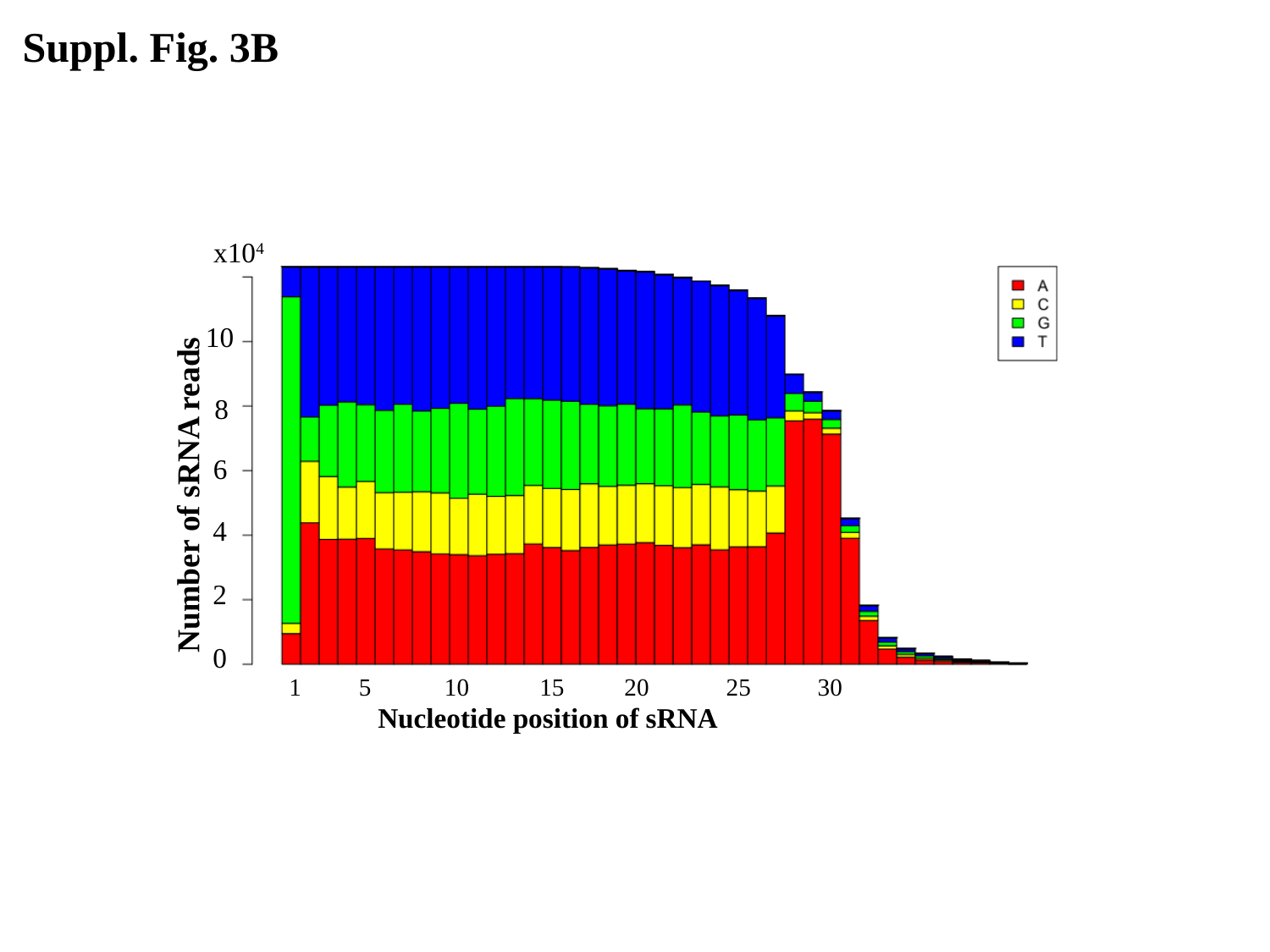

Suppl. Fig. 3B
x104
10
8
Number of sRNA reads
6
4
2
0
1
5
10
15
20
25
30
Nucleotide position of sRNA

## Slide 6
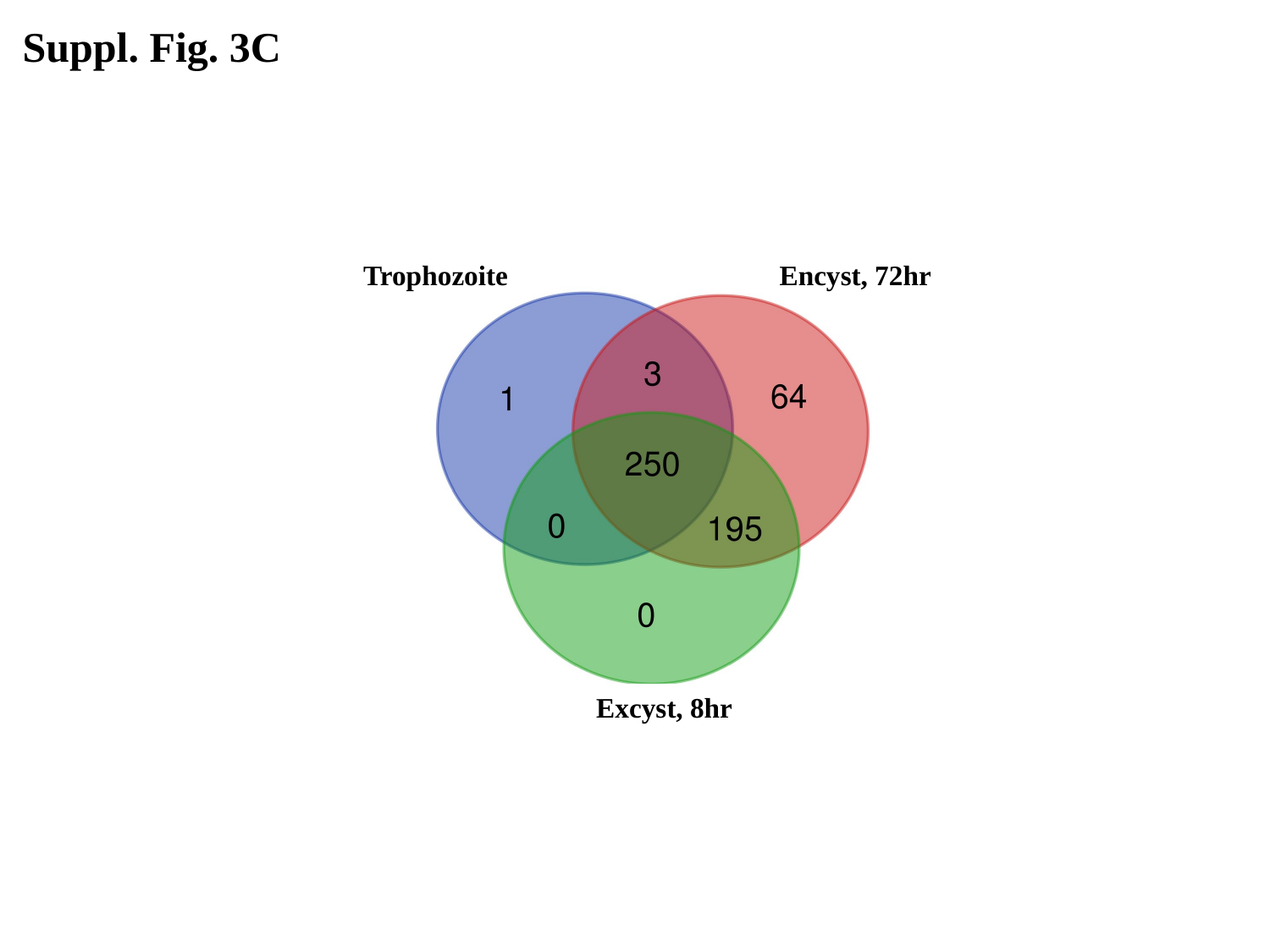

Suppl. Fig. 3C
Trophozoite
Encyst, 72hr
Excyst, 8hr

## Slide 7
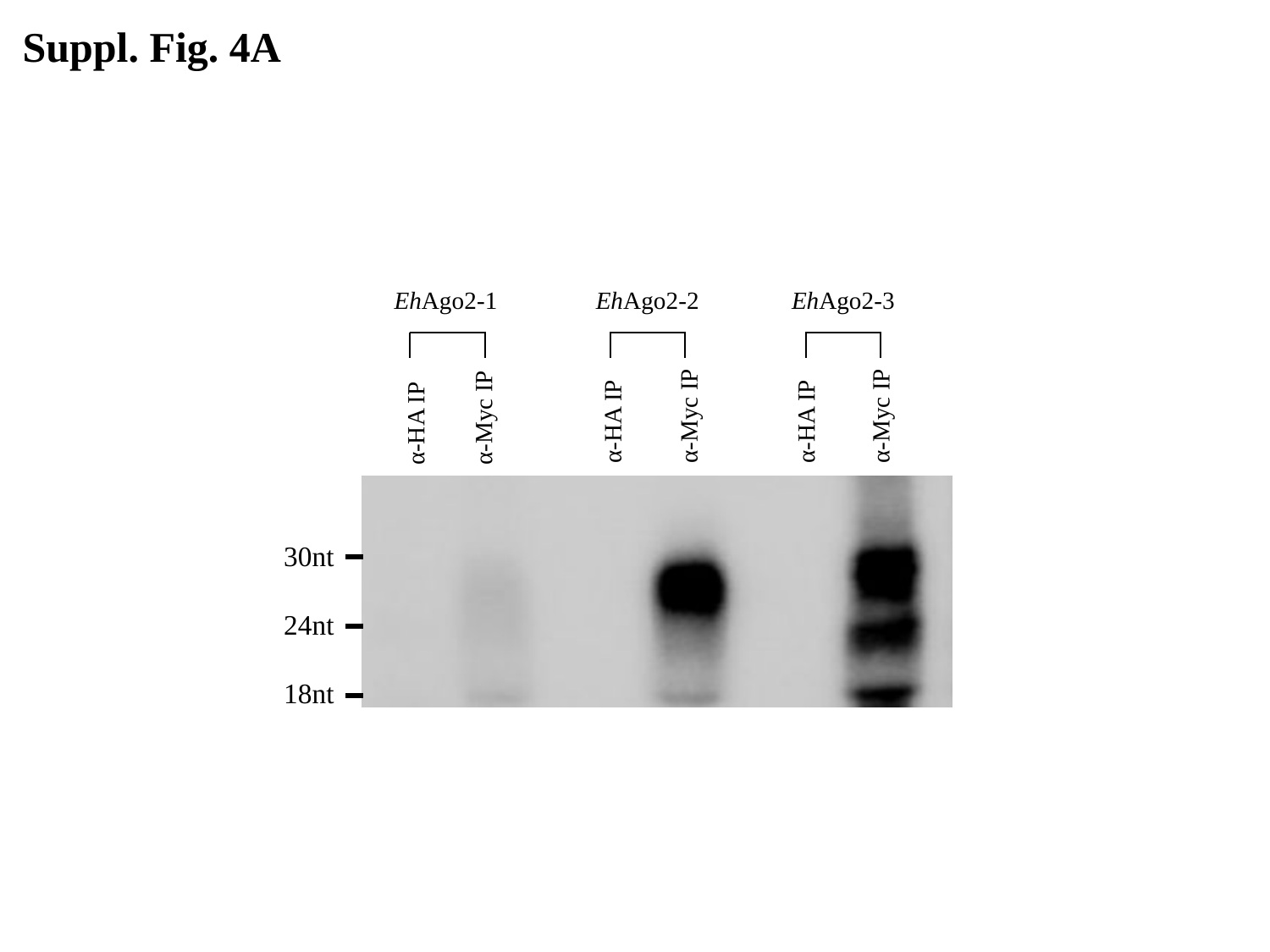

Suppl. Fig. 4A
EhAgo2-1
EhAgo2-2
EhAgo2-3
α-HA IP
α-Myc IP
α-HA IP
α-Myc IP
α-HA IP
α-Myc IP
30nt
24nt
18nt

## Slide 8
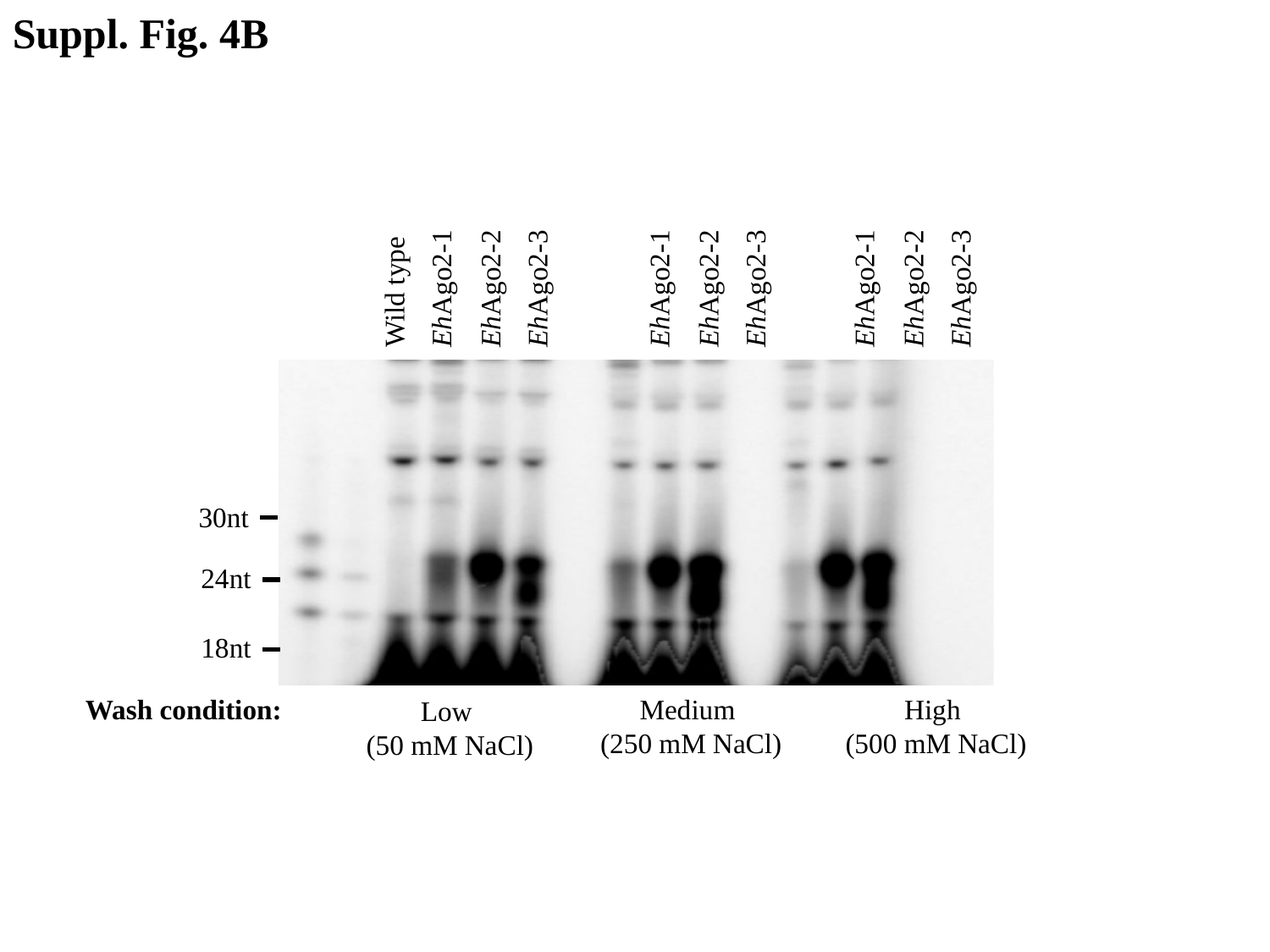

Suppl. Fig. 4B
EhAgo2-1
EhAgo2-3
EhAgo2-1
EhAgo2-3
EhAgo2-2
EhAgo2-2
EhAgo2-1
EhAgo2-3
EhAgo2-2
Wild type
30nt
24nt
18nt
Wash condition:
Medium
(250 mM NaCl)
High
(500 mM NaCl)
Low
(50 mM NaCl)

## Slide 9
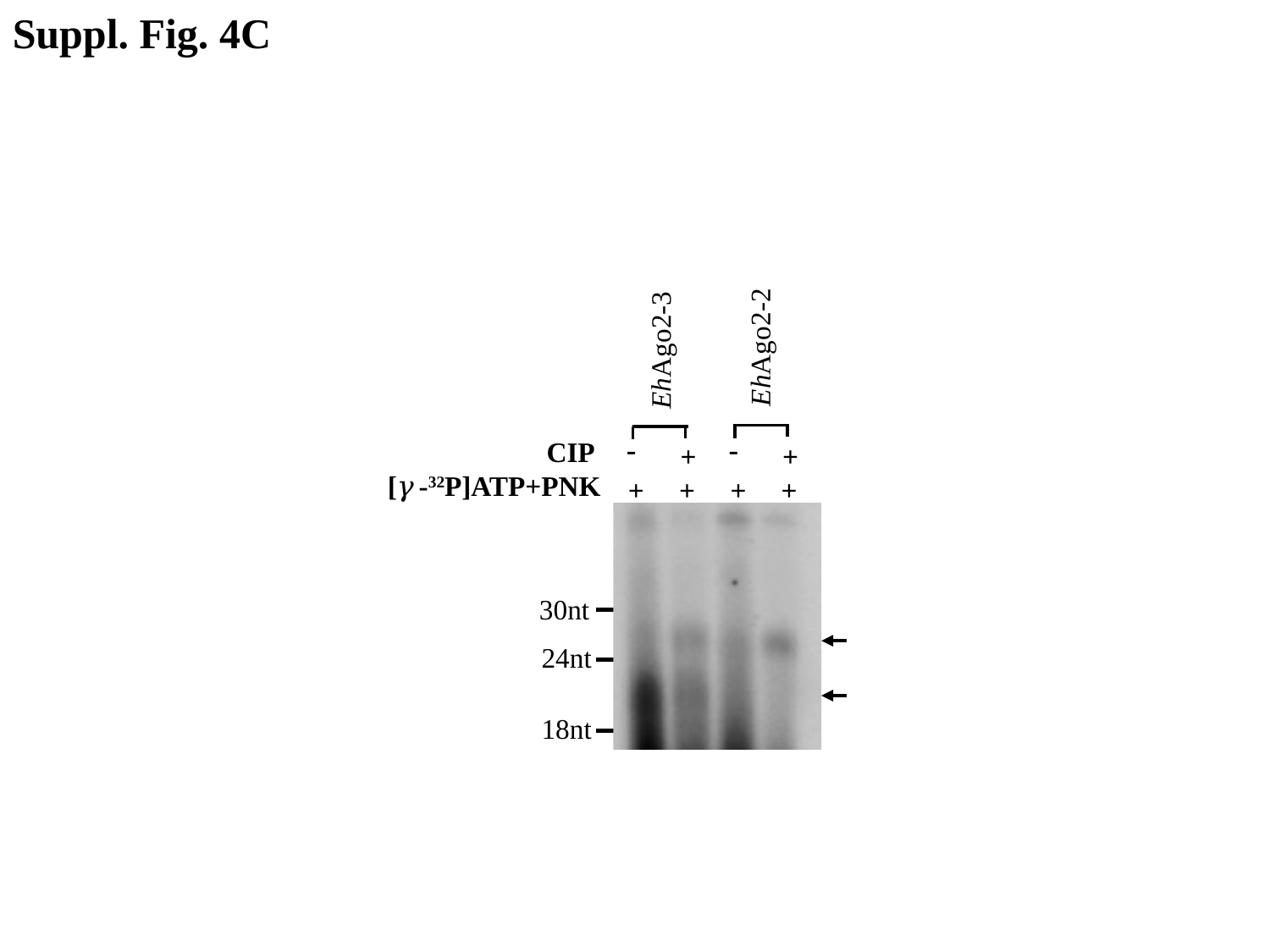

Suppl. Fig. 4C
EhAgo2-2
EhAgo2-3
1D
CIP
[𝛾-32P]ATP+PNK
 +
+ +
 +
+ +
30nt
24nt
18nt

## Slide 10
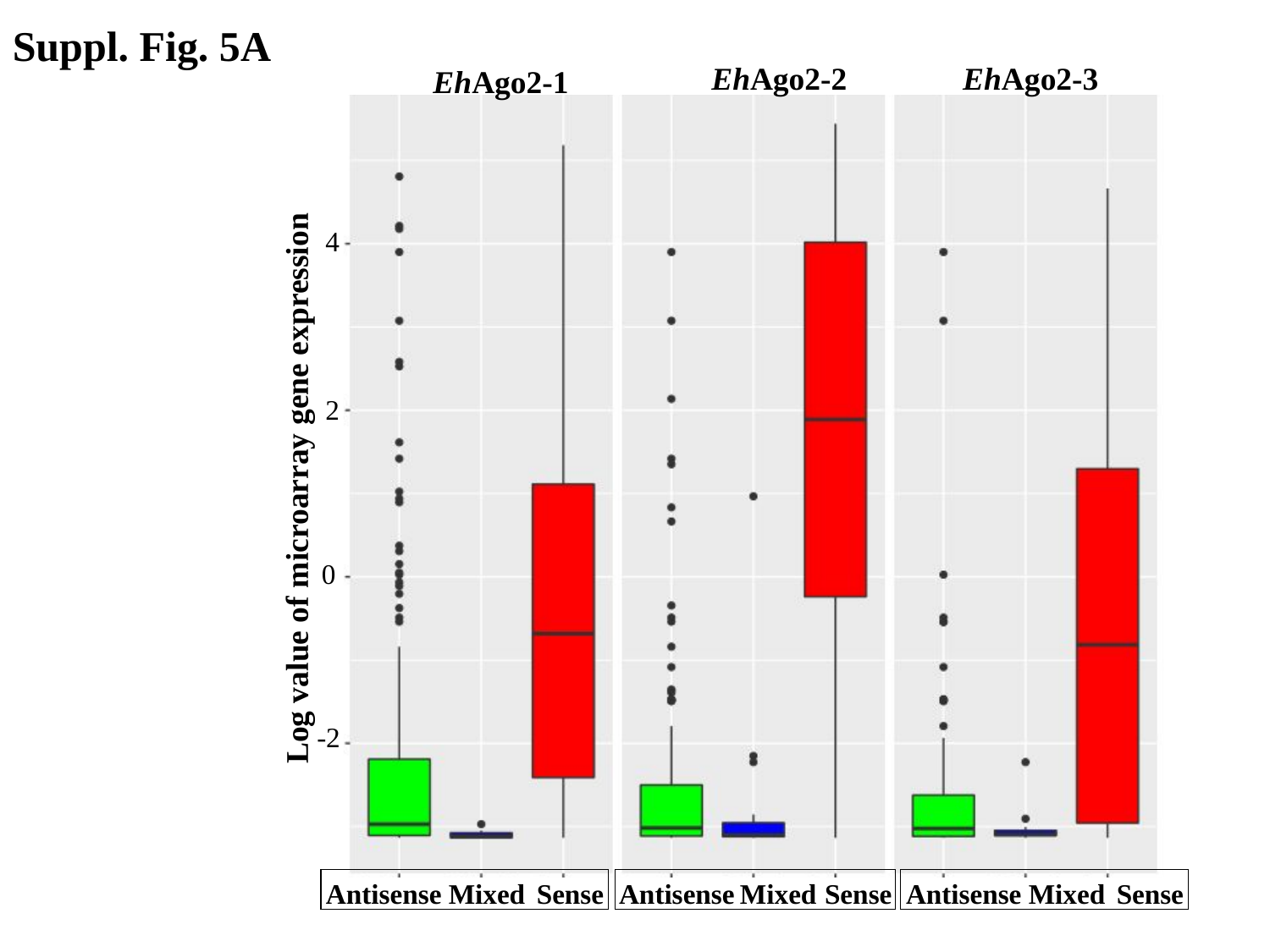

Suppl. Fig. 5A
EhAgo2-3
EhAgo2-2
EhAgo2-1
4
2
Log value of microarray gene expression
0
-2
Antisense
Mixed
Sense
Antisense
Mixed
Sense
Antisense
Mixed
Sense

## Slide 11
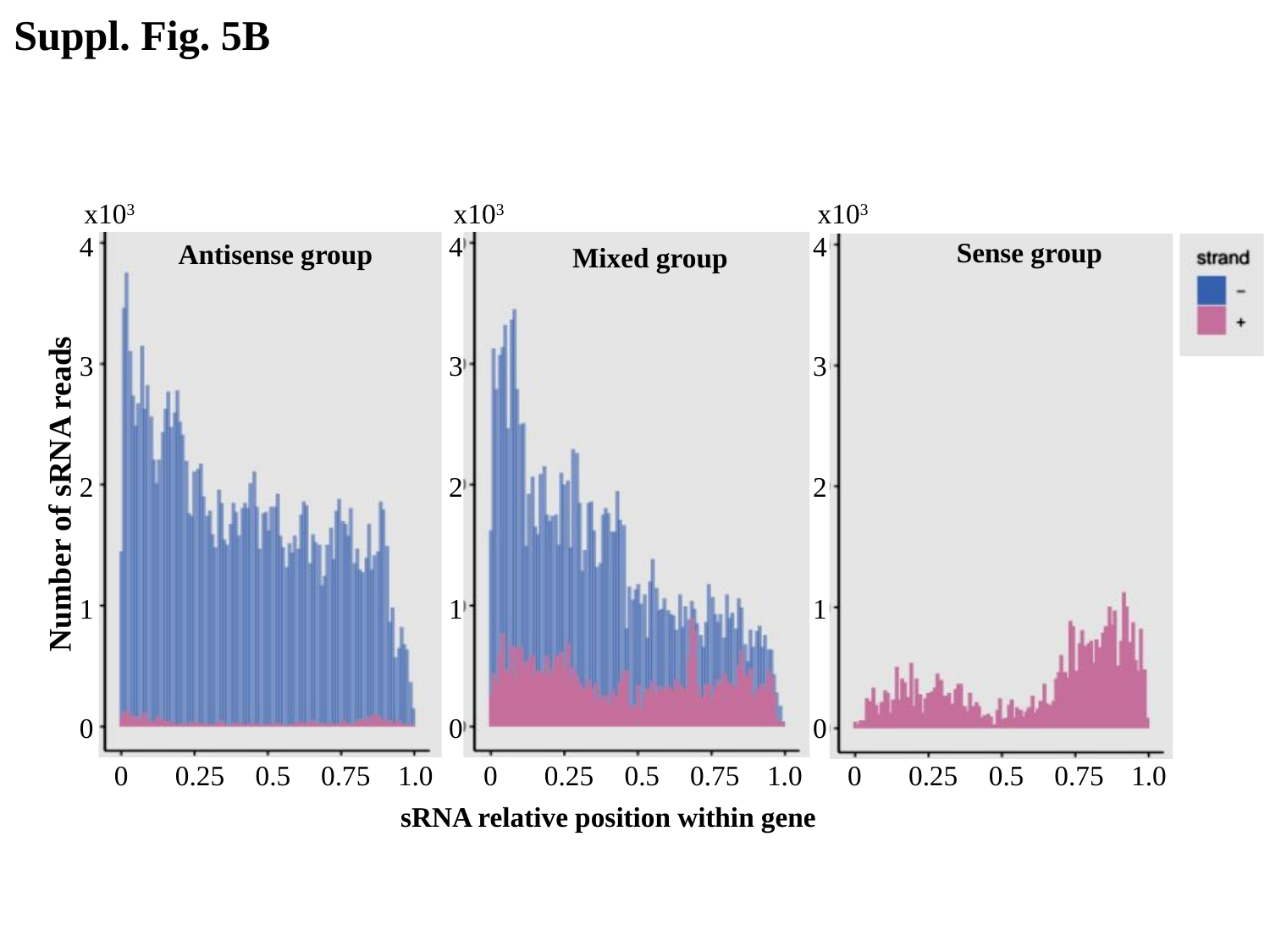

Suppl. Fig. 5B
x103
x103
x103
4
4
4
Sense group
Antisense group
Mixed group
3
3
3
Number of sRNA reads
2
2
2
1
1
1
0
0
0
0
0.25
0.5
0.75
1.0
0
0.25
0.5
0.75
1.0
0
0.25
0.5
0.75
1.0
sRNA relative position within gene

## Slide 12
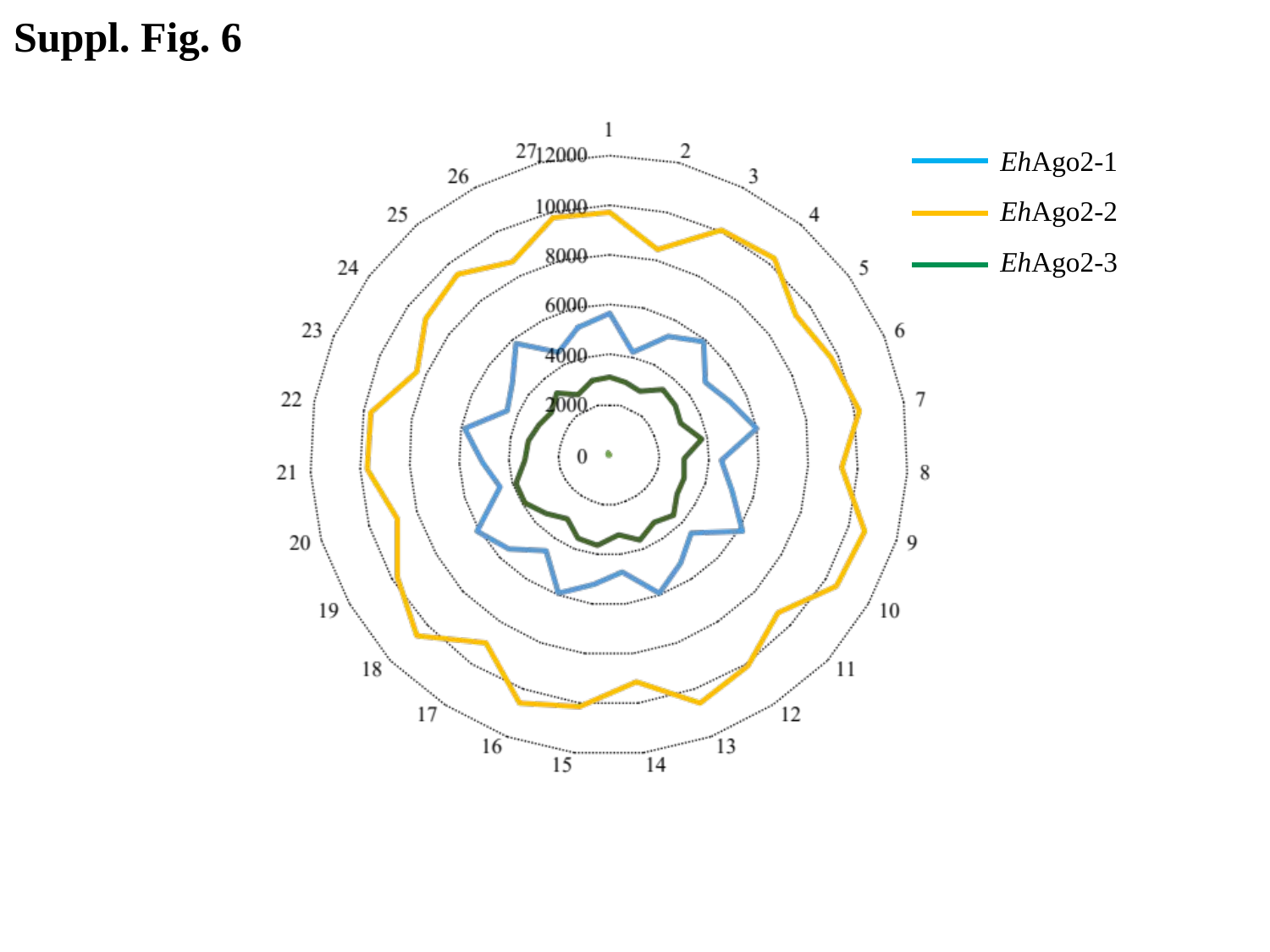

Suppl. Fig. 6
EhAgo2-1
EhAgo2-2
EhAgo2-3

## Slide 13
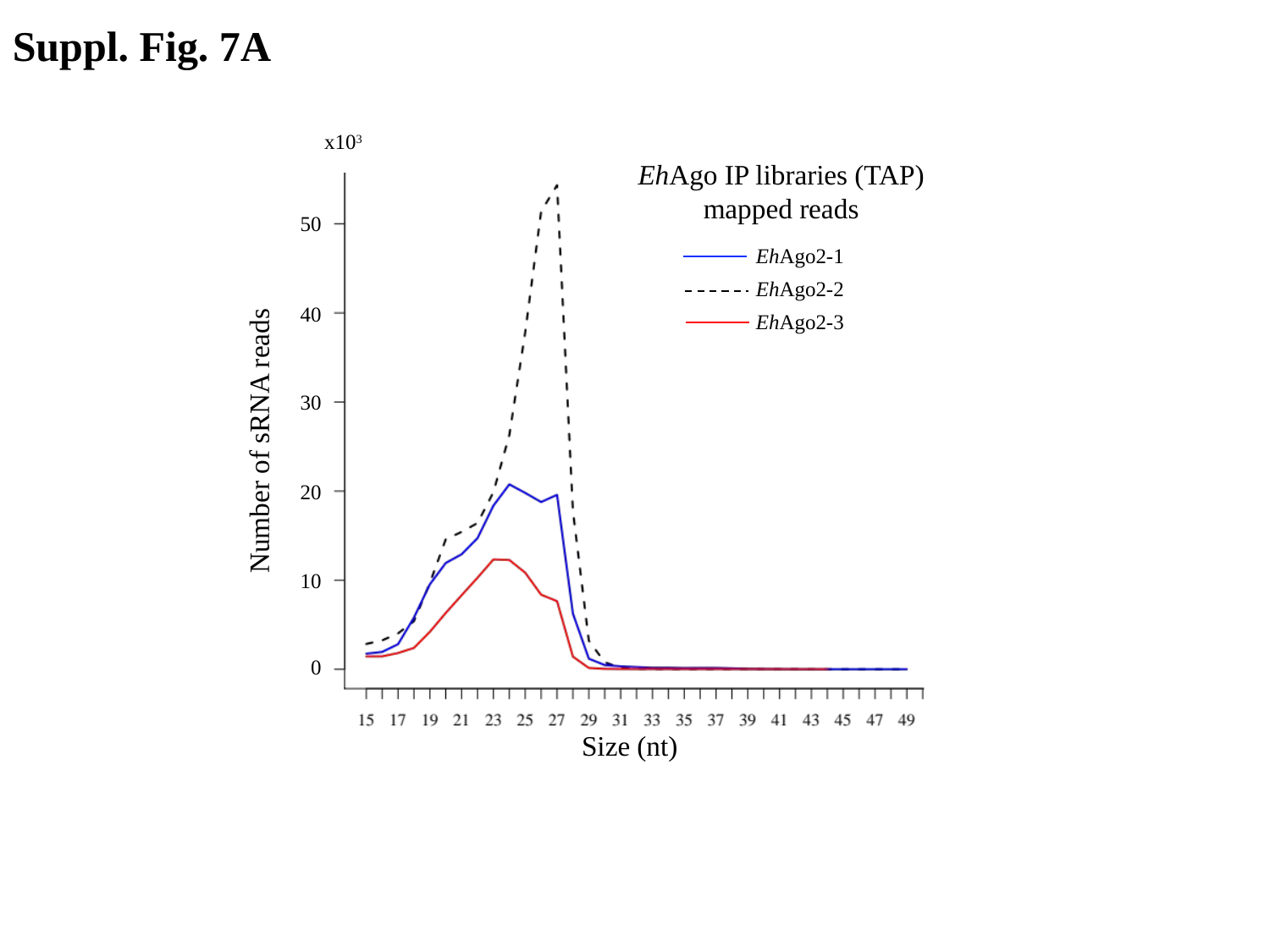

Suppl. Fig. 7A
x103
EhAgo IP libraries (TAP) mapped reads
50
EhAgo2-1
EhAgo2-2
40
EhAgo2-3
Number of sRNA reads
30
20
10
0
Size (nt)

## Slide 14
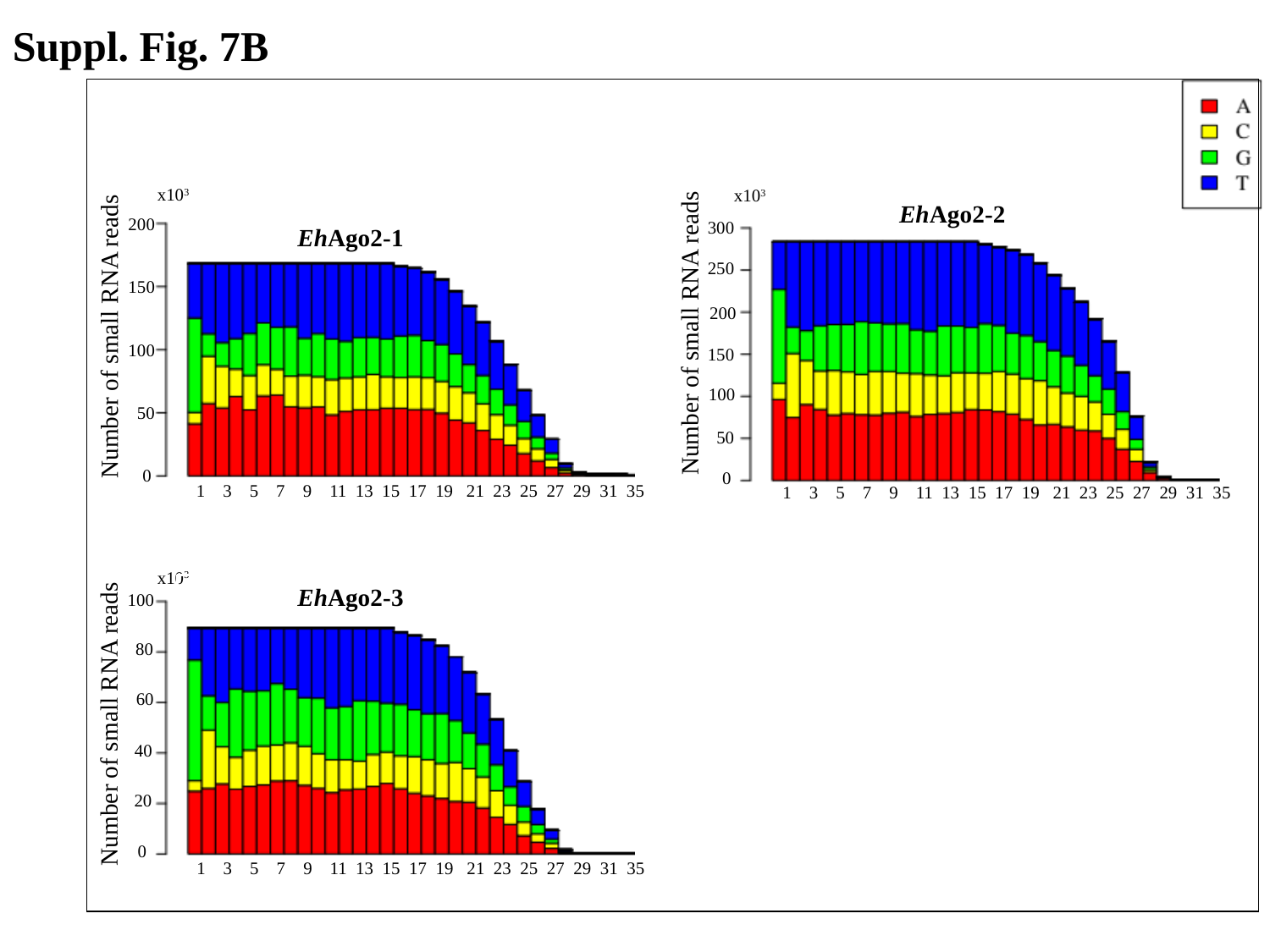

Suppl. Fig. 7B
x103
x103
EhAgo2-2
200
300
EhAgo2-1
250
150
200
Number of small RNA reads
Number of small RNA reads
100
150
100
50
50
0
0
1 3 5 7 9 11 13 15 17 19 21 23 25 27 29 31 35
1 3 5 7 9 11 13 15 17 19 21 23 25 27 29 31 35
x103
EhAgo2-3
100
80
60
Number of small RNA reads
40
20
0
1 3 5 7 9 11 13 15 17 19 21 23 25 27 29 31 35

## Slide 15
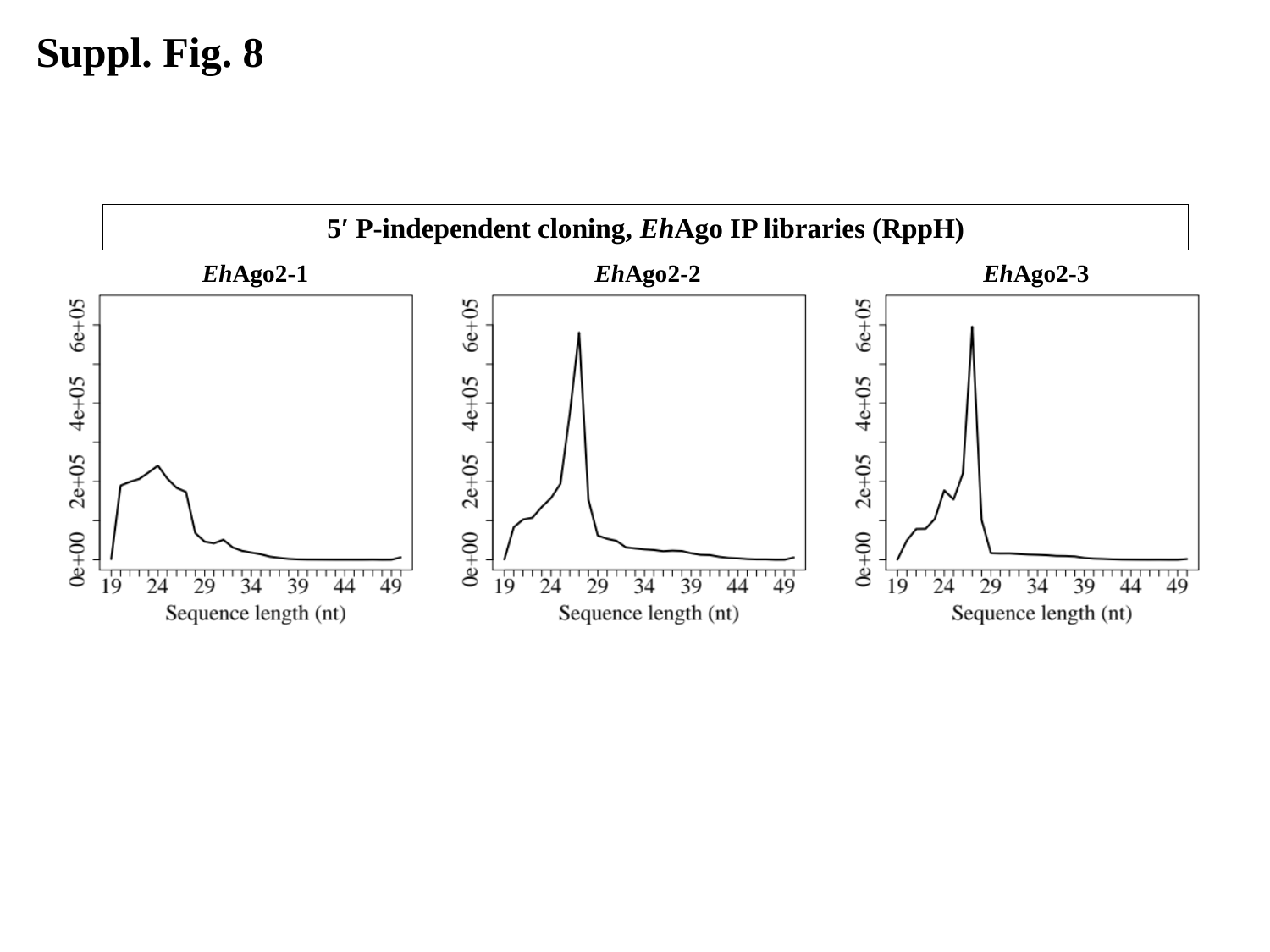

Suppl. Fig. 8
5′ P-independent cloning, EhAgo IP libraries (RppH)
EhAgo2-1
EhAgo2-2
EhAgo2-3

## Slide 16
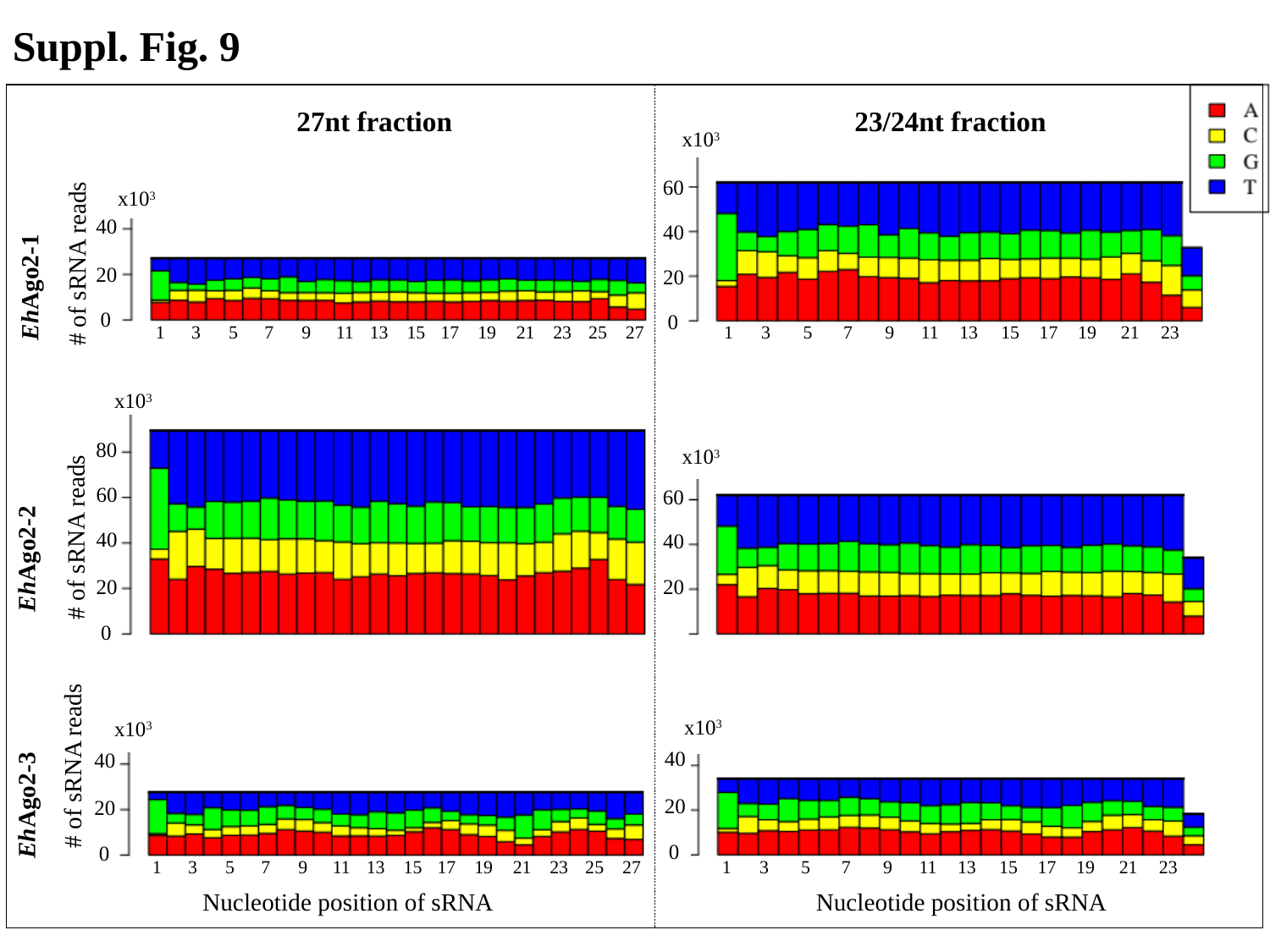

Suppl. Fig. 9
27nt fraction
23/24nt fraction
x103
60
x103
40
40
# of sRNA reads
EhAgo2-1
20
20
0
0
1
3
5
7
9
11
13
15
17
19
21
23
25
27
1
3
5
7
9
11
13
15
17
19
21
23
x103
80
x103
60
60
# of sRNA reads
EhAgo2-2
40
40
20
20
0
x103
x103
# of sRNA reads
40
40
EhAgo2-3
20
20
0
0
1
3
5
7
9
11
13
15
17
19
21
23
25
27
1
3
5
7
9
11
13
15
17
19
21
23
Nucleotide position of sRNA
Nucleotide position of sRNA

## Slide 17
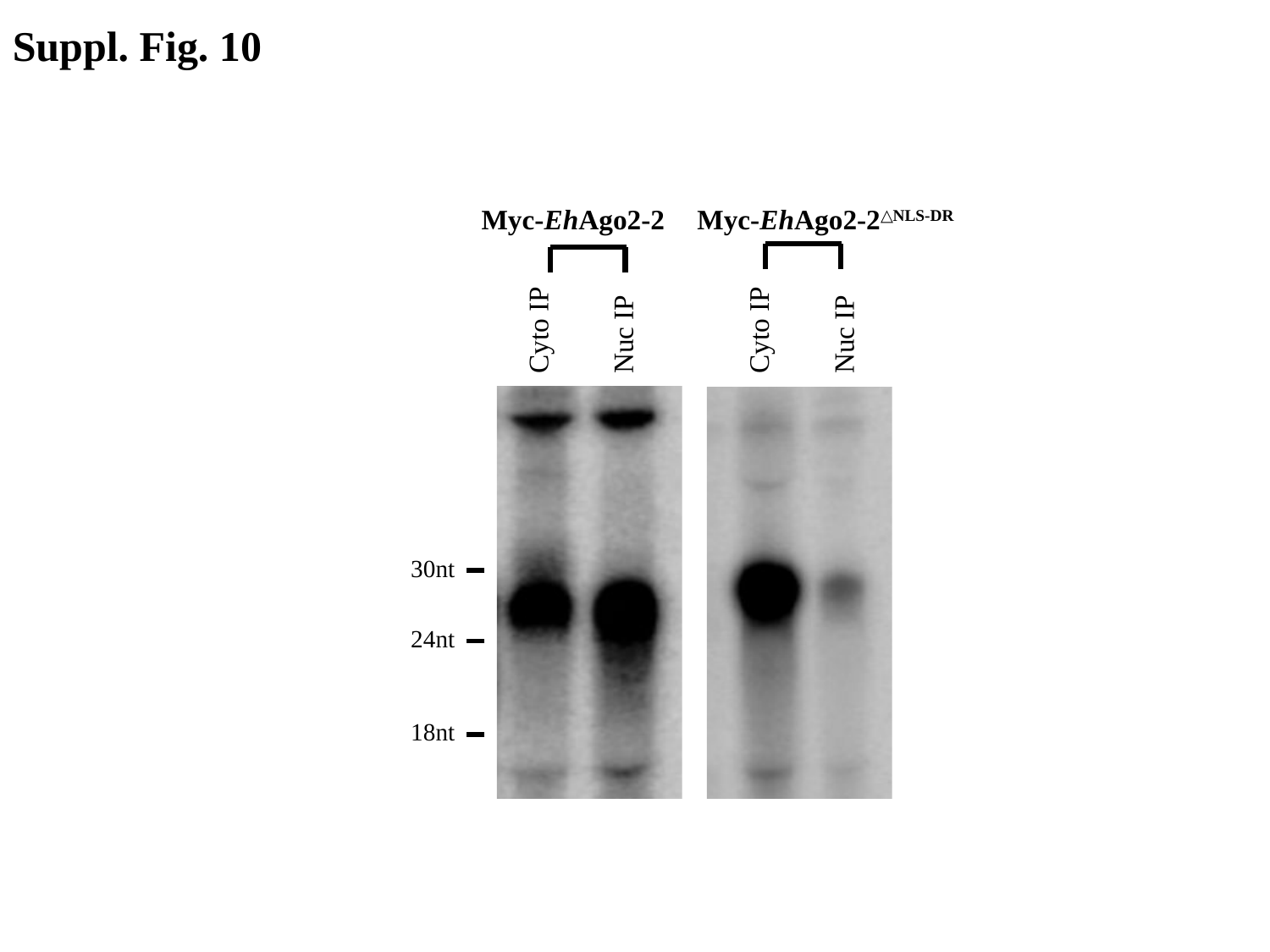

Suppl. Fig. 10
Myc-EhAgo2-2
Myc-EhAgo2-2△NLS-DR
Nuc IP
Nuc IP
Cyto IP
Cyto IP
30nt
24nt
18nt

## Slide 18
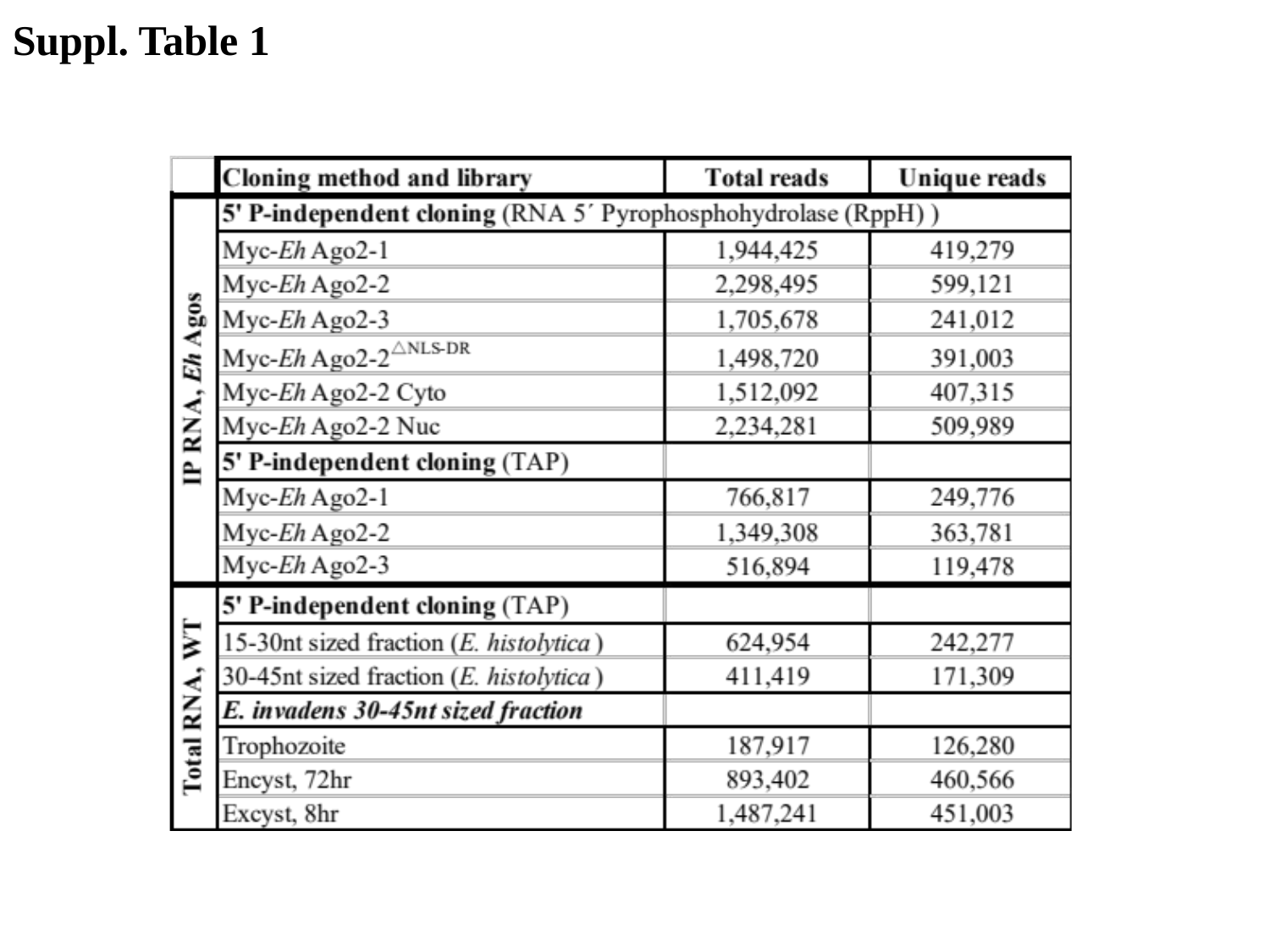

Suppl. Table 1

## Slide 19
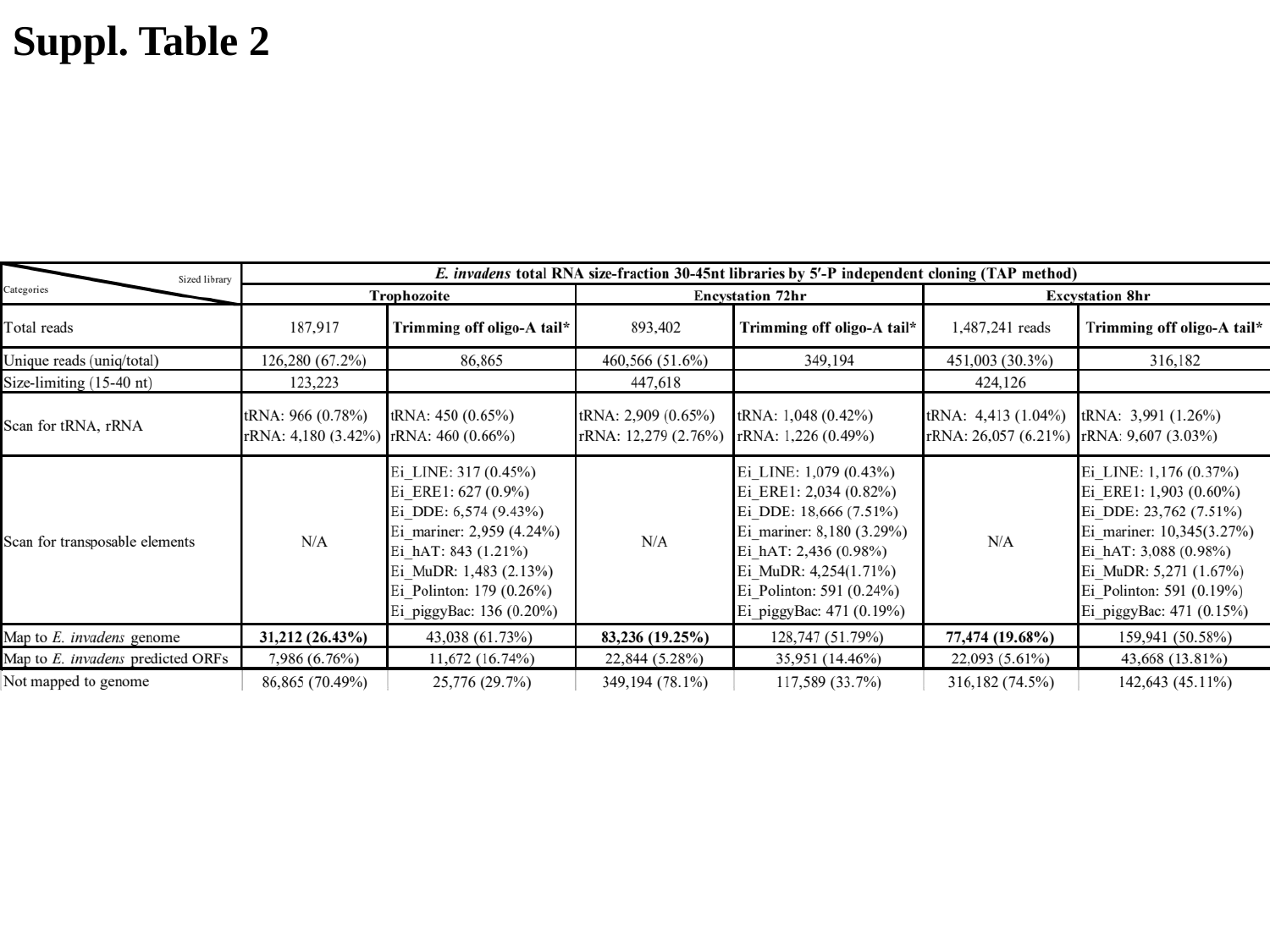

Suppl. Table 2

## Slide 20
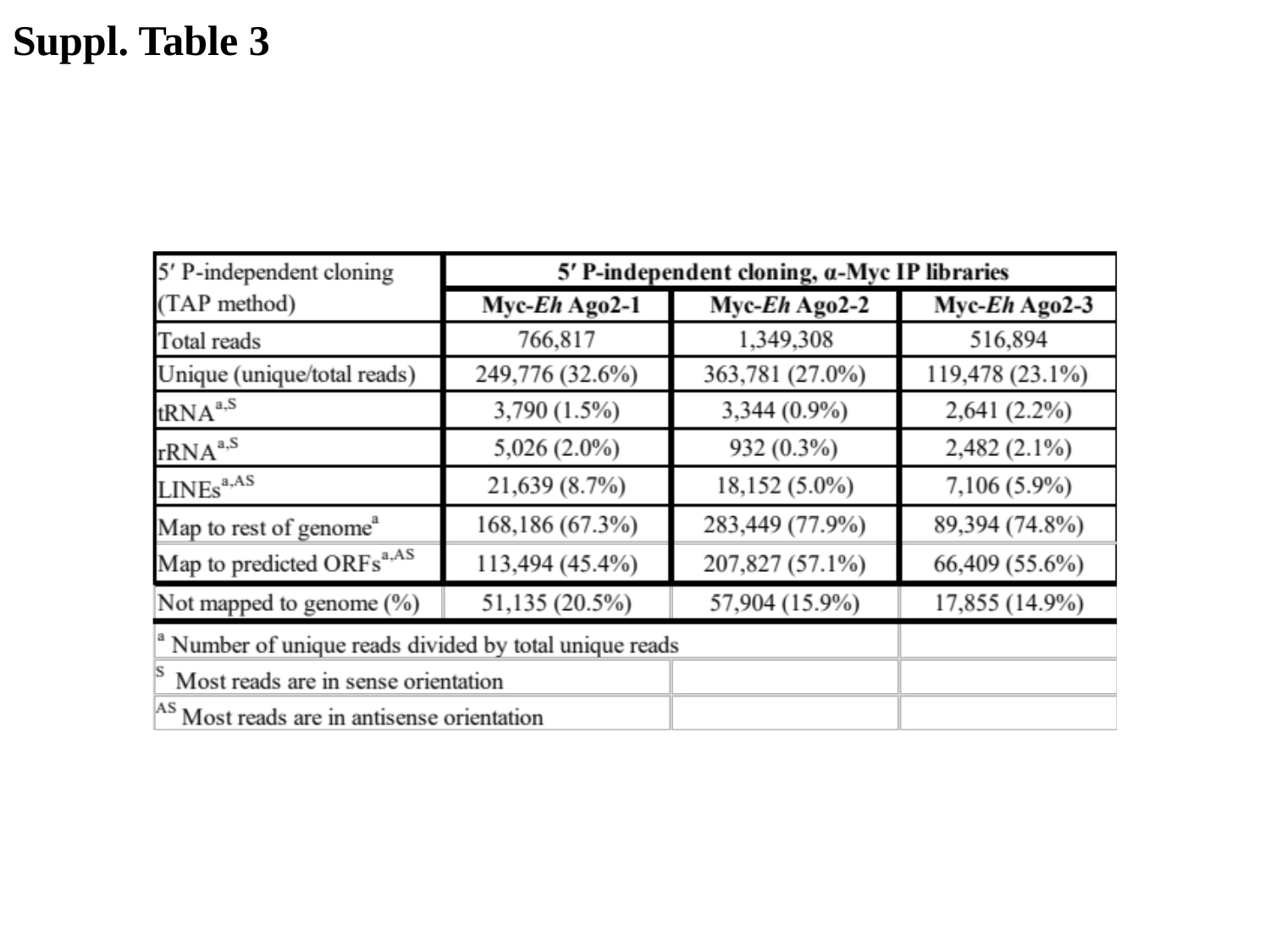

Suppl. Table 3

## Slide 21
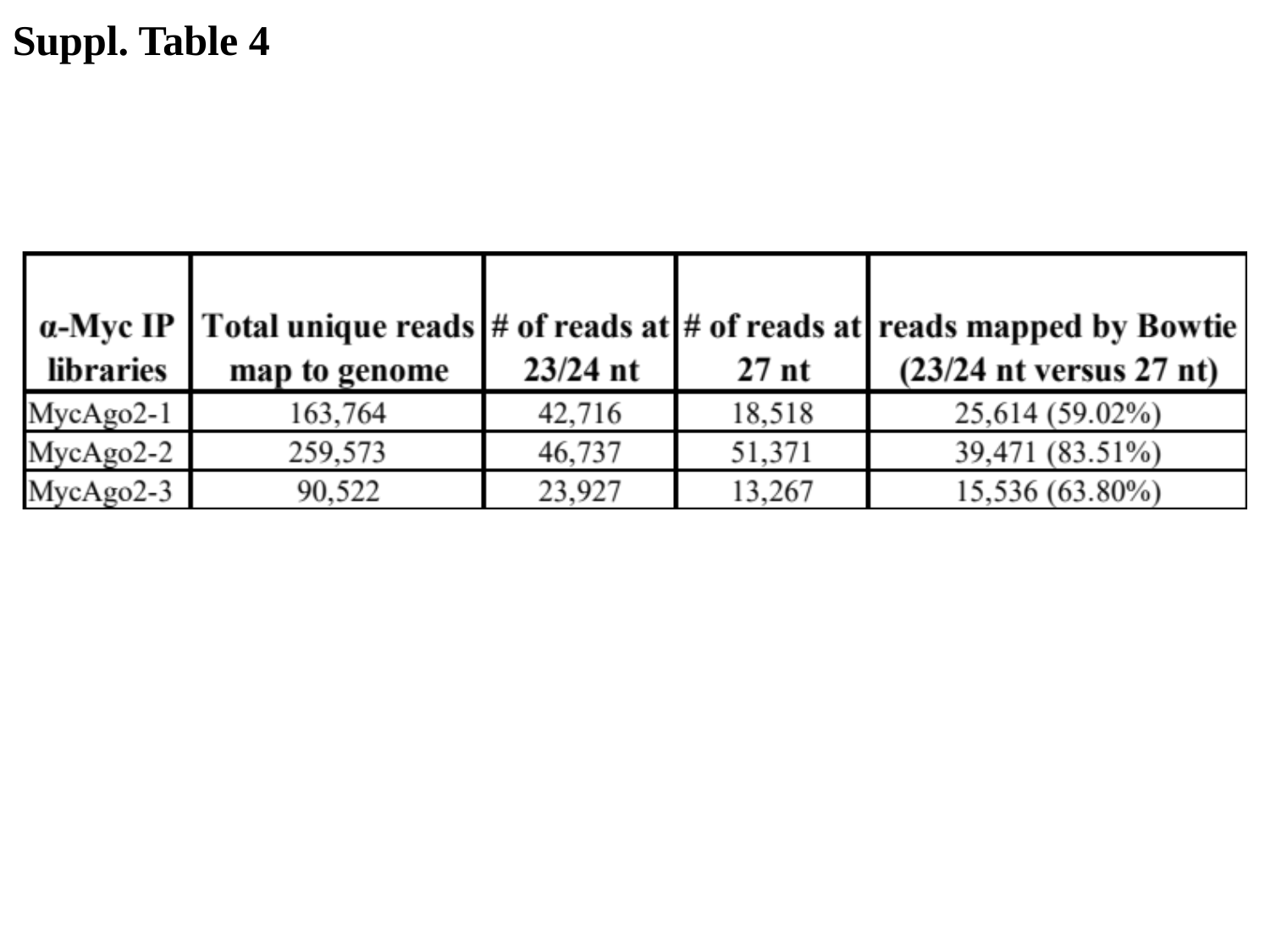

Suppl. Table 4

## Slide 22
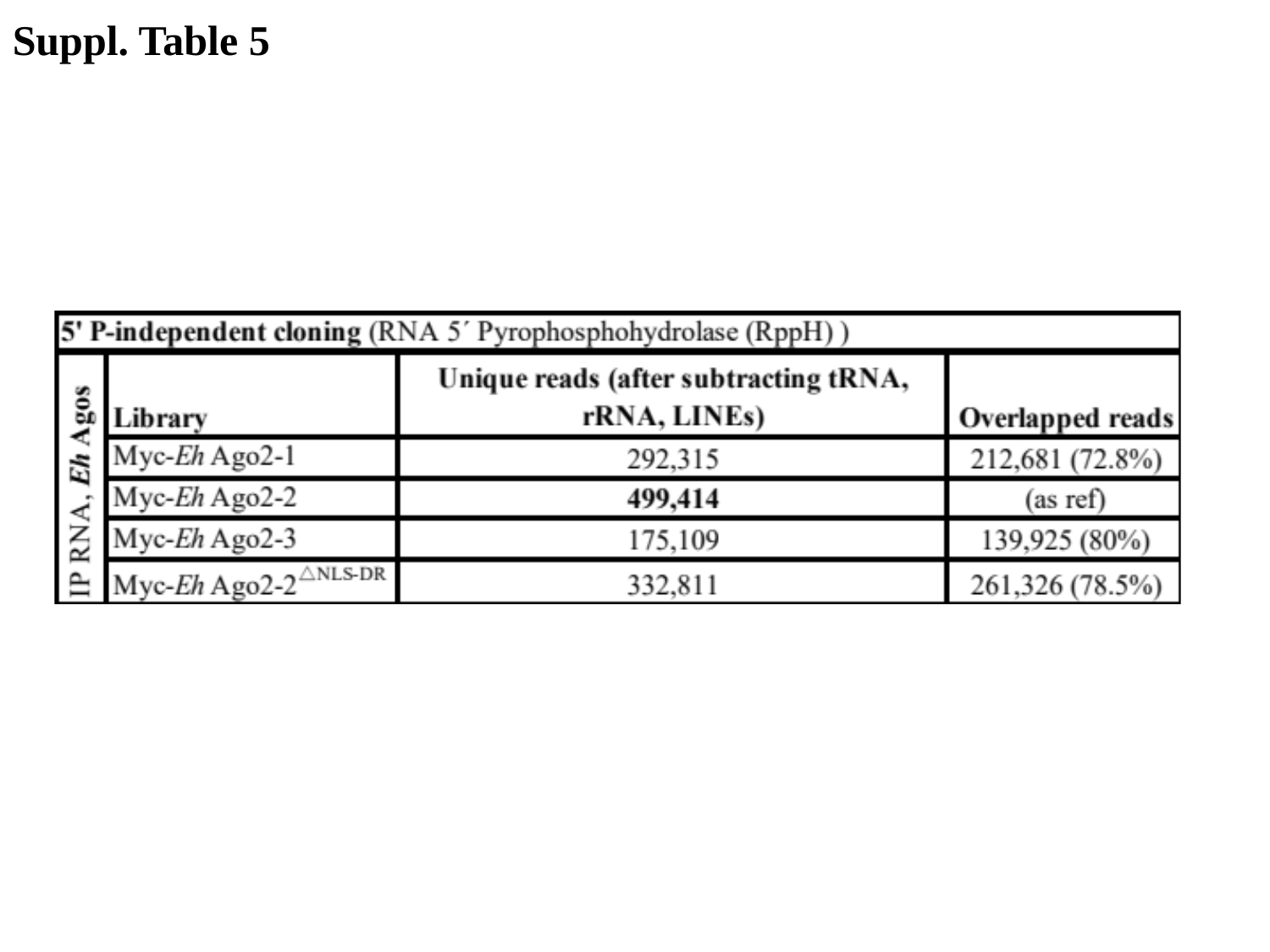

Suppl. Table 5

## Slide 23
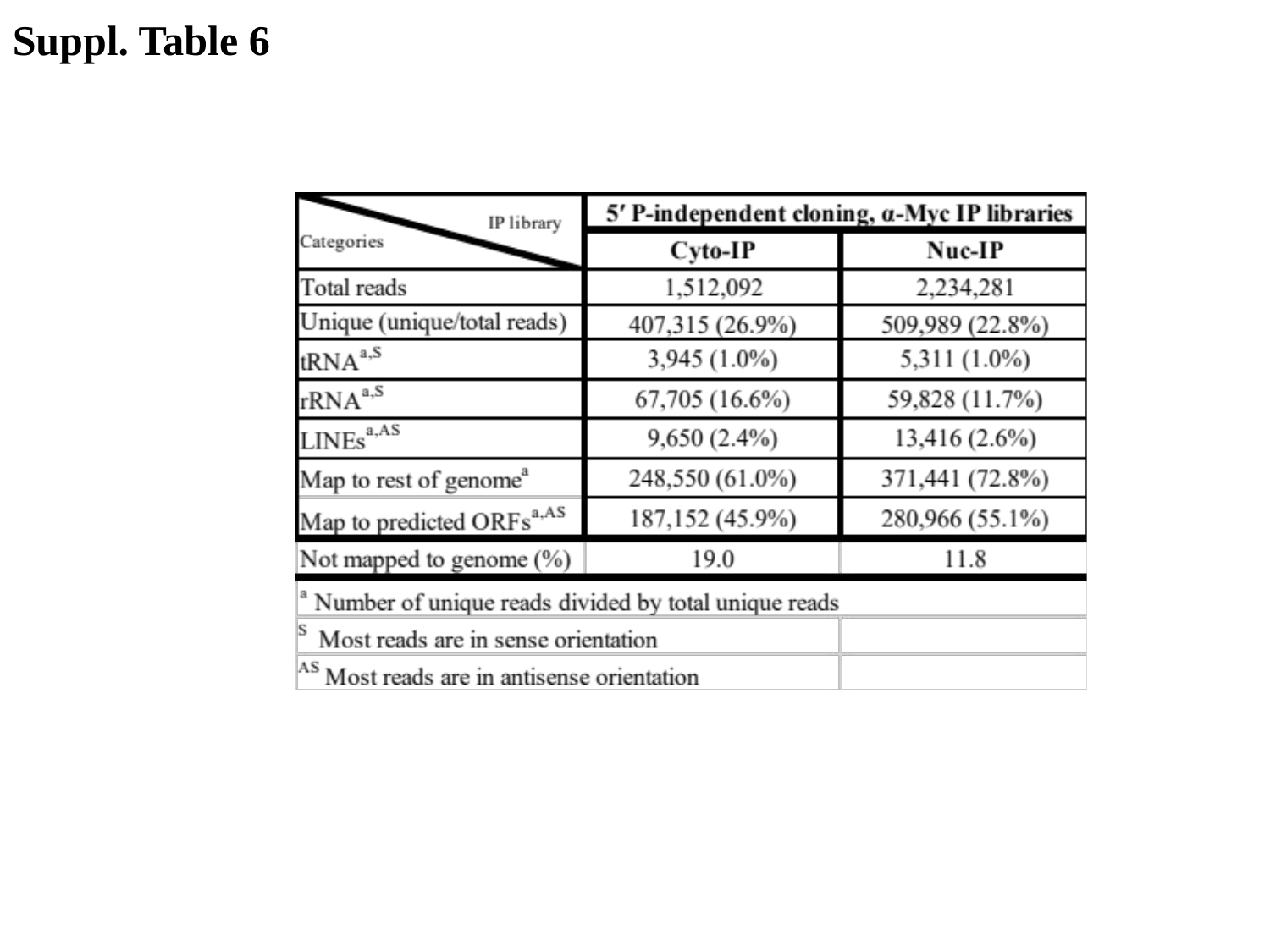

Suppl. Table 6

## Slide 24
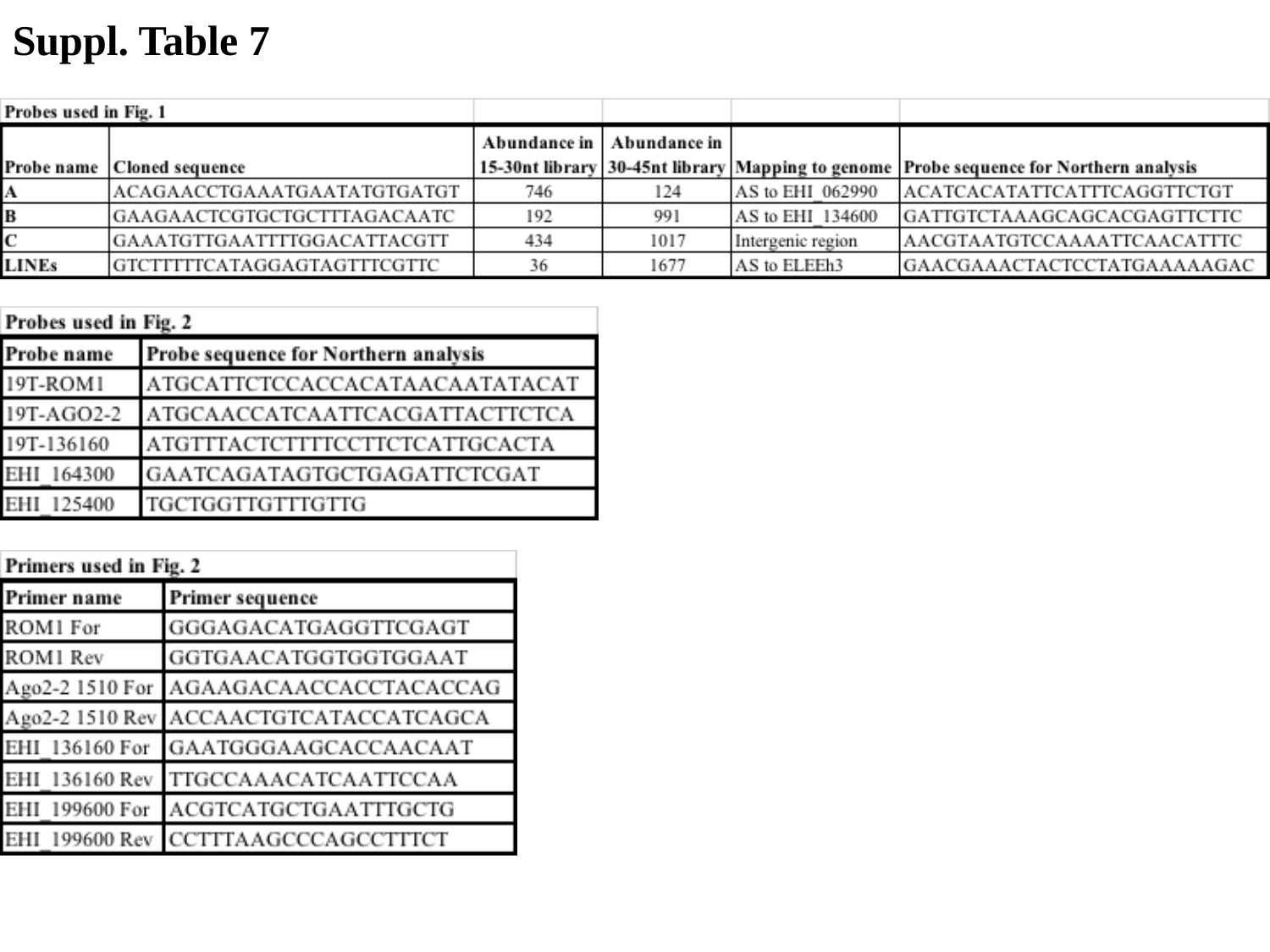

Suppl. Table 7

## Slide 25
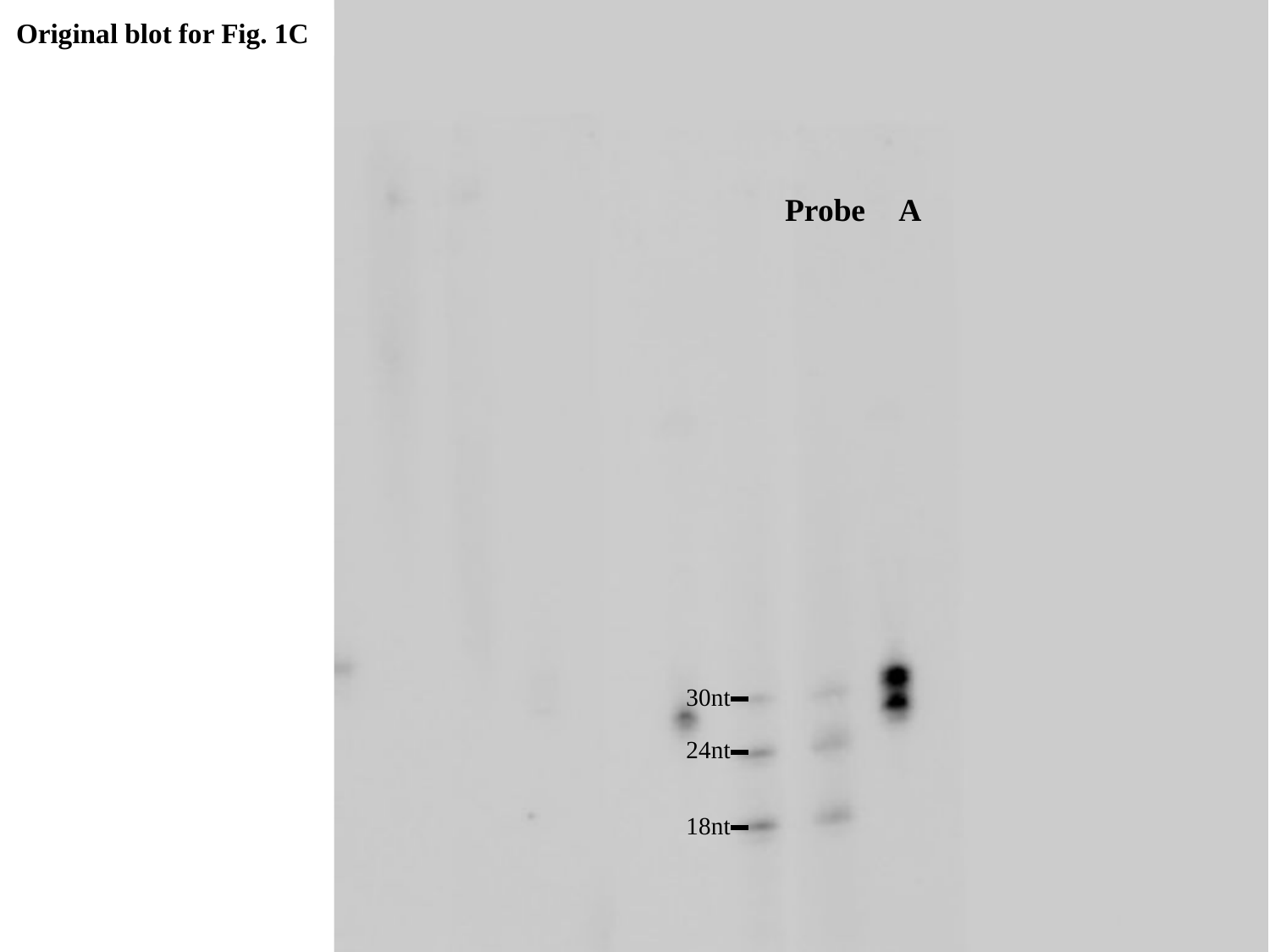

Original blot for Fig. 1C
Probe
A
30nt
24nt
18nt

## Slide 26
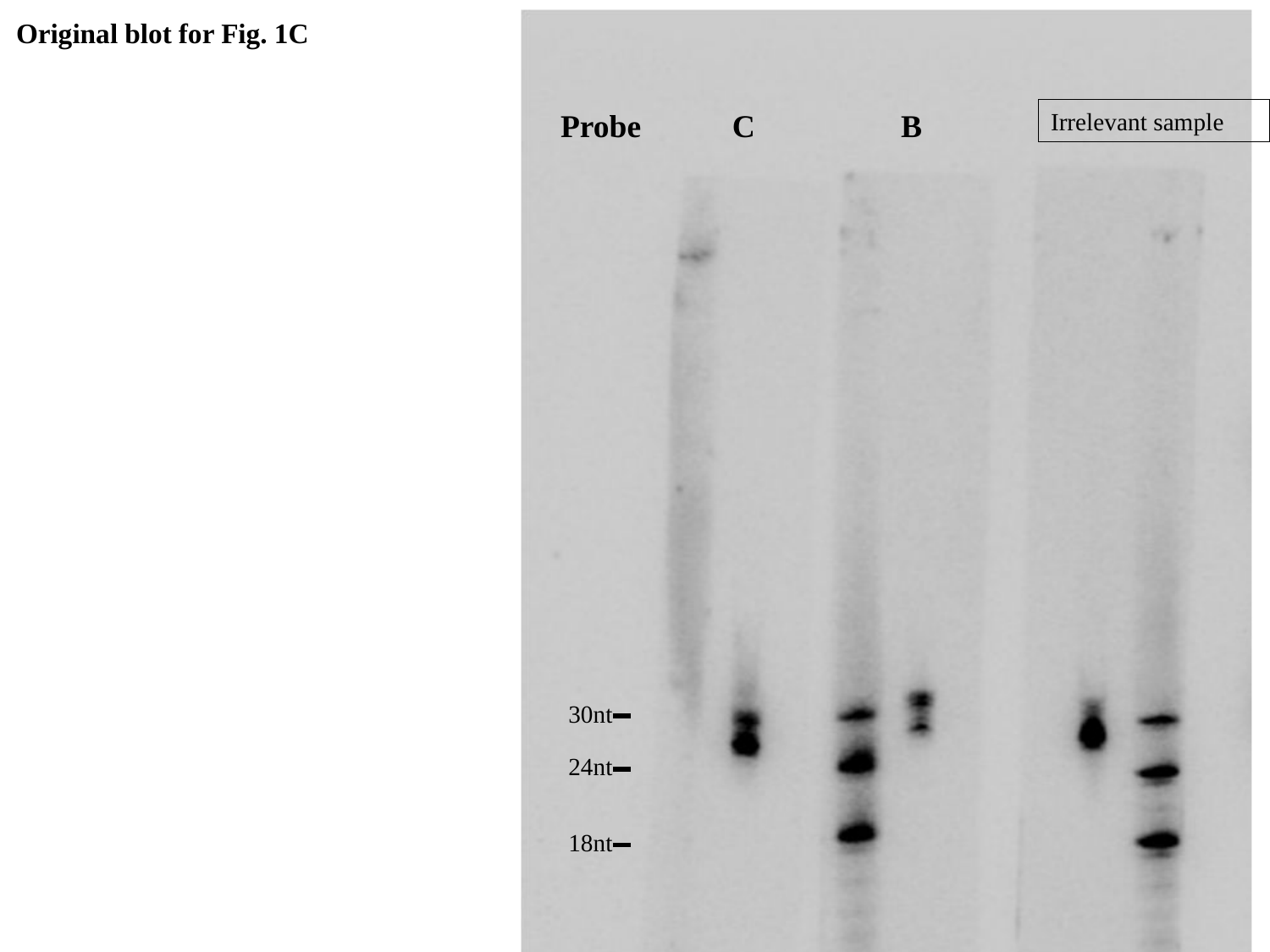

Original blot for Fig. 1C
Probe
C
B
Irrelevant sample
30nt
24nt
18nt

## Slide 27
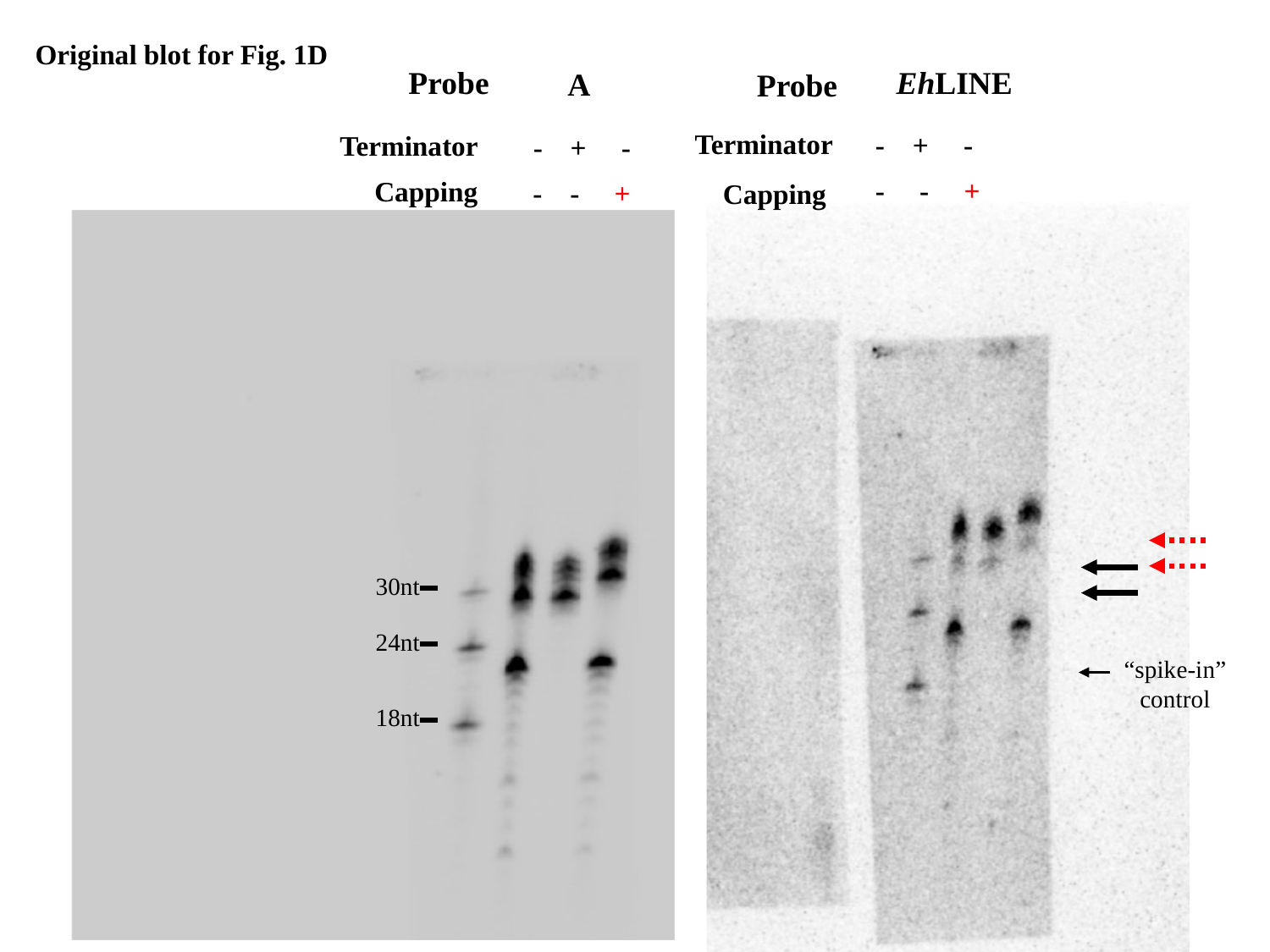

Original blot for Fig. 1D
Probe
EhLINE
A
Probe
Terminator
 - + -
Terminator
 - + -
 - - +
Capping
 - - +
Capping
30nt
24nt
“spike-in” control
18nt

## Slide 28
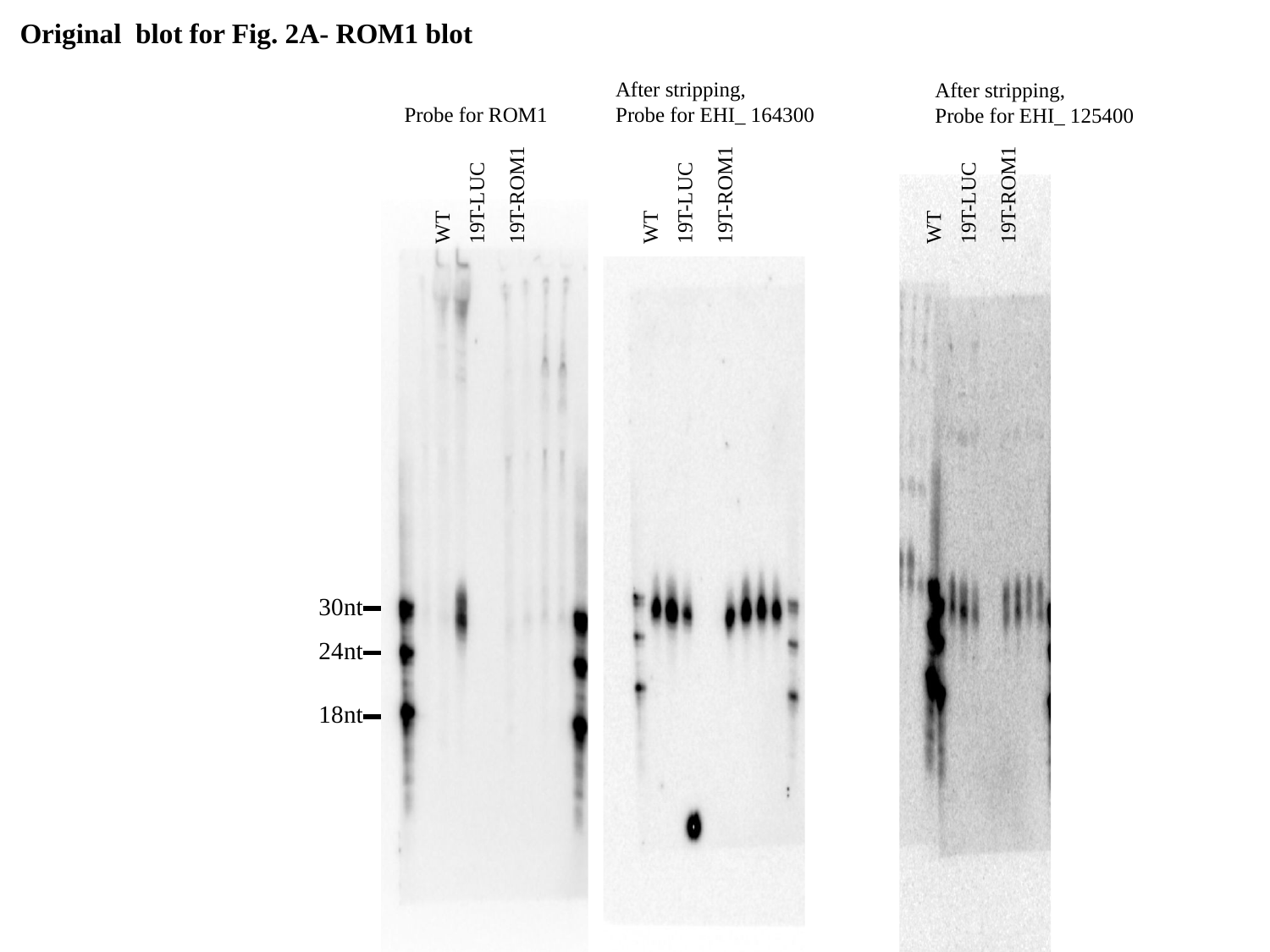

Original blot for Fig. 2A- ROM1 blot
After stripping,
Probe for EHI_ 164300
After stripping,
Probe for EHI_ 125400
Probe for ROM1
19T-LUC
19T-ROM1
19T-LUC
19T-ROM1
19T-LUC
19T-ROM1
WT
WT
WT
30nt
24nt
18nt

## Slide 29
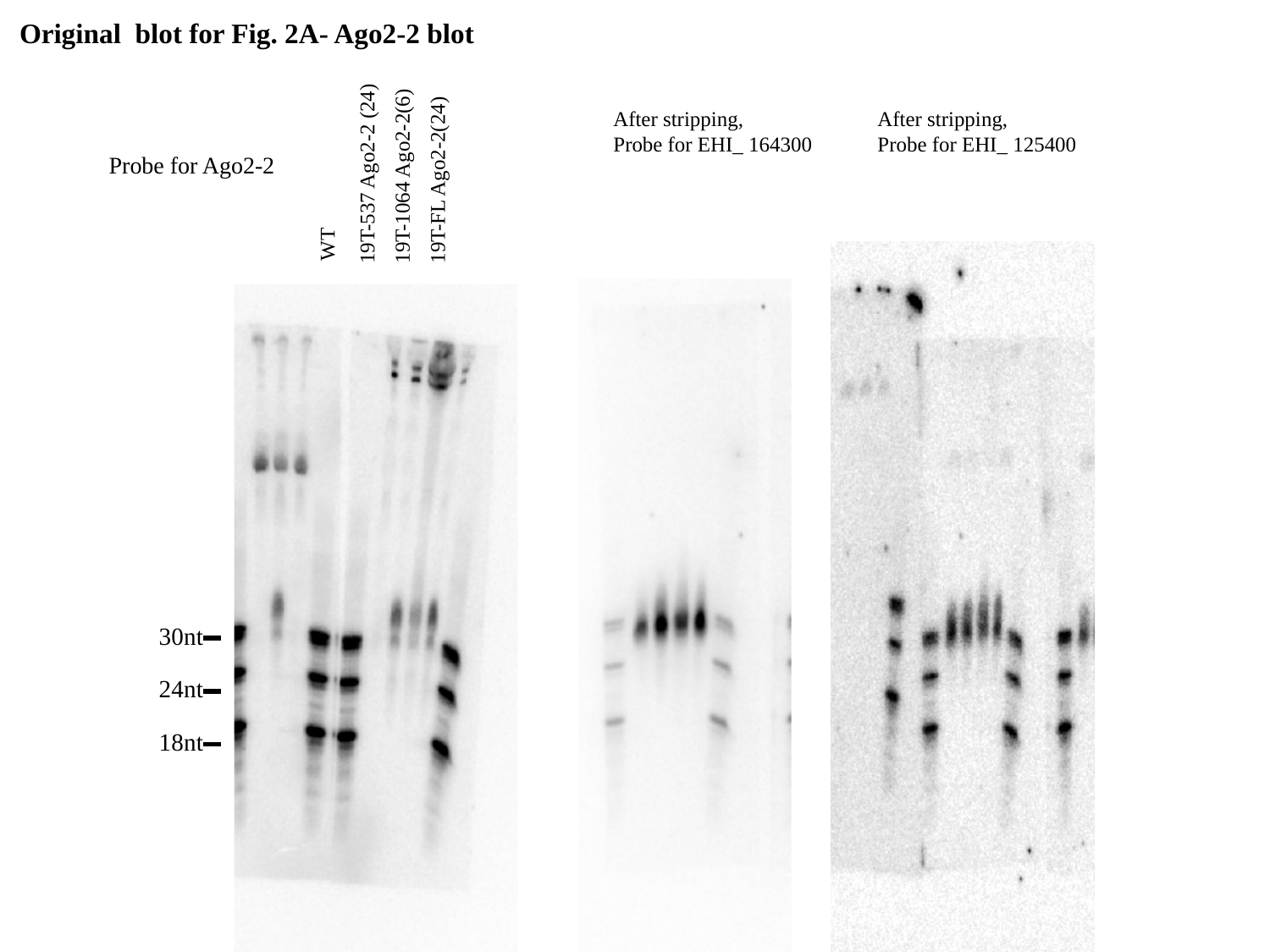

Original blot for Fig. 2A- Ago2-2 blot
After stripping,
Probe for EHI_ 164300
After stripping,
Probe for EHI_ 125400
19T-537 Ago2-2 (24)
Probe for Ago2-2
19T-FL Ago2-2(24)
19T-1064 Ago2-2(6)
WT
30nt
24nt
18nt

## Slide 30
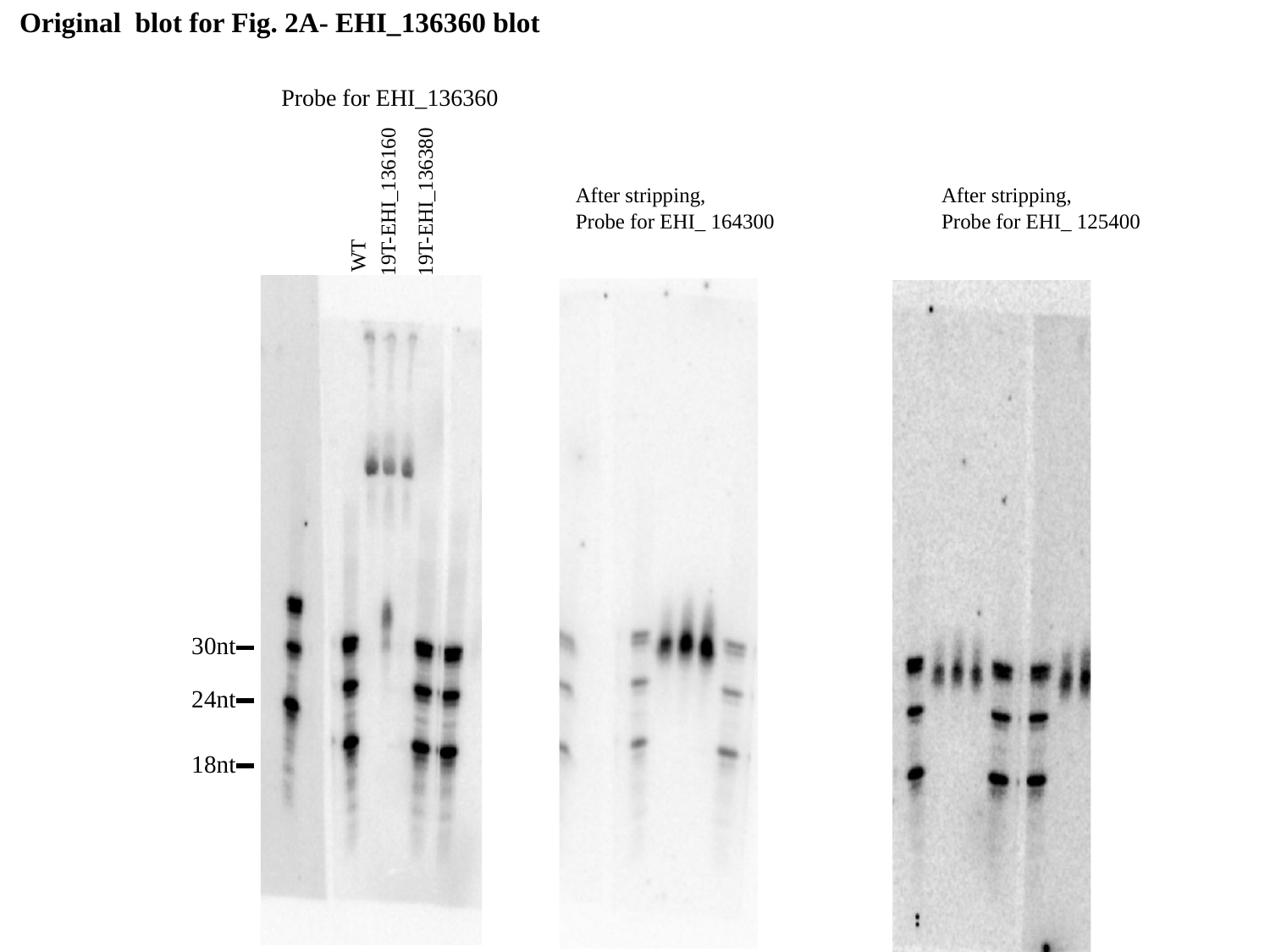

Original blot for Fig. 2A- EHI_136360 blot
Probe for EHI_136360
19T-EHI_136160
19T-EHI_136380
After stripping,
Probe for EHI_ 164300
After stripping,
Probe for EHI_ 125400
WT
30nt
24nt
18nt

## Slide 31
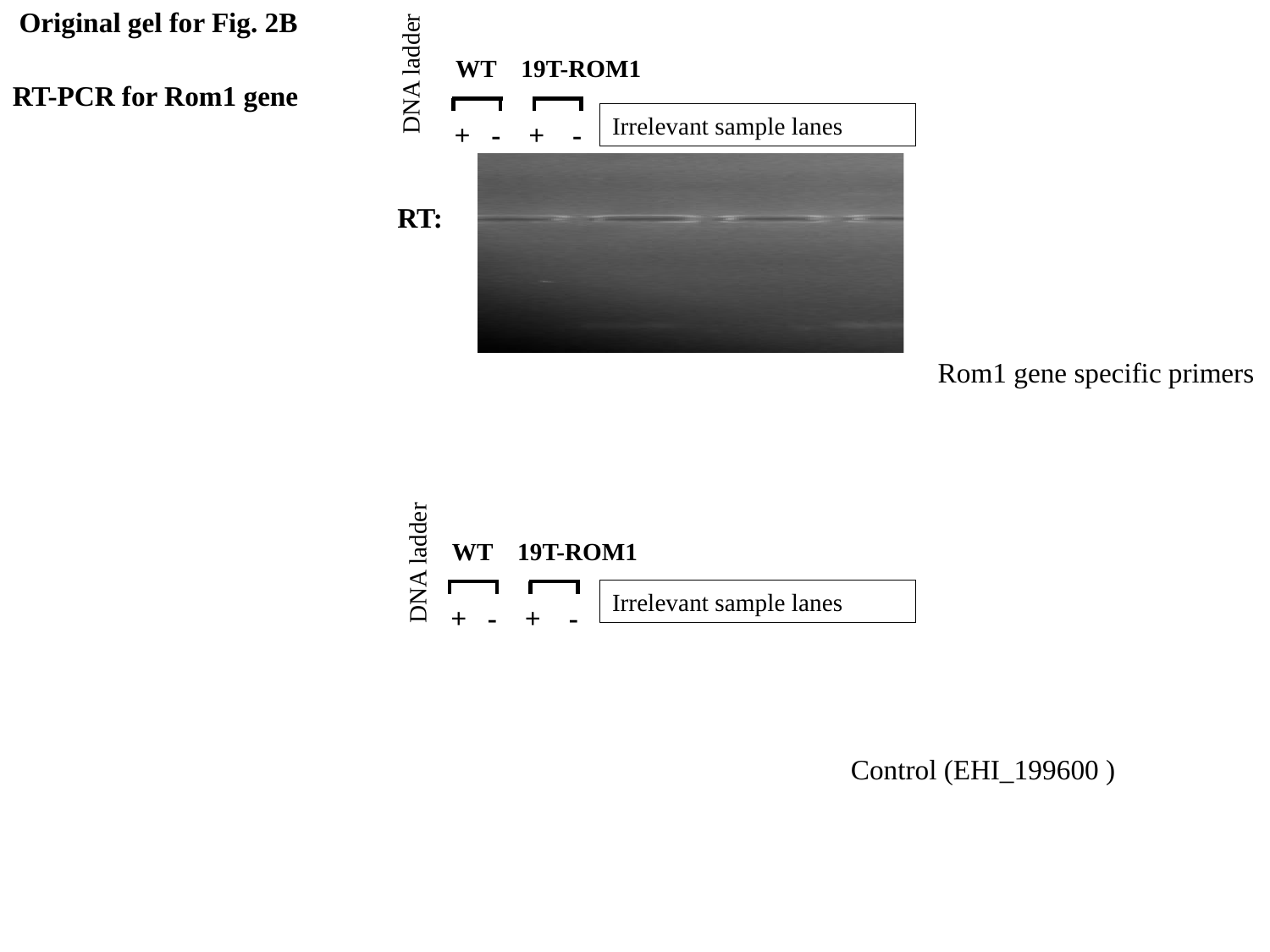

Original gel for Fig. 2B
DNA ladder
WT 19T-ROM1
RT-PCR for Rom1 gene
Irrelevant sample lanes
+ - + -
RT:
Rom1 gene specific primers
WT 19T-ROM1
DNA ladder
Irrelevant sample lanes
+ - + -
Control (EHI_199600 )

## Slide 32
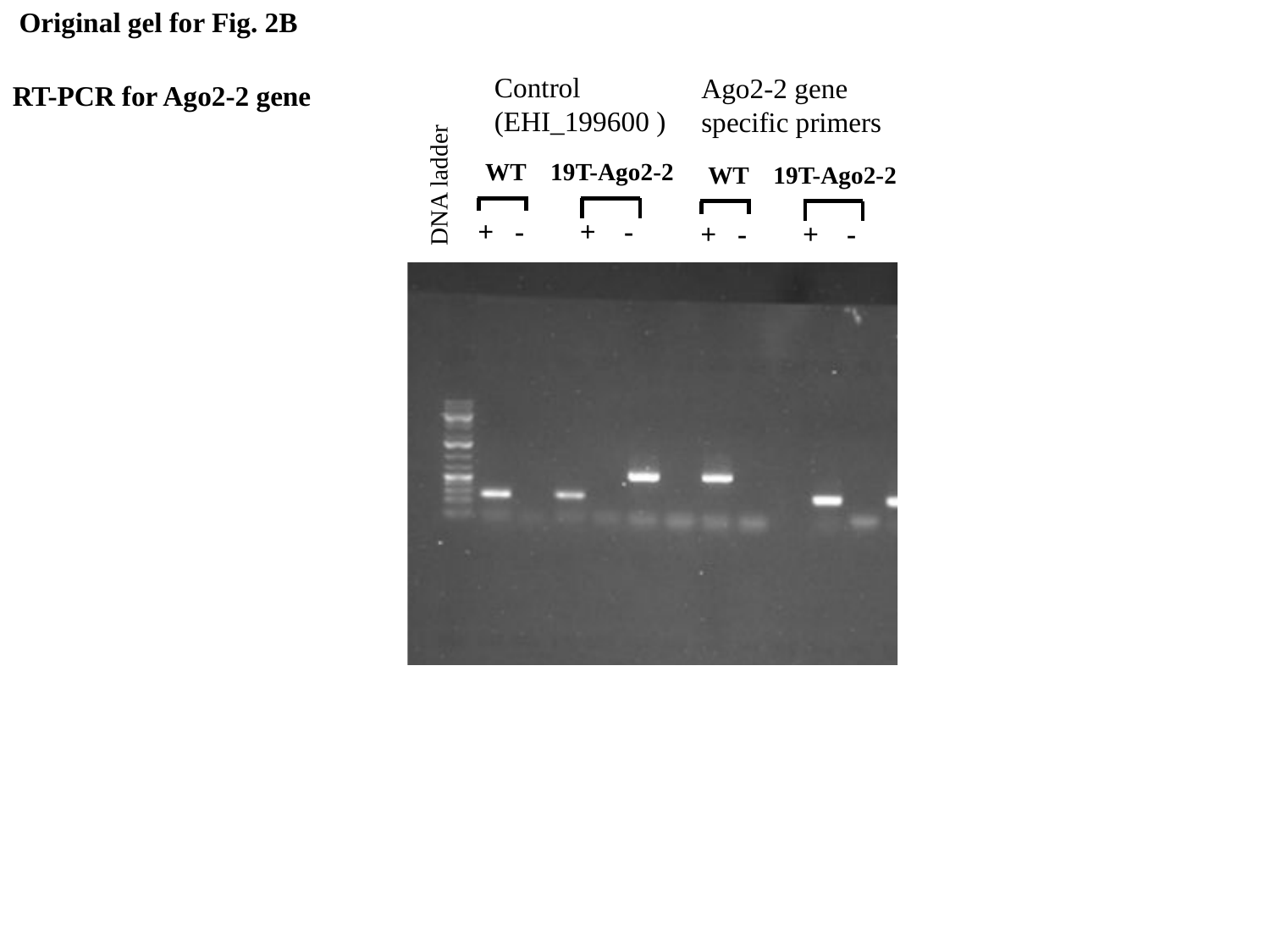

Original gel for Fig. 2B
Control (EHI_199600 )
Ago2-2 gene specific primers
RT-PCR for Ago2-2 gene
WT 19T-Ago2-2
WT 19T-Ago2-2
DNA ladder
+ - + -
+ - + -

## Slide 33
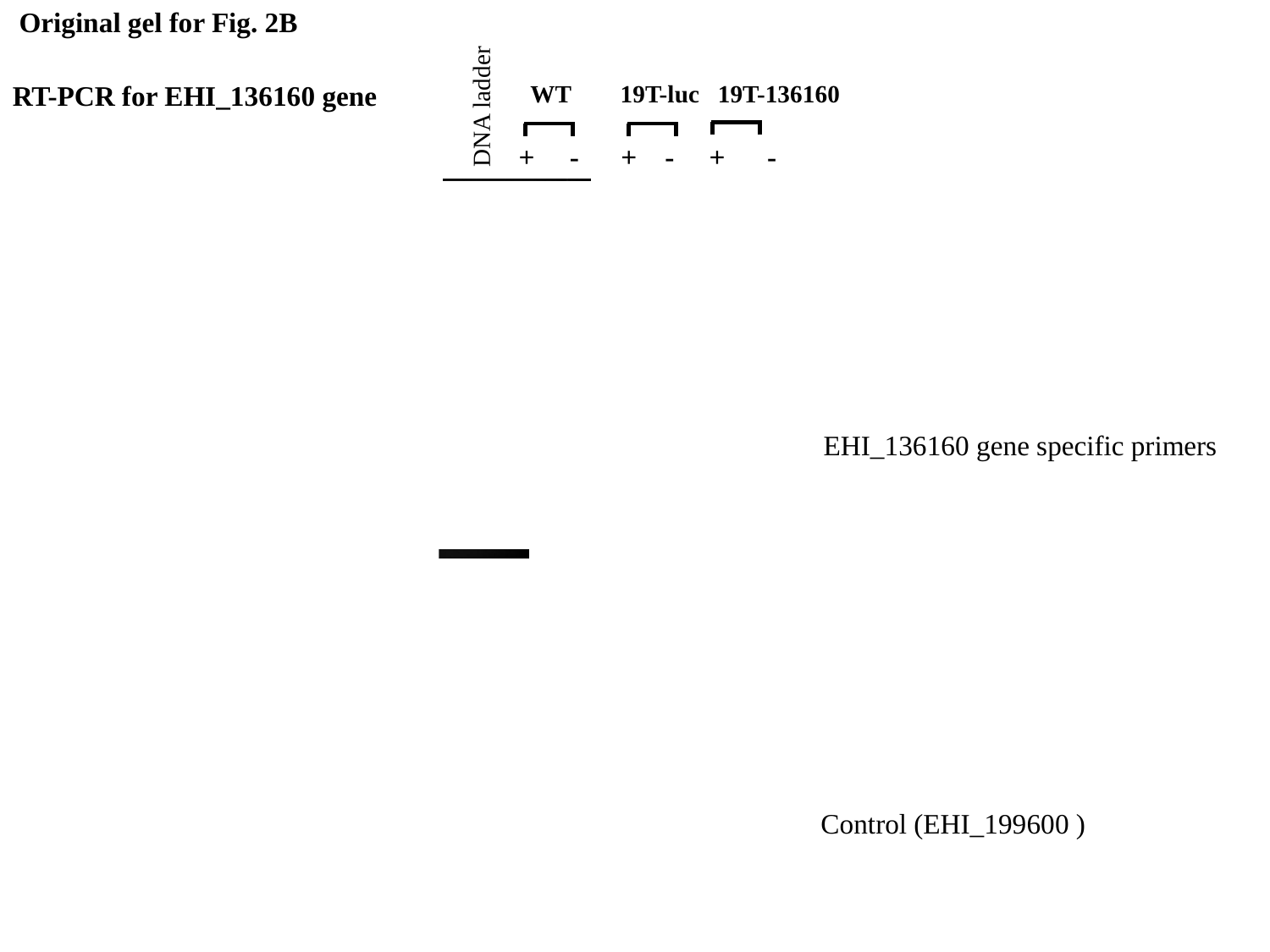

Original gel for Fig. 2B
RT-PCR for EHI_136160 gene
 WT 19T-luc 19T-136160
DNA ladder
+ - + - + -
EHI_136160 gene specific primers
Control (EHI_199600 )

## Slide 34
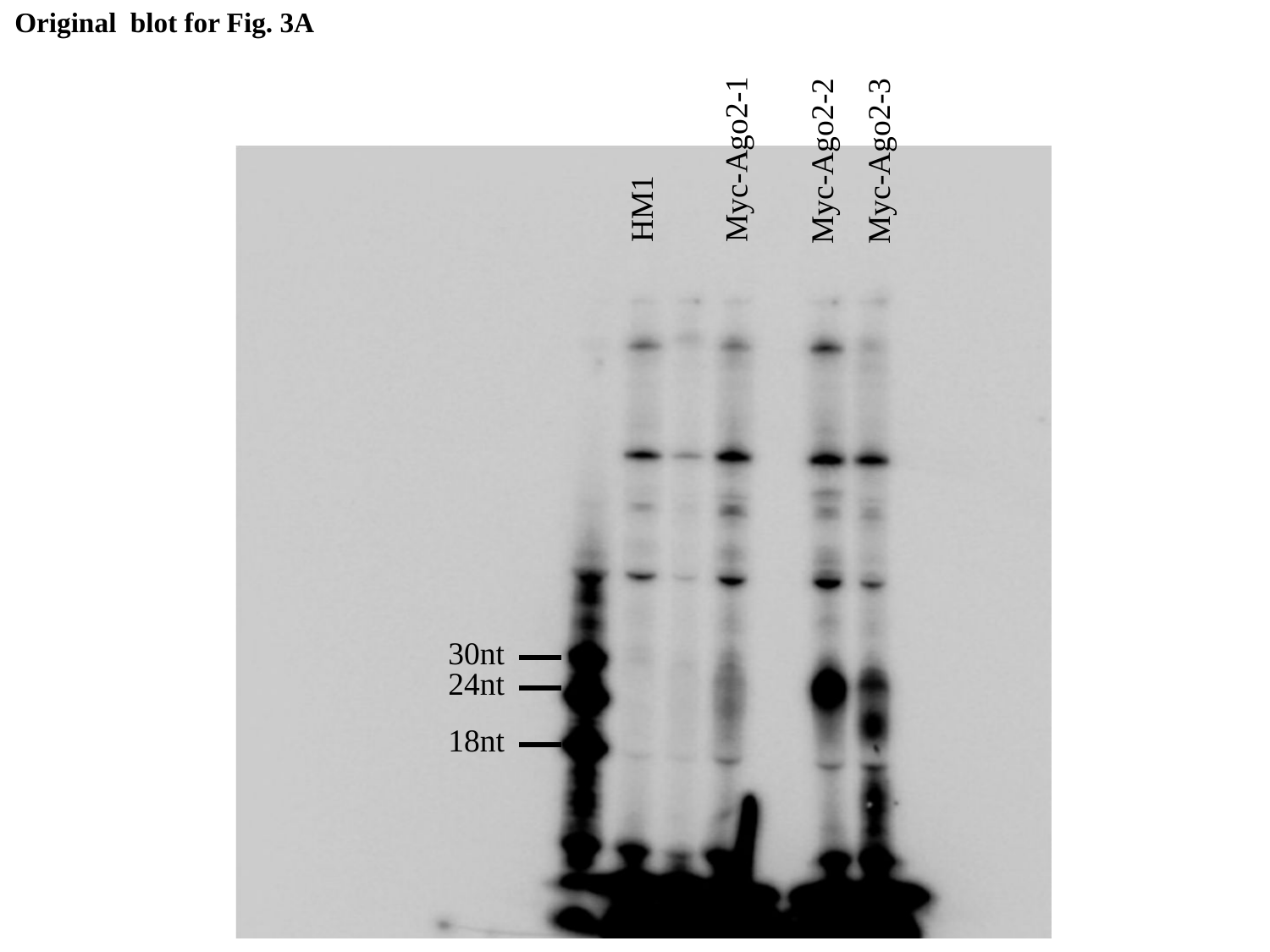

Original blot for Fig. 3A
Myc-Ago2-1
Myc-Ago2-2
Myc-Ago2-3
HM1
30nt
24nt
18nt

## Slide 35
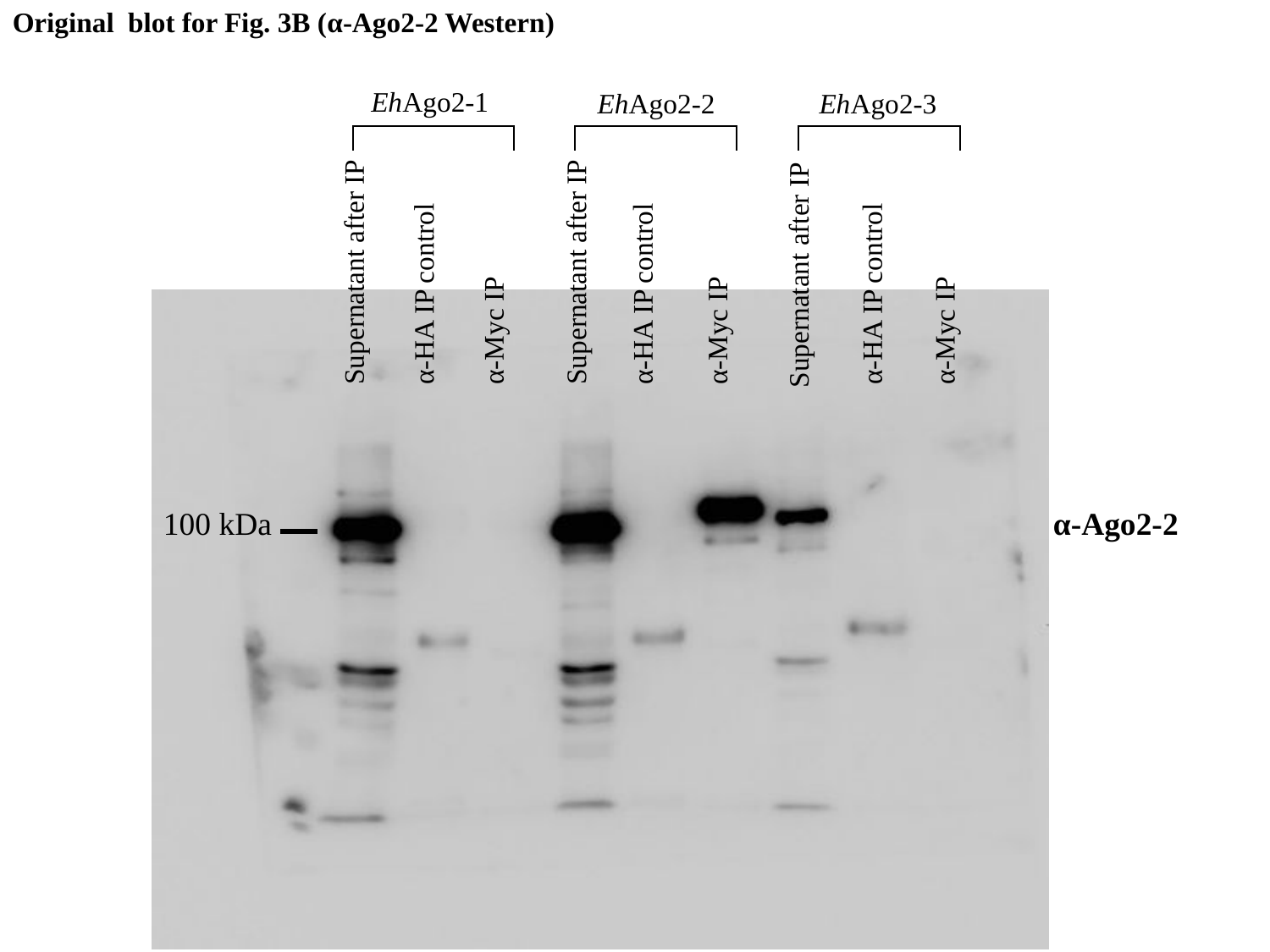

Original blot for Fig. 3B (α-Ago2-2 Western)
EhAgo2-1
EhAgo2-2
EhAgo2-3
Supernatant after IP
Supernatant after IP
Supernatant after IP
α-HA IP control
α-HA IP control
α-HA IP control
α-Myc IP
α-Myc IP
α-Myc IP
100 kDa
α-Ago2-2

## Slide 36
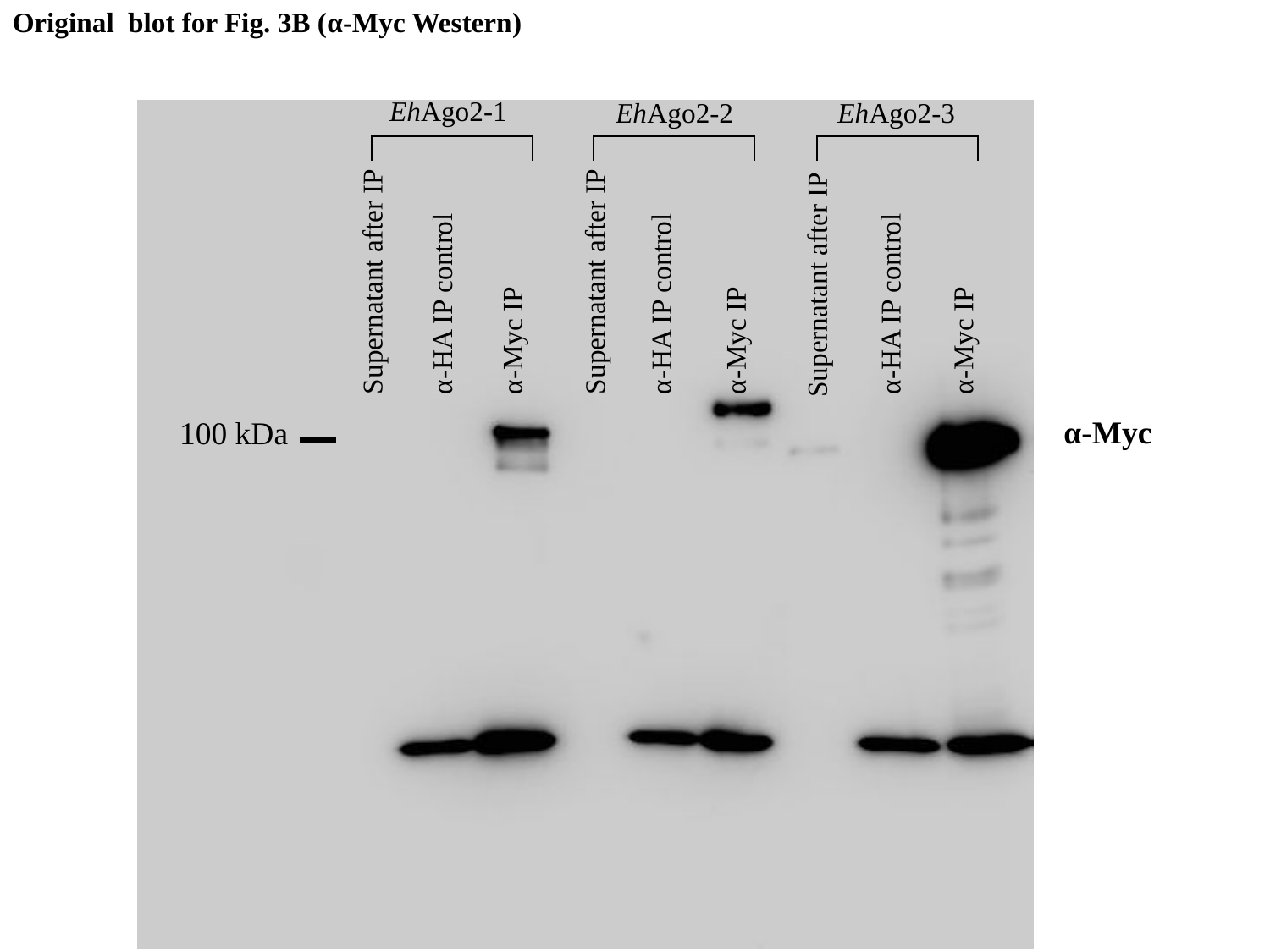

Original blot for Fig. 3B (α-Myc Western)
EhAgo2-1
EhAgo2-2
EhAgo2-3
Supernatant after IP
Supernatant after IP
Supernatant after IP
α-HA IP control
α-HA IP control
α-HA IP control
α-Myc IP
α-Myc IP
α-Myc IP
α-Myc
100 kDa

## Slide 37
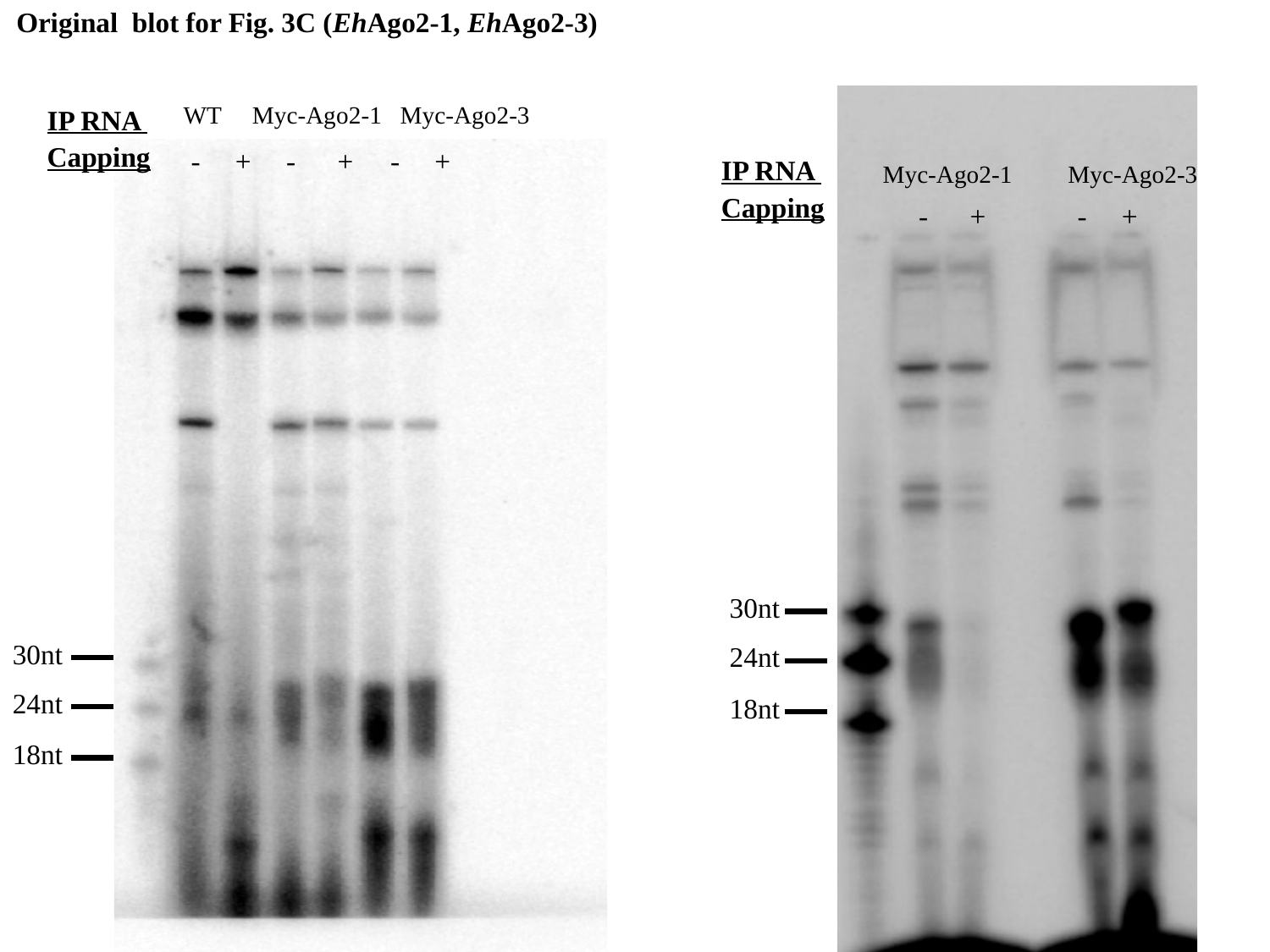

Original blot for Fig. 3C (EhAgo2-1, EhAgo2-3)
WT
Myc-Ago2-1
Myc-Ago2-3
IP RNA
Capping
 - +
 - +
 - +
IP RNA
Myc-Ago2-1
Myc-Ago2-3
Capping
 - +
 - +
30nt
30nt
24nt
24nt
18nt
18nt

## Slide 38
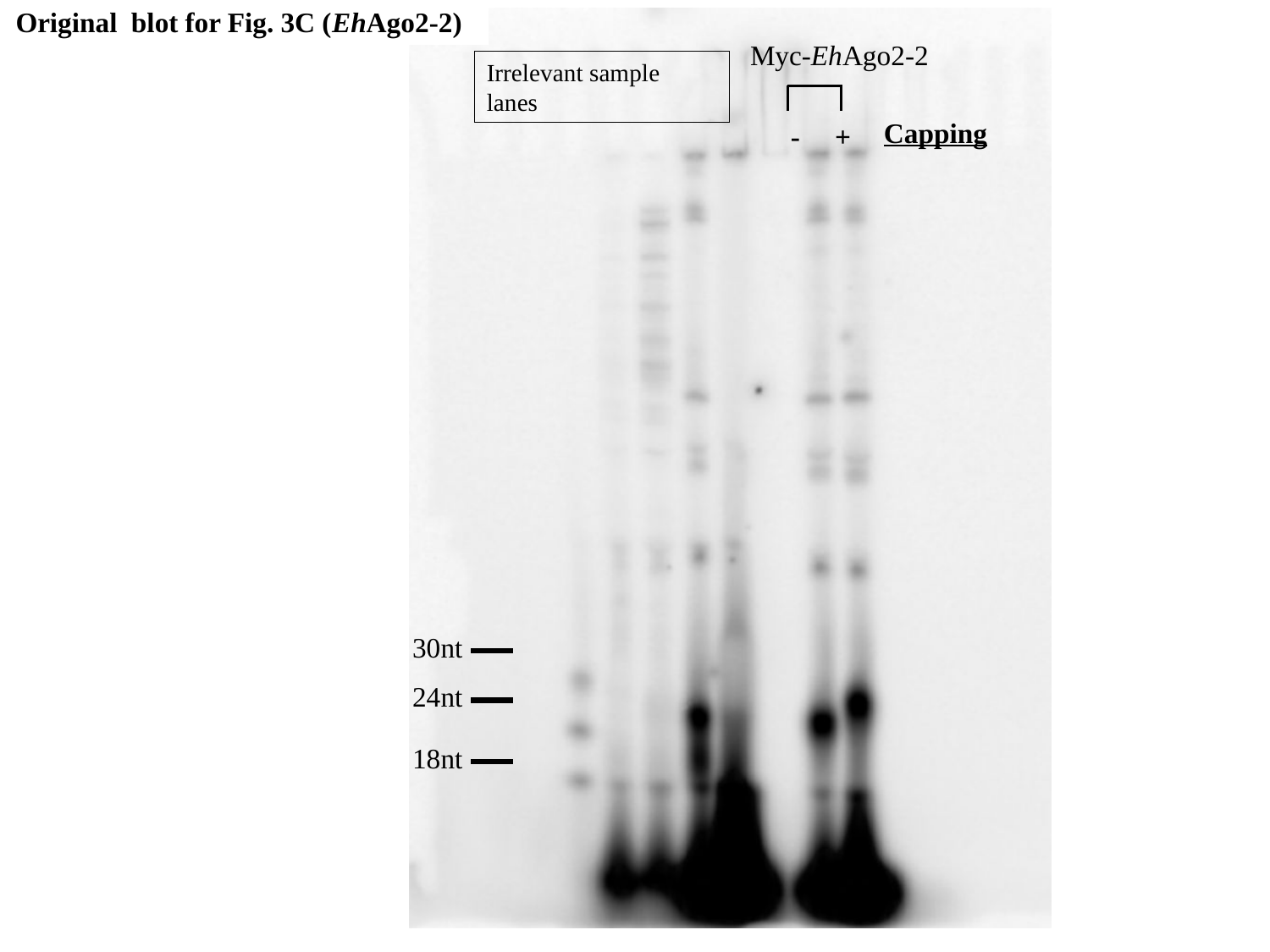

Original blot for Fig. 3C (EhAgo2-2)
Myc-EhAgo2-2
Irrelevant sample lanes
Capping
 - +
30nt
24nt
18nt
